# Vinyl Cation Stabilization by Silicon Enables a Formal Metal‐Free α‐Arylation of Alkyl Ketones

**DOI:** 10.1002/anie.201909381

**Published:** 2019-10-22

**Authors:** Amandine Pons, Jean Michalland, Wojciech Zawodny, Yong Chen, Veronica Tona, Nuno Maulide

**Affiliations:** ^1^ Institute of Organic Chemistry University of Vienna Währinger Strasse 38 1090 Vienna Austria

**Keywords:** α-arylation, sigmatropic rearrangement, silicon β-effect, vinyl cations

## Abstract

The ability of silicon to stabilize vinyl cationic species leads to a redox arylation of alkynes whereby the stringent limitations of reactivity and regioselectivity of alkyl‐substituted alkynes are lifted. This allows the synthesis of a range of α‐silyl‐α′‐arylketones under mild conditions in good to excellent yields and with high functional group tolerance, whereby the silicon moiety in the final products can either be removed for a formal acetone monoarylation transform, or capitalized upon for subsequent electrophilic substitutions at either side of the carbonyl group.

## Graphical abstract

**I can Si clearly now**: The ability of silicon to stabilize vinyl cationic species enables a redox arylation of alkynes whereby the poor reactivity and regioselectivity of alkyl‐substituted alkynes are lifted. This enables the synthesis of a range of α‐silyl‐α′‐arylketones under mild conditions in good to excellent yields. The silicon moiety of the products can either be removed or harnessed for additional C−C bond formation.

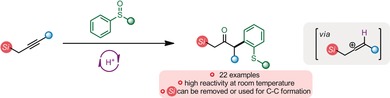

Redox‐neutral strategies for the α‐functionalization of carbonyl compounds through sigmatropic rearrangements have become a powerful tool for the formation of C−C bonds. Recently, our group used this approach for the synthesis of enantioenriched 1,4‐dicarbonyl arrays from vinyl sulfoxides and ynamides (Scheme 1 a).^1^ Similarly, a formal metal‐free α‐arylation of carbonyl compounds can be achieved by using aryl sulfoxides (Scheme 1 a).^2^ However, those methods mandate the presence of heteroatoms2a–2d or aryl rings2e–2f in the alkyne reactants as a crucial feature for stabilization of the vinyl cation intermediate **A** (Scheme 1 a). Even with aryl‐substituted acetylenes, the method requires somewhat forcing conditions (high temperatures and solvent‐free conditions) and is not efficient for alkyl‐substituted acetylenes. Indeed (Scheme 1 b), while phenylacetylene affords arylated products in excess of 90 % yield, the non‐aromatic counterpart cyclohexylacetylene **B** provides a disappointing 32 % yield of product. In the case of unsymmetrical dialkyl‐substituted internal alkynes, this poor reactivity is compounded by a complete lack of regioselectivity, a consequence of unselective protonation.

**Scheme 1:**
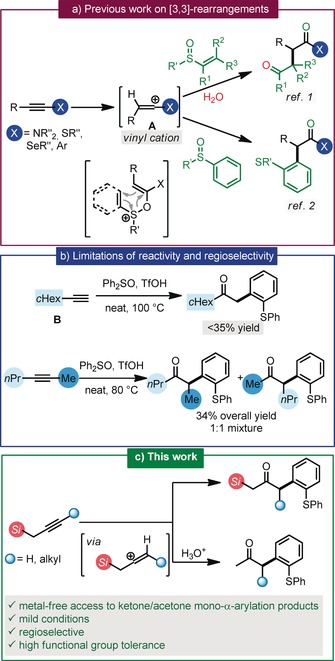
a) Redox‐neutral α‐functionalization of carbonyl compounds, b) limitations of reactivity and selectivity, and c) novel approach presented herein.

In addition, the application of this methodology to the synthesis of acetone‐derived arylation products would require the use of propyne—a gas at ambient pressure—as a starting material, adding yet another layer of complexity to an already convoluted problem. The direct mono‐α‐arylation of simple ketones such as acetone remains a challenge even for palladium‐catalyzed procedures, often requiring the use of acetone as a solvent in order to avoid polyarylation.^3^

To address these issues, we envisioned to use the known ability of silicon to stabilize a β‐positive charge^4^ as a tool to solve the problems of reactivity and selectivity upon formation of vinyl cationic species. Herein, we show how silicon not only activates the alkyne partner but decisively guides regioselectivity for internal, dialkyl‐substituted alkynes while offering a simple protodesilylation path to the formal α‐arylation of acetone (and other simple ketones) (Scheme 1 c).^5^

We first investigated the reaction between terminal propargyl silane **1 a** and different sulfoxides in the presence of a Brønsted acid, exploring a range of solvents (for full optimization details, see the Supporting Information). From the outset, high reactivity at room temperature was observed, in sharp contrast to the sluggishness of aliphatic alkynes described in Scheme 1 b. Pleasingly, the use of bis(trifluoromethane)sulfonimide as acid and either dichloromethane or nitromethane allowed the productive union of **1 a** and diphenyl sulfoxide to deliver the corresponding α‐silyl‐α′‐arylketone **2 a** in a high 84 % isolated yield, along with small amounts (<5 %) of protodesilylated product. The reaction could be readily scaled up to a 10 mmol scale to afford 2.5 g of α‐silyl ketone. A screening of sulfoxides, depicted in Scheme 2, revealed that a diversity of substituents were tolerated at the *para*‐position, such as the electron‐donating methyl, methoxy, and phenyl groups (**2 b**–**d**), halogens (**2 e**–**g**), and an electron‐withdrawing nitrile moiety (**2 h**). A tricyclic sulfoxide derived from dibenzothiophene reacted promptly to afford compound **2 i**. When bis(*meta*,*para*‐dimethylphenyl) sulfoxide was used, the corresponding compound **2 j** was isolated with a high yield but as a 1:1 mixture of regioisomers. Aryl alkyl sulfoxides were also suitable partners in the reaction, as shown by the formation of compounds **2 k**–**o**. Importantly, and as hinted by the occasional observation of minute amounts of protodesilylated material, a simple HCl quench allowed direct access to product **3 a**—the elusive product of redox arylation of propyne and corresponding to a formal metal‐free α‐arylation of acetone (Scheme 2, bottom).

**Scheme 2:**
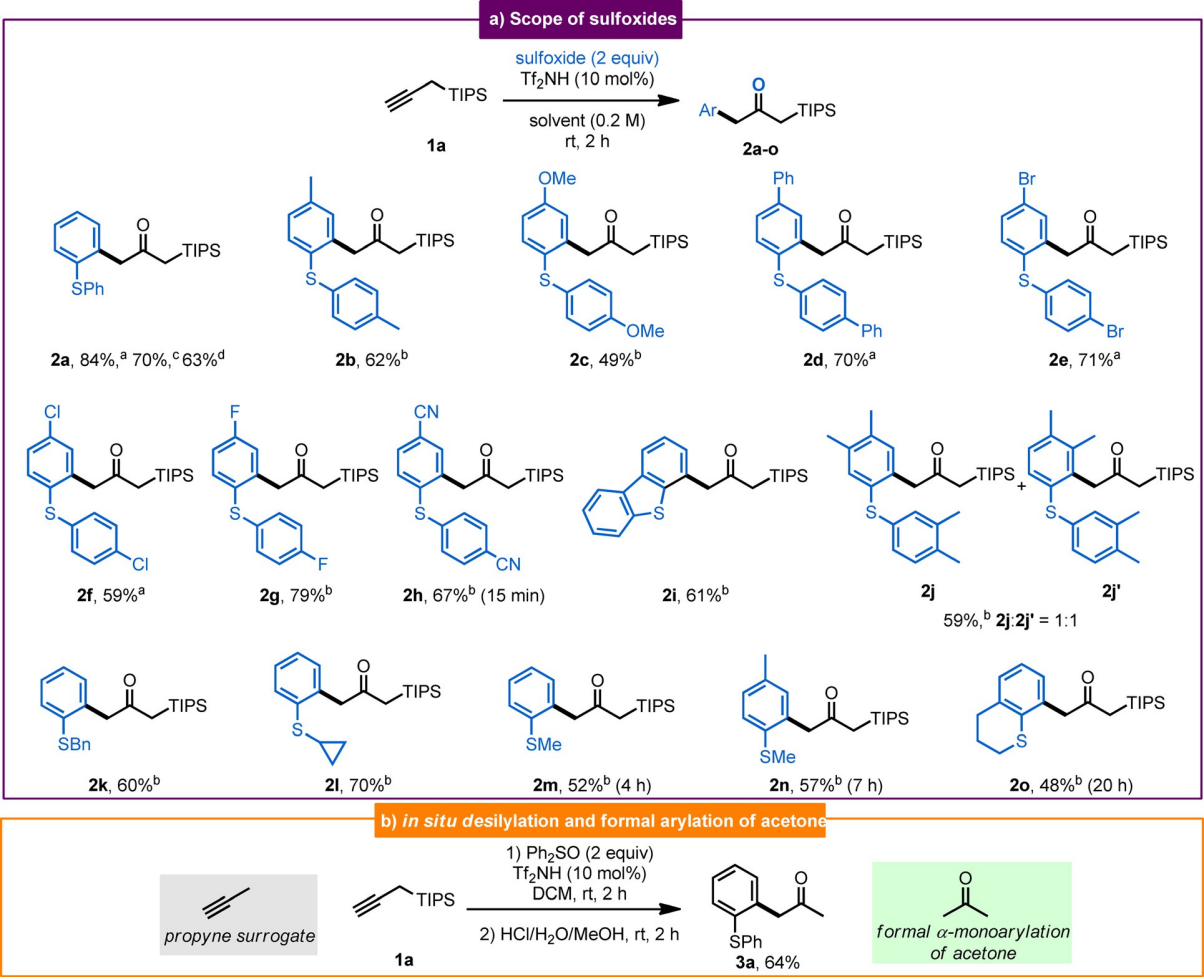
a) Scope of sulfoxide partners and b) in situ desilylation. [a] Reaction was performed in dichloromethane. [b] Reaction was performed in nitromethane. [c] Reaction was performed on 5 mmol scale. [d] Reaction was performed on 10 mmol scale.

Eager to obtain more mechanistic insights, we performed some competition experiments. Using a 1:1 mixture of *p*‐chloro‐ and *p*‐methyl‐arylated sulfoxides, a strong (77:23) preference for the most electron‐rich sulfoxide was observed (Scheme 3 a), akin to the intramolecular case where a sulfoxide carrying both groups was used (Scheme 3 b). These results are consistent with the selectivity previously observed for this type of rearrangement.2e

**Scheme 3:**
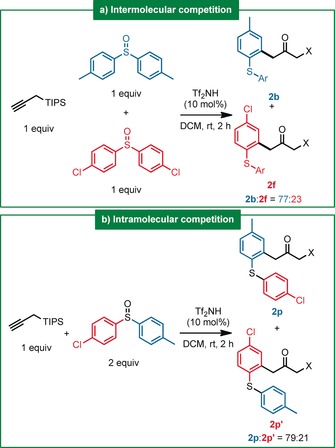
Competition experiments. X=TIPS or H.

Unexpectedly, we observed a marked influence of the substitution pattern of the sulfoxide on the kinetics of the reaction. As shown in Figure 1, when an electron‐rich sulfoxide such as bis(*p*‐methoxyphenyl) sulfoxide was used, the reaction was much slower than in the case of an electron‐poor counterpart (such as for example, the *p*‐fluoro derivative). We measured the initial rate of the reaction for several substituents and a Hammett plot showed a good correlation. These observations suggest that the Brønsted basicity of the sulfoxide plays an important role in the reaction (Figure 1). Therefore, it is reasonable to suggest that triflimide first protonates the sulfoxide, which in turn protonates the propargyl silane with a rate that depends on its substitution.

**Figure 1:**
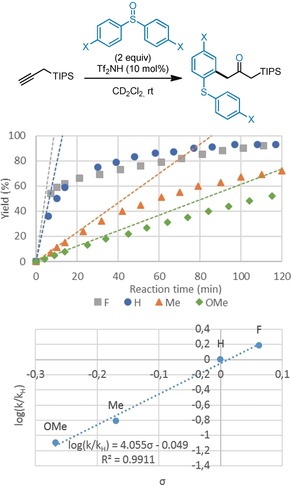
Kinetic experiments (top: kinetic plot; bottom: Hammett plot; for details, see the Supporting Information).

We then turned our attention to the use of internal alkynes in the reaction (Scheme 4). Although the reactions were slower than in the unsubstituted case, the use of a propargyl silane derived from 2‐hexyne led to the corresponding branched product **4 a** with complete regioselectivity. The reaction also proceeded smoothly in the presence of a secondary alkyl substituent (**4 b**,**c**). Acetoxy‐ (**4 d**), bromide‐ (**4 e**), and phthalimide‐ (**4 f**) containing substrates are tolerated (Scheme 4). Propargyl silanes bearing substituents α‐ to the silicon rearranged into 1,3‐dienes under the reaction conditions.^6^

**Scheme 4:**
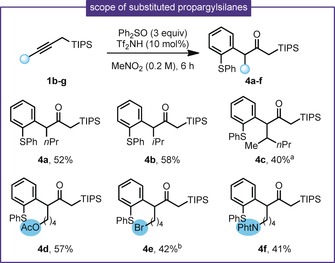
Scope of substituted propargylsilane partners. [a] d.r.=1:1; [b] 8 h reaction time.

Finally, having two methylene groups adjacent to the carbonyl group in **2 a**, we set out to direct substitution at either side of the molecule with by using different sets of reagents. We hypothesized that the obtained α‐silyl‐α′‐arylketones might be amenable nucleophiles for a Mukaiyama aldol‐type coupling.^7^, ^8^ Among other strategies, fluoride sources have been reported to activate the silyl group and generate a nucleophilic enolate.^9^ In our case, the use of tetramethylammonium fluoride triggered an aldol reaction in the presence of benzaldehyde, leading to product **5** in 46 % yield (Scheme 5). We also managed to alkylate the benzylic position using NaHMDS as a base and MeI as an electrophile to give **6**. Finally, the arylsulfanyl moiety can be easily removed under mild conditions while preserving the triisopropylsilyl group to give **7**.2a

**Scheme 5:**
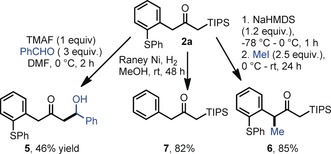
Mukaiyama‐type reaction of **2 a**. TMAF=tetramethylammonium fluoride.

In summary, we have shown that the ability of silicon to stabilize vinyl cationic species leads to a redox arylation whereby the limitations of reactivity and regioselectivity of alkyl‐substituted alkynes are lifted. A range of α‐silyl‐α′‐arylketones were obtained under mild conditions in good to excellent yields and high functional group tolerance. In situ protodesilylation affords the products of a formal acetone monoarylation while Mukaiyama‐type aldol reaction and alkylation of the benzylic position showcase the utility of silicon incorporation in the adducts. Kinetic analysis suggests that an unusual sulfoxide‐mediated protonation is operative.

## Conflict of interest

The authors declare no conflict of interest.

## Supporting information

As a service to our authors and readers, this journal provides supporting information supplied by the authors. Such materials are peer reviewed and may be re‐organized for online delivery, but are not copy‐edited or typeset. Technical support issues arising from supporting information (other than missing files) should be addressed to the authors.

Supplementary
