# Supplementary material for: Vinyl Cation Stabilization by Silicon Enables a Formal Metal‐Free α‐Arylation of Alkyl Ketones
Source: Angew Chem Int Ed Engl. 2019 Oct 22;58(48):17303–6. doi: 10.1002/anie.201909381 (PMC6899746; doi:10.1002/anie.201909381)
Supplement: Supplementary file 1 — Supplementary [file ANIE-58-17303-s001.pdf]

Angewandte Chemie
Eine Zeitschrift der Gesellschaft Deutscher Chemiker

Supporting Information

Vinyl Cation Stabilization by Silicon Enables a Formal Metal-Free α-Arylation of Alkyl Ketones

*Amandine Pons[+], Jean Michalland[+], Wojciech Zawodny[+], Yong Chen, Veronica Tona, and Nuno Maulide**

anie_201909381_sm_miscellaneous_information.pdf

Table of Contents

Experimental Procedures 2
1) Synthesis of starting materials 2
a. Sulfoxides 2
b. Propargyl silanes 4
2) Reaction between propargyl silanes and sulfoxides 7
a. Terminal propargyl silanes 7
b. In situ protodesilylation 12
c. α-branched propargyl silanes 12
d. Internal propargyl silanes 12
e. Competition experiments 15
3) Post-functionalization 17
NMR spectra 19
References 55

Experimental Procedures

General experimental: Unless otherwise stated, all glassware was flame-dried before use and all reactions were performed under an atmosphere of argon. All solvents were distilled from appropriate drying agents prior to use or directly taken from commercial sealed bottles under an atmosphere of argon. All reagents were used as received from commercial suppliers unless otherwise stated. Reaction progress was monitored by thin layer chromatography (TLC) performed on aluminum plates coated with silica gel F254 with 0.2 mm thickness. Chromatograms were visualized by fluorescence quenching with UV light at 254 nm or by staining using potassium permanganate. Flash column chromatography was performed using silica gel 60 (230-400 mesh, Merck and co.). Neat infra-red spectra were recorded using a Brucker Vertex 70 FT-IR spectrometer. Wavenumbers are reported in cm^{-1}. Mass spectra were obtained using a Brucker maXis UHR-TOF spectrometer, using electrospray ionization (ESI) and by Agilent 7200B GC/Q-TOF spectrometer, using electron impact (EI). All ^{1}H NMR and ^{13}C NMR spectra were recorded using a Bruker AV NEO 500, AV III 600 or AV III HD 700 spectrometer in $CDCl_3$. Chemical shifts were given in parts per million (ppm, δ), referenced to the solvent peak of $CDCl_3$, defined at δ = 7.26 ppm (^{1}H NMR) and δ = 77.16 (^{13}C NMR). Coupling constants are quoted in Hz (*J*). ^{1}H NMR splitting patterns were designated as singlet (s), doublet (d), triplet (t), quartet (q), pentet (p). Splitting patterns that could not be interpreted or easily visualized were designated as multiplet (m) or broad (br).

1) Synthesis of starting materials

a. Sulfoxides

4,4'-Sulfinylbis(methoxybenzene)

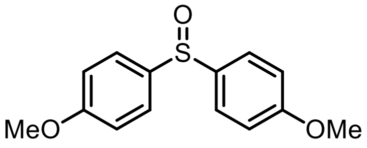

4,4'-Sulfinylbis(methoxybenzene) was prepared according to a literature procedure[1] on a 5 mmol scale and obtained as a white solid (1.08 g, 82% yield). The spectral properties are in agreement with those reported in the literature.

4,4"-Sulfinyldi-1,1'-biphenyl

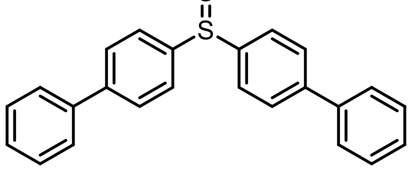

4,4"-Sulfinyldi-1,1'-biphenyl was prepared according to a literature procedure[2] on a 6 mmol scale and the title compound was obtained as a white solid (1.48 g, 70% yield). The spectral properties are in agreement with those reported in the literature.

4,4'-Sulfinylbis(bromobenzene)

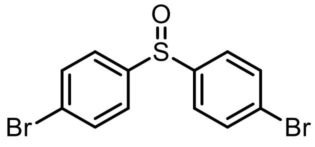

4,4'-Sulfinylbis(bromobenzene) was prepared according to a literature procedure[1] on a 10 mmol scale and was obtained (1.82 g, 50 % yield) as a white solid. The spectral properties are in agreement with those reported in the literature.

4,4'-Sulfinylbis(fluorobenzene)

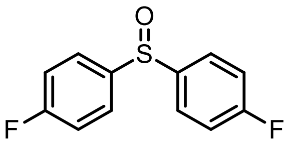

4,4'-Sulfinylbis(fluorobenzene) was prepared according to a literature procedure[1] on a 10 mmol scale and was obtained (1.29 g, 54% yield) as a yellow oil. The spectral properties are in agreement with those reported in the literature.

4,4'-Sulfinyldibenzonitrile

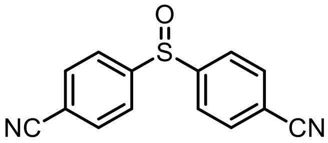

4,4'-Sulfinyldibenzonitrile was prepared according to a literature procedure[1] on a 3.75 mmol scale and was obtained (460 mg, 42% yield over two steps) as a white solid. The spectral properties are in agreement with those reported in the literature.

Dibenzo[b,d]thiophene 5-oxide

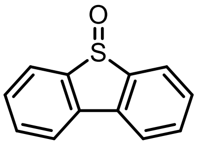

Dibenzo[b,d]thiophene 5-oxide was prepared according to a literature procedure[2] and obtained as a white solid (1.13 g, 56% yield) The spectral properties are in agreement with those reported in the literature.

4,4'-Sulfinylbis(1,2-dimethylbenzene)

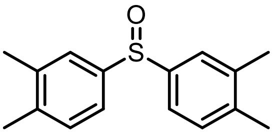

4,4'-Sulfinylbis(1,2-dimethylbenzene) was prepared according to a literature procedure[2] on a 10 mmol scale and obtained as a white solid (2.57 g, 99% yield). The spectral properties are in agreement with those reported in the literature.

1-Methyl-4-(methylsulfinyl)benzene

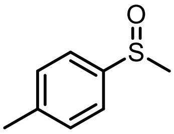

1-Methyl-4-(methylsulfinyl)benzene was prepared according to a literature procedure[3] on a 5.8 mmol scale and obtained as a colourless oil (0.80 g, 90% yield). The spectral properties are in agreement with those reported in the literature.[4]

(Benzylsulfinyl)benzene

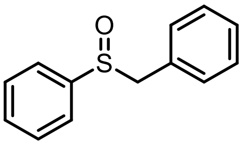

(Benzylsulfinyl)benzene was prepared according to a modified literature procedure.[5] A round-bottom-flask was charged with benzylphenylsulfide (3.0 mmol, 1.0 equiv.) and DCM (0,5 M). After cooling to 0 °C, m-CPBA (3.0 mmol, 1.0 equiv.) was added in portions and the reaction was stirred for 1 h. Aqueous 1 M NaOH (10 mL) was added, the layers were separated and the aqueous phase was extracted with DCM (2x 10 mL). The combined organic extracts were dried over anhydrous $MgSO_4$ and concentrated under reduced pressure. The crude residue was purified by column chromatography (10-20% EtOAc in heptane). The title compound was obtained as a white solid (0.44 g, 68% yield). The spectral properties are in agreement with those reported in the literature.[6]

(Cyclopropylsulfinyl)benzene

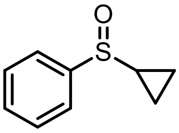

(Cyclopropylsulfinyl)benzene was prepared following a similar procedure used for the synthesis of (benzylsulfinyl)benzene on a 3 mmol scale. The title compound was obtained as a colourless oil (0.42 g, 84% yield). The spectral properties are in agreement with those reported in the literature.[7]

Thiochromane 1-oxide

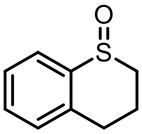

Thiochromane 1-oxide was prepared from thiochromane following a similar procedure used for the synthesis of (benzylsulfinyl)benzene on a 3.3 mmol scale. The title compound was obtained as a colourless oil (0.48 g, 88% yield). The spectral properties are in agreement with those reported in the literature.[9]

1-Chloro-4-(p-tolylsulfinyl)benzene

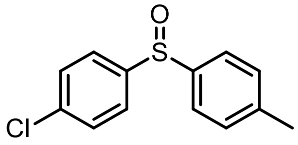

1-Chloro-4-(p-tolylsulfinyl)benzene was prepared according to a literature procedure[2] on a 16 mmol scale and obtained as a white solid (1.19 g, 34% yield over two steps). The spectral properties are in agreement with those reported in the literature.

b. Propargyl silanes

Triisopropyl(3-(trimethylsilyl)prop-2-yn-1-yl)silane (**1a'**)

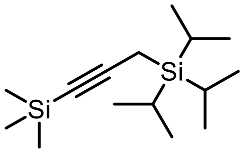

A 1.7 M solution of *t*-BuLi (11.5 mL, 19.6 mmol, 0.98 equiv) was added dropwise to a solution of 1-(trimethylsilyl)-1-propyne (3.1 mL, 20 mmol, 1 equiv) in Et_2O (30 mL) at -60 °C. The mixture was allowed to warm to -10 °C and TIPSCl (4.9 mL, 23 mmol, 1.15 equiv) was added. The mixture was stirred overnight at room temperature, quenched with saturated aqueous NH_4Cl and extracted twice with Et_2O. The combined organic layers were dried over $MgSO_4$ and concentrated under reduced pressure. The crude material was purified by distillation (10 mbar, 100 °C) to afford 4.63 g (83% yield) of **1a'**.
^{1}H NMR (600 MHz, $CDCl_3$): δ 1.60 (s, 2H), 1.18 – 1.12 (m, 3H), 1.08 (d, *J* = 6.9 Hz, 18H), 0.10 (s, 9H) ppm.
^{13}C NMR (150 MHz, $CDCl_3$): δ 106.7, 83.4, 18.7 (6C), 11.2 (3C), 0.8, 0.3 (3C) ppm.
HRMS (EI+): calculated for [M-*i*Pr]$^+$ $C_{12}H_{25}Si_2^+$: 225.1489, found: 225.1489.
IR (cm^{-1}): 3317, 2942, 2866, 2116, 1464, 1385, 1249, 1152, 1067, 1016, 999, 882, 735, 709, 653, 628, 600.

Triisopropyl(prop-2-yn-1-yl)silane (**1a**)

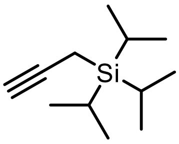

Silver triflate (478 mg, 1.86 mmol, 0.2 equiv) was added to a solution of **1a'** (2.50 g, 9.31 mmol, 1 equiv) in DCM (10 mL), MeOH (6 mL) and water (1.2 mL) at room temperature. The mixture was stirred overnight at room temperature and quenched with saturated aqueous NH_4Cl. The aqueous layer was extracted three times with DCM, the combined organic layers were dried over $MgSO_4$ and concentrated under reduced pressure. The crude material was purified by distillation (20 mbar, 110 °C) to afford 1.40 g (77% yield) of propargyl silane **1a**.
^{1}H NMR (600 MHz, $CDCl_3$): δ 1.79 (t, *J* = 3.0 Hz, 1H), 1.53 (d, *J* = 3.0 Hz, 2H), 1.20 – 1.14 (m, 3H), 1.09 (d, *J* = 7.0 Hz, 18H) ppm.
^{13}C NMR (150 MHz, $CDCl_3$): δ 83.4, 67.1, 18.7 (6C), 11.1 (3C), -1.1 ppm.
HRMS (EI+): calculated for [M-*i*Pr]$^+$ $C_9H_{17}Si^+$: 153,1094 found: 153.1090.
IR (cm^{-1}): 3317, 2942, 2891, 2866, 2816, 1463, 1385, 1249, 1152, 1067, 999, 882, 753, 709, 628, 600.

General procedure A: A 1.7 M solution of *t*BuLi (1.1 equiv) was added to a solution of methyl internal alkyne (1 equiv) in THF (0.1 M) at -78 °C. The mixture was stirred for 10 min at 0 °C, then TIPSCl (1.2 equiv) was added at -78 °C. The mixture was stirred overnight at room temperature and quenched with saturated aqueous NH_4Cl. The aqueous layer was extracted 3 times with Et_2O, the combined organic layers were dried over $MgSO_4$ and concentrated under reduced pressure. The crude material was purified by silica gel column chromatography (typical eluent heptane:EtOAc 100:0 up to heptane:EtOAc 98:2).

Hex-2-yn-1-yltriisopropylsilane (**1b**)

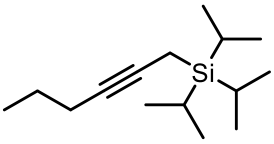

Prepared according to procedure A (34% yield).
^{1}H NMR (600 MHz, $CDCl_3$): δ 2.08 (tt, *J* = 7.0, 2.6 Hz, 2H), 1.51 – 1.42 (m, 4H), 1.17 – 1.11 (m, 3H), 1.08 (d, *J* = 6.6 Hz, 18H), 0.95 (t, *J* = 7.4 Hz, 3H) ppm.
^{13}C NMR (150 MHz, $CDCl_3$): δ 78.8, 78.0, 22.8, 21.2, 18.7 (6C), 13.7, 11.2 (3C), -1.1 ppm.
HRMS (EI+): calculated for [M-*i*Pr]$^+$ $C_{12}H_{23}Si^+$: 195.1564, found: 195.1564.
IR (cm^{-1}): 2959, 2866, 1462, 1382, 1172, 998, 882, 734, 697, 650.

Triisopropyl(4-methylpent-2-yn-1-yl)silane (**1c**)

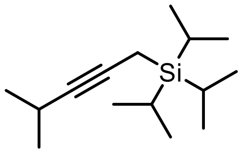

Prepared according to procedure A (25% yield).
^{1}H NMR (600 MHz, $CDCl_3$): δ 2.52 – 2.43 (m, 1H), 1.47 (d, *J* = 2.4 Hz, 2H), 1.16 – 1.11 (m, 3H), 1.10 (d, *J* = 6.8 Hz, 6H), 1.07 (d, *J* = 6.5 Hz, 18H) ppm.
^{13}C NMR (150 MHz, $CDCl_3$): δ 84.7, 77.3, 23.6 (2C), 21.0, 18.7 (6C), 11.2 (3C), -1.1 ppm.
HRMS (EI+): calculated for $[M\text{-}iPr]^+$ $C_{12}H_{23}Si^+$: 195.1564, found: 195.1560.
IR (cm^{-1}): 2940, 2866, 1462, 1319, 1154, 1046, 998, 882, 734, 650.

Triisopropyl(4-methylhept-2-yn-1-yl)silane (**1d**)

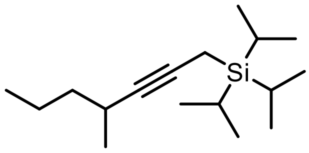

Prepared according to procedure A (27% yield).
^{1}H NMR (600 MHz, $CDCl_3$): δ 2.40 – 2.31 (m, 1H), 1.49 (d, *J* = 2.4 Hz, 2H), 1.39 – 1.30 (m, 3H), 1.15 – 1.06 (m, 25H), 0.89 (t, *J* = 7.2 Hz, 3H) ppm.
^{13}C NMR (150 MHz, $CDCl_3$): 83.6, 78.0, 39.8, 26.1, 21.7, 20.7, 18.8 (6C), 14.1, 11.2 (3C), -1.1 ppm.
HRMS (EI+): calculated for $[M\text{-}iPr]^+$ $C_{14}H_{27}Si^+$: 223.1877, found: 223.1872.
IR (cm^{-1}): 2928, 2865, 1894, 1462, 1381, 1152, 998, 882, 734, 651.

7-(Triisopropylsilyl)hept-5-yn-1-ol (**1e'**)

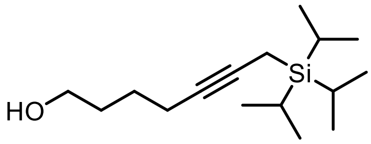

A 2.5 M solution of *n*-BuLi (3.3 mL, 8.1 mmol, 1.05 equiv) was added to a solution of 5-heptynol (870 mg, 7.8 mmol, 1 equiv) in THF (20 mL) at -78 °C. The mixture was stirred for 10 min at room temperature, then cooled again to -78 °C. A 1.7 M solution of *t*-BuLi (4.8 mL, 8.1 mmol, 1.05 equiv) was then added. The mixture was stirred for 10 min at room temperature, then cooled again to -78 °C. TIPSCl (1.8 mL, 1.1 equiv, 8.5 mmol) was added. The mixture was stirred for 2 hours at room temperature and quenched with saturated aqueous NH_4Cl. The aqueous layer was extracted 3 times with Et_2O, the combined organic layers were dried over $MgSO_4$ and concentrated under reduced pressure. The crude product was purified by silica gel column chromatography (heptane/EtOAc 10:0 to 8:2) to afford 760 mg (37% yield) of propargyl silane **1e'**.
^{1}H NMR (600 MHz, $CDCl_3$): δ 3.64 (t, *J* = 6.5 Hz, 2H), 2.15 (tt, *J* = 6.9, 2.6 Hz, 2H), 1.68 – 1.62 (m, 2H), 1.56 – 1.50 (m, 2H), 1.47 (t, *J* = 2.7 Hz, 2H), 1.15 – 1.09 (m, 3H), 1.07 (d, *J* = 6.6 Hz, 18H) ppm.
^{13}C NMR (150 MHz, $CDCl_3$): δ 78.4, 78.4, 62.7, 32.1, 25.6, 18.9, 18.7 (6C), 11.1 (3C), -1.1 ppm.
HRMS (ESI+): calculated for $[M+Na]^+$ $C_{16}H_{32}NaOSi^+$: 291.2115, found: 291.2115.
IR (cm^{-1}): 2959, 2866, 1712, 1679, 1462, 1070, 1013, 998, 882, 734, 650.

7-(Triisopropylsilyl)hept-5-yn-1-yl acetate (**1^e**)

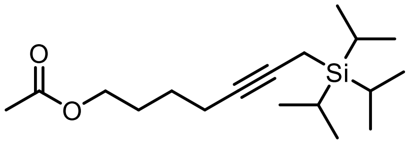

Triethylamine (0.19 mL, 1.4 mmol, 2 equiv) was added to a solution of alcohol **1e'** (187 mg, 0.70 mmol, 1 equiv) and acetyl chloride (0.07 mL, 1.0 mmol, 1.5 equiv) in DCM (5 mL) at room temperature. The mixture was stirred at room temperature overnight, then diluted with water. The aqueous layer was extracted 3 times with DCM, the combined organic layers were dried over $MgSO_4$ and concentrated under reduced pressure. The crude material was purified by silica gel column chromatography (heptane/EtOAc 98:2) to afford 178 mg (82% yield) of product **1e**.
^{1}H NMR (600 MHz, $CDCl_3$): δ 4.06 (t, *J* = 6.6 Hz, 2H), 2.18 – 2.12 (m, 2H), 2.03 (s, 3H), 1.74 – 1.68 (m, 2H), 1.53 – 1.48 (m, 2H), 1.47 (t, *J* = 2.6 Hz, 2H), 1.16 – 1.09 (m, 3H), 1.06 (d, *J* = 6.7 Hz, 18H) ppm.
^{13}C NMR (150 MHz, $CDCl_3$): δ 171.3, 78.6, 78.0, 64.3, 27.9, 25.8, 21.1, 18.8, 18.7 (6C), 11.1 (3C), -1.1 ppm.
HRMS (ESI+): calculated for $[M+Na]^+$ $C_{18}H_{34}NaO_2Si^+$: 333.2220, found: 333.2220.
IR (cm^{-1}): 2940, 2866, 1740, 1462, 1367, 1232, 1039, 882, 734, 650.

(7-bromohept-2-yn-1-yl)triisopropylsilane (**1f**)

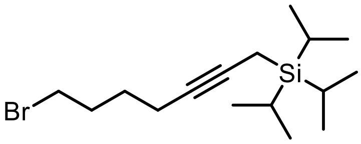

Triphenylphosphine (633 mg, 2.4 mmol, 1.5 equiv) was added to a solution of alcohol **1e'** (432 mg, 1.6 mmol, 1 equiv) and CBr_4 (640 mg, 1.9 mmol, 1.2 equiv) in DCM (3 mL) at 0 °C. The mixture was stirred 15 min at 0 °C then 2 hours at room temperature and concentrated under reduced pressure. Et_2O was added, the mixture was filtered and the filtrate was concentrated under reduced pressure. The crude product was purified by silica gel column chromatography (heptane/EtOAc 100:1 to 98:2) to afford 440 mg (83% yield) of bromide **1f**.

^{1}H NMR (600 MHz, $CDCl_3$): δ 3.41 (t, *J* = 6.8 Hz, 2H), 2.19 – 2.13 (m, 2H), 1.96 (dt, *J* = 14.2, 7.0 Hz, 2H), 1.59 (dt, *J* = 14.3, 7.0 Hz, 2H), 1.47 (t, *J* = 2.7 Hz, 2H), 1.16 – 1.09 (m, 3H), 1.07 (d, *J* = 6.5 Hz, 18H) ppm.

^{13}C NMR (150 MHz, $CDCl_3$): δ 78.9, 77.8, 33.5, 31.9, 27.7, 18.7 (6C), 18.3, 11.1 (3C), -1.1 ppm.

HRMS (EI+): calculated for $[M\text{-}i\text{Pr}]^+$ $C_{13}H_{24}BrSi^+$: 287.0825, found: 287.0823.

IR (cm^{-1}): 2959, 2866, 1462, 1250, 1172, 1016, 998, 882, 734, 697, 650.

2-(7-(Triisopropylsilyl)hept-5-yn-1-yl)isoindoline-1,3-dione (**1g**)

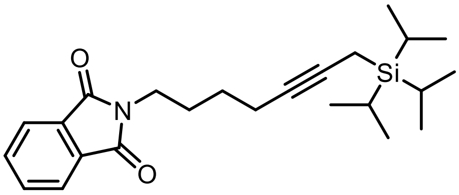

Potassium phthalimide (251 mg, 1.36 mmol, 1.5 equiv) was added to a solution of bromide **1f** (300 mg, 0.91 mmol, 1 equiv) in DMF (3 mL) at room temperature. The mixture was stirred overnight at room temperature, then diluted with water. The aqueous layer was extracted 3 times with EtOAc, the combined organic layers were dried over $MgSO_4$ and concentrated under reduced pressure. The crude product was purified by silica gel column chromatography (heptane/EtOAc 9:1 to 8:2) to afford 370 mg (quantitative yield) of compound **1g**.

^{1}H NMR (600 MHz, $CDCl_3$): δ 7.86 – 7.79 (m, 2H), 7.73 – 7.67 (m, 2H), 3.68 (t, *J* = 7.1 Hz, 2H), 2.19 – 2.13 (m, 2H), 1.76 (dd, *J* = 15.2, 7.1 Hz, 2H), 1.49 (dt, *J* = 14.8, 7.2 Hz, 2H), 1.45 (bs, 2H), 1.14 – 1.06 (m, 3H), 1.04 (d, *J* = 6.7 Hz, 18H) ppm.

^{13}C NMR (150 MHz, $CDCl_3$): δ 168.5 (2C), 134.0 (2C), 132.3 (2C), 123.3 (2C), 78.6, 78.0, 37.7, 28.1, 26.6, 18.8, 18.7 (6C), 11.1 (3C), -1.1 ppm.

HRMS (ESI+): calculated for $[M+Na]^+$ $C_{24}H_{35}NNaO_2Si^+$: 420.2329, found: 420.2323.

IR (cm^{-1}): 2959, 2866, 1772, 1707, 1401, 1367, 1036, 882, 717.

2) Reaction between propargyl silanes and sulfoxides

a. Terminal propargyl silanes

Table S1. Optimization for terminal propargyl silanes.

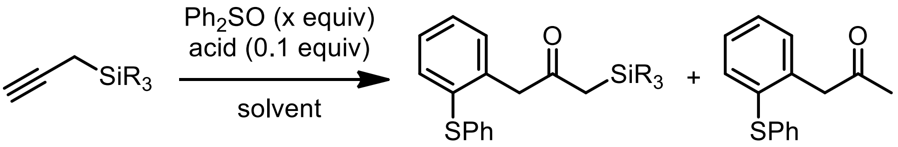

2 3

entry	Si group	acid	temperature – time	concentration	x equiv Ph_2SO	solvent	2	3	starting material
1	TBS	TfOH	0 °C – 5h	0.4 M	3	DCM	9%	4%	29%
2	TIPS	TfOH	rt – 2h	0.4 M	3	DCM	65%	14%	10%
3	TIPS	Tf_2NH	rt – 2h	0.4 M	3	DCM	74%	17%	3%
4	TIPS	Tf_2NH	0 °C – 2h	0.4 M	3	DCM	16%	traces	75%
5	TIPS	Tf_2NH	rt – 2h	0.2 M	3	DCM	77%	16%	2%
**6**	**TIPS**	**Tf_2NH**	**rt – 2h**	**0.2 M**	**2**	**DCM**	**81%**	**10%**	**7%**
7	TIPS	Tf_2NH	rt – 2h	0.2 M	2	$MeNO_2$	70%	19%	0%

General procedure B: A solution of Tf_2NH (5.6 mg, 0.02 mmol, 0.1 equiv) in $MeNO_2$ or DCM (0.1 mL) was added to a solution of alkyne (39 mg, 0.2 mmol, 1 equiv) and sulfoxide (0.4 mmol, 2 equiv) in $MeNO_2$ or DCM (0.9 mL) at room temperature. The mixture was stirred at room temperature for 2 hours and quenched with saturated aqueous $NaHCO_3$. The aqueous layer was extracted 3 times with DCM, the combined organic layers were dried over $MgSO_4$ and concentrated under reduced pressure. The crude material was purified by silica gel column chromatography (typically a gradient elution heptane-EtOAc from 99:1 to 95:5).

1-(2-(Phenylthio)phenyl)-3-(triisopropylsilyl)propan-2-one (**2a**)

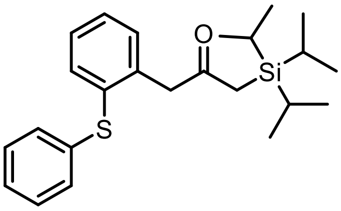

Prepared according to procedure B in DCM (84% yield).
^{1}H NMR (700 MHz, $CDCl_3$): δ 7.40 (dd, J = 7.8, 1.1 Hz, 1H), 7.30 (td, J = 7.5, 1.3 Hz, 1H), 7.27 – 7.20 (m, 4H), 7.18 – 7.12 (m, 3H), 3.97 (s, 2H), 2.25 (s, 2H), 1.17 – 1.10 (m, 3H), 1.06 (d, J = 7.1 Hz, 18H) ppm.
^{13}C NMR (175 MHz, $CDCl_3$): δ 207.0, 137.8, 137.1, 134.7, 134.0, 131.3, 129.2 (2C), 129.0 (2C), 128.6, 128.1, 126.3, 49.9, 29.7, 18.7 (6C), 11.6 (3C) ppm.
HRMS (ESI+): calculated for $[M+H]^+$ $C_{24}H_{35}OSSi^+$: 399.2172, found: 399.2178.
IR (cm^{-1}): 2941, 2890, 2865, 1693, 1582, 1468, 1439, 1404, 1314, 1188, 1044, 1023, 882, 737, 689.

1-(5-Methyl-2-(p-tolylthio)phenyl)-3-(triisopropylsilyl)propan-2-one (**2b**)

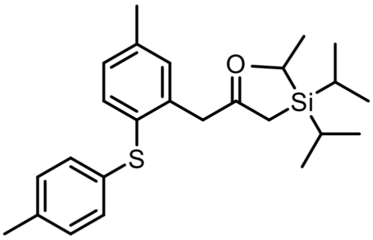

Prepared according to procedure B in $MeNO_2$ (62% yield).

^{1}H NMR (600 MHz, CDCl$_3$): δ 7.29 (d, *J* = 7.9 Hz, 1H), 7.09 – 7.01 (m, 6H), 3.92 (s, 2H), 2.33 (s, 3H), 2.29 (s, 3H), 2.23 (s, 2H), 1.18 – 1.09 (m, 3H), 1.06 (d, *J* = 7.0 Hz, 18H) ppm.
^{13}C NMR (150 MHz, CDCl$_3$): δ 207.5, 138.5, 137.6, 136.1, 134.7, 133.9, 132.0, 130.8, 129.9 (2C), 129.0 (3C), 49.9, 29.7, 21.3, 21.1, 18.7 (6C), 11.6 (3C) ppm.
HRMS (ESI+): calculated for [M+H]$^+$ $C_{26}H_{39}OSSi^+$: 427.2485, found: 427.2488.
IR (cm^{-1}): 2941, 2864, 1692, 1492, 1462, 1231, 1190, 1105, 1043, 1016, 881, 802, 647.

1-(5-Methoxy-2-((4-methoxyphenyl)thio)phenyl)-3-(triisopropylsilyl)propan-2-one (**2c**)

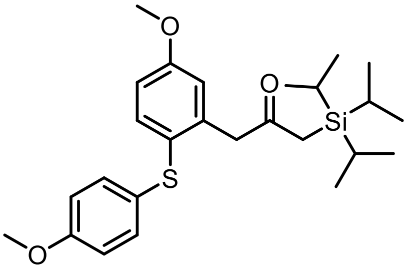

Prepared according to procedure B in $MeNO_2$ (49% yield).
^{1}H NMR (600 MHz, CDCl$_3$): δ 7.34 (d, *J* = 8.5 Hz, 1H), 7.07 (d, *J* = 8.8 Hz, 2H), 6.81 – 6.76 (m, 4H), 3.94 (s, 2H), 3.79 (s, 3H), 3.76 (s, 3H), 2.24 (s, 2H), 1.17 – 1.04 (m, 21H) ppm.
^{13}C NMR (150 MHz, CDCl$_3$): δ 207.1, 159.8, 158.5, 139.3, 136.3, 130.5 (2C), 128.5, 125.6, 116.5, 114.8 (2C), 114.0, 55.5, 55.4, 50.1, 29.7, 18.7 (6C), 11.6 (3C) ppm.
HRMS (ESI+): calculated for [M+H]$^+$ $C_{26}H_{39}O_3SSi^+$: 459.2384, found: 459.2384.
IR (cm^{-1}): 2940, 2866, 1691, 1592, 1566, 1492, 1458, 1284, 1236, 1172, 1028, 882, 819, 642.

1-(4-([1,1'-Biphenyl]-4-ylthio)-[1,1'-biphenyl]-3-yl)-3-(triisopropylsilyl)propan-2-one (**2d**)

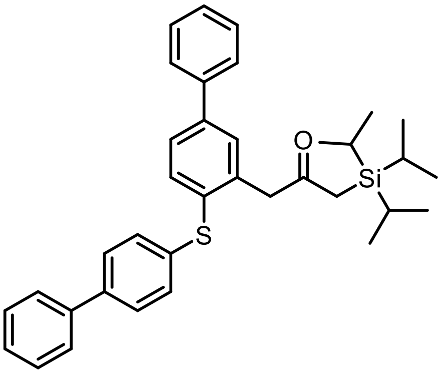

Prepared according to procedure B in DCM (70% yield).
^{1}H NMR (600 MHz, CDCl$_3$): δ 7.62 – 7.58 (m, 2H), 7.58 – 7.54 (m, 2H), 7.54 – 7.46 (m, 5H), 7.46 – 7.40 (m, 4H), 7.38 – 7.32 (m, 2H), 7.29 – 7.24 (m, 2H), 4.05 (s, 2H), 2.30 (s, 2H), 1.18 – 1.13 (m, 3H), 1.09 – 1.06 (m, 18H) ppm.
^{13}C NMR (150 MHz, CDCl$_3$): δ 207.1, 141.5, 140.5, 140.3, 139.4, 138.1, 136.2, 135.1, 132.9, 130.0, 129.4 (2C), 129.0 (2C), 129.0 (2C), 128.0 (2C), 127.8, 127.5, 127.3 (2C), 127.0 (2C), 126.9, 50.1, 29.9, 18.7 (6C), 11.6 (3C) ppm.
HRMS (ESI+): calculated for [M+H]$^+$ $C_{36}H_{43}OSSi^+$: 551.2798, found: 551.2783.
IR (cm^{-1}): 3058, 3028, 2940, 2889, 2864, 1692, 1598, 1468, 1386, 1316, 1149, 1106, 1065, 1040, 1004, 882, 757, 695.

1-(5-Bromo-2-((4-bromophenyl)thio)phenyl)-3-(triisopropylsilyl)propan-2-one (**2e**)

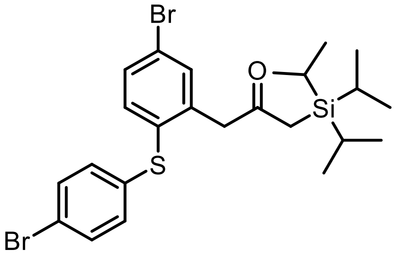

Prepared according to procedure B in DCM (71% yield).
^{1}H NMR (600 MHz, CDCl$_3$): δ 7.39 (d, *J* = 2.2 Hz, 1H), 7.38 – 7.34 (m, 3H), 7.23 (d, *J* = 8.3 Hz, 1H), 7.01 – 6.97 (m, 2H), 3.91 (s, 2H), 2.25 (s, 2H), 1.15 – 1.09 (m, 3H), 1.06 (d, *J* = 6.6 Hz, 18H) ppm.
^{13}C NMR (150 MHz, CDCl$_3$): δ 206.0, 139.8, 136.0, 135.9, 134.4, 132.8, 132.4 (2C), 131.5, 130.6 (2C), 123.0, 120.6, 49.6, 30.0, 18.7 (6C), 11.6 (3C) ppm.
HRMS (ESI+): calculated for [M+H]$^+$ $C_{24}H_{33}Br_2OSSi^+$: 555.0383, found: 555.0382.
IR (cm^{-1}): 2941, 2865, 1694, 1466, 1384, 1360, 1309, 1219, 1199, 1186, 1100, 1083, 1044, 1007, 809, 730, 645.

1-(5-Chloro-2-((4-chlorophenyl)thio)phenyl)-3-(triisopropylsilyl)propan-2-one (**2f**)

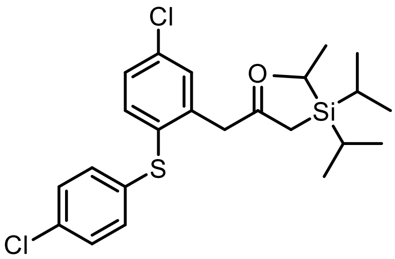

Prepared according to procedure B in DCM (59% yield).
^{1}H NMR (600 MHz, CDCl$_3$): δ 7.31 (d, *J* = 8.3 Hz, 1H), 7.27 – 7.20 (m, 4H), 7.06 (d, *J* = 8.5 Hz, 2H), 3.94 (s, 2H), 2.26 (s, 2H), 1.18 – 1.10 (m, 3H), 1.07 (d, *J* = 6.8 Hz, 18H) ppm.
^{13}C NMR (150 MHz, CDCl$_3$): δ 206.0, 139.5, 135.8, 135.3, 134.8, 132.6, 132.3, 131.5, 130.3 (2C), 129.5 (2C), 128.5, 49.7, 29.9, 18.7 (6C), 11.6 (3C) ppm.
HRMS (ESI+): calculated for $[M+H]^+$ $C_{24}H_{33}Cl_2OSSi^+$: 467.1393, found: 467.1380.
IR (cm^{-1}): 2941, 2864, 2848, 1697, 1472, 1461, 1199, 1186, 1100, 1088, 1009, 882, 817.

1-(5-Fluoro-2-((4-fluorophenyl)thio)phenyl)-3-(triisopropylsilyl)propan-2-one (**2g**)

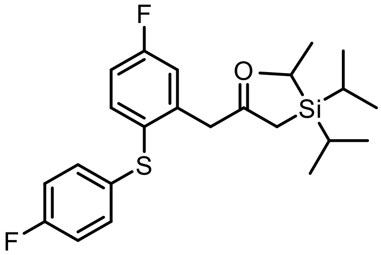

Prepared according to procedure B in $MeNO_2$ (79% yield).
^{1}H NMR (600 MHz, CDCl$_3$): δ 7.37 (dd, *J* = 8.6, 5.7 Hz, 1H), 7.12 – 7.08 (m, 2H), 6.99 (dd, *J* = 9.3, 2.8 Hz, 1H), 6.97 – 6.92 (m, 3H), 3.96 (s, 2H), 2.25 (s, 2H), 1.16 – 1.10 (m, 3H), 1.07 (d, *J* = 6.7 Hz, 18H) ppm.
^{13}C NMR (150 MHz, CDCl$_3$): δ 206.2, 163.1 (d, *J* = 148.2 Hz), 161.5 (d, *J* = 145.6 Hz), 140.2 (d, *J* = 8.2 Hz), 136.6 (d, *J* = 8.4 Hz), 132.2 (d, *J* = 3.1 Hz), 130.8 (d, *J* = 7.9 Hz, 2C), 129.4 (d, *J* = 3.2 Hz), 118.4 (d, *J* = 22.2 Hz), 116.4 (d, *J* = 22.1 Hz, 2C), 115.5 (d, *J* = 21.6 Hz), 49.8, 29.9, 18.7 (6C), 11.6 (3C) ppm.
HRMS (ESI+): calculated for $[M+H]^+$ $C_{24}H_{33}F_2OSSi^+$: 435.1984, found: 435.1978.
IR (cm^{-1}): 2942, 2891, 2865, 1696, 1578, 1489, 1470, 1402, 1271, 1226, 1193, 1155, 1104, 1043, 1013, 880, 821, 759, 641.

4-((4-Cyanophenyl)thio)-3-(2-oxo-3-(triisopropylsilyl)propyl)benzonitrile (**2h**)

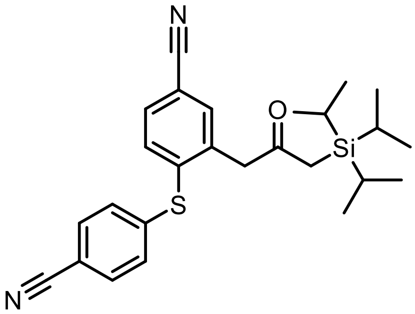

Prepared according to procedure B in $MeNO_2$ with a reaction time of 15 minutes (67% yield).
^{1}H NMR (600 MHz, CDCl$_3$): δ 7.54 (m, 2H), 7.52 (m, 1H), 7.51 (s, 1H), 7.45 (m, 1H), 7.24 – 7.20 (m, 2H), 4.00 (s, 2H), 2.29 (s, 2H), 1.15 – 1.09 (m, 3H), 1.06 (d, *J* = 6.6 Hz, 18H) ppm.
^{13}C NMR (150 MHz, CDCl$_3$): δ 205.1, 142.5, 139.2, 139.1, 134.9, 134.8, 132.9 (2C), 131.6, 129.4 (2C), 118.4, 118.1, 112.8, 110.5, 49.5, 30.1, 18.6 (6C), 11.6 (3C) ppm.
HRMS (ESI+): calculated for $[M+H]^+$ $C_{26}H_{33}N_2OSSi^+$: 449.2077, found: 449.2072.
IR (cm^{-1}): 2941, 2890, 2865, 2228, 1693, 1590, 1484, 1468, 1400, 1314, 1276, 1193, 1105, 1082, 1048, 1016, 882, 823, 754, 689, 674, 646.

1-(Dibenzo[b,d]thiophen-4-yl)-3-(triisopropylsilyl)propan-2-one (**2i**)

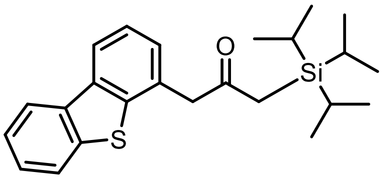

Prepared according to procedure B in $MeNO_2$ (61% yield).
^{1}H NMR (600 MHz, $CDCl_3$): δ 8.18 – 8.12 (m, 1H), 8.08 (d, *J* = 7.8 Hz, 1H), 7.90 – 7.84 (m, 1H), 7.50 – 7.43 (m, 3H), 7.34 (d, *J* = 7.3 Hz, 1H), 4.01 (s, 2H), 2.34 (s, 2H), 1.24 – 1.18 (m, 3H), 1.12 (d, *J* = 7.2 Hz, 18H) ppm.
^{13}C NMR (150 MHz, $CDCl_3$): δ 206.3, 140.1, 139.1, 136.1, 136.1, 129.6, 127.9, 126.9, 125.1, 124.6, 122.9, 121.9, 120.5, 51.2, 29.5, 18.7 (6C), 11.6 (3C) ppm.
HRMS (ESI+): calculated for $[M+H]^+$ $C_{24}H_{33}OSSi^+$: 397.2016, found: 397.2007.
IR (cm^{-1}): 2940, 2888, 2864, 1691, 1462, 1442, 1401, 1308, 1226, 1191, 1107, 1057, 1039, 1018, 881, 747, 660.

1-(2-((3,4-Dimethylphenyl)thio)-4,5-dimethylphenyl)-3-(triisopropylsilyl)propan-2-one (**2j**/**2j’**)

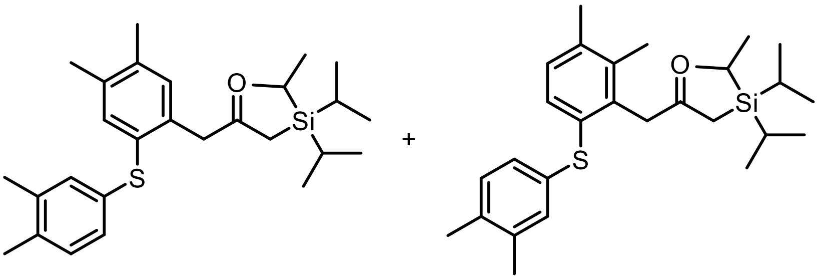

Prepared according to procedure B in $MeNO_2$ with a 2.5 hours reaction time (59% yield, obtained as a 1:1 mixture of inseparable regioisomers, described here as a mixture).
^{1}H NMR (700 MHz, $CDCl_3$): δ 7.21 (s, 1H), 7.17 (d, *J* = 7.9 Hz, 1H), 7.04 – 7.00 (m, 2H), 6.98 (d, *J* = 7.9 Hz, 2H), 6.94 (s, 2H), 6.85 – 6.80 (m, 2H), 4.15 (s, 2H), 3.88 (s, 2H), 2.28 (s, 3H), 2.23 (s, 3H), 2.22 (s, 2H), 2.20 (s, 2H), 2.19 (s, 6H), 2.18 (s, 9H), 2.14 (s, 3H), 1.12 (dt, *J* = 8.6, 7.4 Hz, 6H), 1.07 – 1.04 (m, 36H).
^{13}C NMR (175 MHz, $CDCl_3$): δ 207.8, 207.0, 137.5, 137.4, 137.4, 137.4, 136.6, 136.6, 136.1, 135.3, 134.7, 134.6, 134.4, 134.3, 132.4, 132.0, 131.8, 130.5, 130.4 (3C), 130.2, 129.8, 129.6, 126.6, 126.2, 49.5, 48.0, 29.6, 29.4, 21.1, 19.9, 19.8, 19.7, 19.4 (2C), 19.4, 18.8 (6C), 18.7 (6C), 16.9, 11.7 (3C), 11.6 (3C) ppm.
HRMS (ESI+): calculated for $[M+H]^+$ $C_{28}H_{43}OSSi^+$: 455.2798, found: 455.2798.
IR (cm^{-1}): 2940, 2865, 1693, 1597, 1457, 1383, 1311, 1244, 1187, 1108, 1043, 1017, 997, 882, 809, 745, 689, 651.

1-(2-(Benzylthio)phenyl)-3-(triisopropylsilyl)propan-2-one (**2k**)

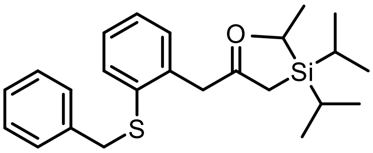

Prepared according to procedure B in $MeNO_2$ (60% yield).
^{1}H NMR (600 MHz, $CDCl_3$): δ 7.40 – 7.36 (m, 1H), 7.28 – 7.22 (m, 3H), 7.22 – 7.17 (m, 4H), 7.16 – 7.11 (m, 1H), 4.01 (s, 2H), 3.84 (s, 2H), 2.27 (s, 2H), 1.20 – 1.13 (m, 3H), 1.09 (d, *J* = 7.1 Hz, 18H) ppm.
^{13}C NMR (150 MHz, $CDCl_3$): δ 207.3, 137.6, 136.9, 135.8, 132.2, 130.7, 129.0 (2C), 128.5 (2C), 127.8, 127.3, 127.3, 50.0, 40.3, 29.6, 18.7 (6C), 11.6 (3C) ppm.
HRMS (ESI+): calculated for $[M+H]^+$ $C_{25}H_{37}OSSi^+$: 413.2329, found: 413.2325.
IR (cm^{-1}): 2940, 2890, 2865, 1691, 1587, 1466, 1404, 1316, 1188, 1104, 1043, 1016, 882, 743, 696, 664.

1-(2-(Cyclopropylthio)phenyl)-3-(triisopropylsilyl)propan-2-one (**2l**)

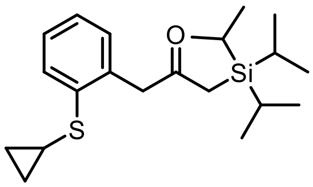

Prepared according to procedure B in $MeNO_2$ (70% yield).
^{1}H NMR (600 MHz, $CDCl_3$): δ 7.61 – 7.58 (m, 1H), 7.26 – 7.23 (m, 1H), 7.14 – 7.09 (m, 2H), 3.81 (s, 2H), 2.28 (s, 2H), 2.14 (tt, *J* = 7.4, 4.4 Hz, 1H), 1.20 – 1.14 (m, 3H), 1.09 (d, *J* = 7.0 Hz, 18H), 1.07 – 1.03 (m, 2H), 0.70 – 0.66 (m, 2H) ppm.
^{13}C NMR (150 MHz, $CDCl_3$): δ 207.1, 138.6, 133.0, 130.5, 127.7, 127.3, 125.4, 49.6, 29.5, 18.7 (6C), 12.6, 11.6 (3C), 8.7 (2C) ppm.
HRMS (ESI+): calculated for $[M+H]^+$ $C_{21}H_{35}OSSi^+$: 363.2172, found: 363.2167.

IR (cm^{-1}): 2941, 2890, 2865, 1692, 1465, 1313, 1230, 1188, 1104, 1042, 881, 741, 663.

1-(2-(Methylthio)phenyl)-3-(triisopropylsilyl)propan-2-one (**2m**)

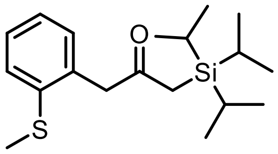

Prepared according to procedure B in $MeNO_2$ with a reaction time of 4 h (52% yield).
^{1}H NMR (600 MHz, $CDCl_3$): δ 7.29 (d, *J* = 7.7 Hz, 1H), 7.27 – 7.22 (m, 1H), 7.14 (s, 1H), 7.13 (s, 1H), 3.90 (s, 2H), 2.44 (s, 3H), 2.31 (s, 2H), 1.21 – 1.15 (m, 3H), 1.10 (d, *J* = 7.1 Hz, 18H) ppm.
^{13}C NMR (150 MHz, $CDCl_3$): δ 207.2, 138.1, 134.2, 130.6, 127.9, 127.3, 125.7, 49.8, 29.6, 18.7 (6C), 16.9, 11.6 (3C) ppm.
HRMS (ESI+): calculated for $[M+H]^+$ $C_{19}H_{33}OSSi^+$: 337.2016, found: 337.2012.
IR (cm^{-1}): 2940, 2865, 1692, 1466, 1439, 1314, 1230, 1187, 1105, 1041, 999, 882, 837, 742, 663.

1-(5-Methyl-2-(methylthio)phenyl)-3-(triisopropylsilyl)propan-2-one (**2n**)

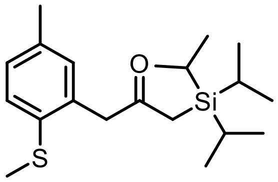

Prepared according to procedure B in $MeNO_2$ with a reaction time of 7 hours (57% yield).
^{1}H NMR (600 MHz, $CDCl_3$): δ 7.22 (d, *J* = 8.0 Hz, 1H), 7.05 (dd, *J* = 8.0, 1.4 Hz, 1H), 6.96 (d, *J* = 1.1 Hz, 1H), 3.89 (s, 2H), 2.41 (s, 3H), 2.31 (s, 2H), 2.29 (s, 3H), 1.20 – 1.16 (m, 3H), 1.10 (d, *J* = 7.0 Hz, 18H) ppm.
^{13}C NMR (150 MHz, $CDCl_3$): δ 207.6, 135.8, 134.6, 134.4, 131.4, 128.8, 128.5, 49.9, 29.6, 21.0, 18.7 (6C), 17.6, 11.6 (3C) ppm.
HRMS (ESI+): calculated for $[M+H]^+$ $C_{20}H_{35}OSSi^+$: 351.2172, found: 351.2161.
IR (cm^{-1}): 2941, 2922, 2890, 2865, 1692, 1463, 1315, 1232, 1191, 1107, 1042, 999, 881, 806, 758, 698, 649.

1-(Thiochroman-8-yl)-3-(triisopropylsilyl)propan-2-one (**2o**)

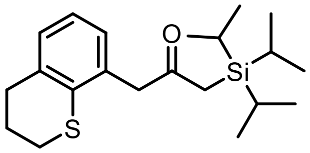

Prepared according to procedure B in $MeNO_2$ with a reaction time of 20 hours (48% yield).
^{1}H NMR (600 MHz, $CDCl_3$): δ 6.98 – 6.93 (m, 3H), 3.78 (s, 2H), 3.03 – 2.98 (m, 2H), 2.85 – 2.80 (m, 2H), 2.31 (s, 2H), 2.12 – 2.04 (m, 2H), 1.21 – 1.14 (m, 3H), 1.09 (d, *J* = 7.0 Hz, 18H) ppm.
^{13}C NMR (150 MHz, $CDCl_3$): δ 207.4, 135.3, 133.3, 132.3, 128.7, 128.6, 123.9, 49.7, 30.5, 29.5, 28.1, 22.8, 18.7 (6C), 11.6 (3C) ppm.
HRMS (ESI+): calculated for $[M+H]^+$ $C_{21}H_{35}OSSi^+$: 363.2172, found: 363.2178.
IR (cm^{-1}): 2940, 2865, 1692, 1466, 1439, 1314, 1230, 1187, 1105, 1041, 999, 882, 837, 742, 663.

b. In situ protodesilylation

1-(2-(phenylthio)phenyl)propan-2-one (**3a**)
A solution of Tf_2NH (5.6 mg, 0.02 mmol, 0.1 equiv) in DCM (0.1 mL) was added to a solution of **1a** (39 mg, 0.2 mmol, 1 equiv) and diphenylsulfoxide (81 mg, 0.4 mmol, 2 equiv) in DCM (0.9 mL) at room temperature. The mixture was stirred at room temperature for 2 hours and concentrated under reduced pressure. MeOH (3 mL) and 1 M aqueous HCl (1 mL) were added, the mixture was stirred at rt for 2 hours then quenched with saturated aqueous $NaHCO_3$. The aqueous layer was extracted 3 times with DCM, the combined organic layers were dried over $MgSO_4$ and concentrated under reduced pressure. The crude material was purified by silica gel column chromatography (64% yield).

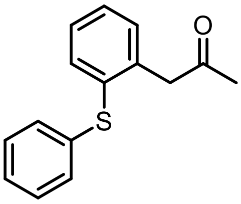

^{1}H NMR (500 MHz, $CDCl_3$): δ 7.44 – 7.37 (m, 1H), 7.33 – 7.29 (m, 1H), 7.28 – 7.26 (m, 2H), 7.26 – 7.23 (m, 2H), 7.21 – 7.15 (m, 3H), 3.91 (s, 2H), 2.14 (s, 3H) ppm.

^{13}C NMR (125 MHz, $CDCl_3$): δ 205.9, 137.3, 136.5, 134.5, 134.3, 131.5, 129.5 (2C), 129.3 (2C), 128.6, 128.4, 126.7, 49.0, 29.9 ppm.

HRMS (ESI+): calculated for $[M+H]^+$ $C_{15}H_{15}OS^+$: 243.0838, found: 243.0840.

IR (cm^{-1}): 3056, 1718, 1580, 1473, 1438, 1354, 1320, 1157, 1059, 1023, 739, 689.

c. α-branched propargyl silanes

When propargyl silanes bearing substituents α- to the silicon were used, the main product of the reaction was a rearranged diene resulting from 1,2-silyl shift. This event has been described in the literature under similar conditions.[10]

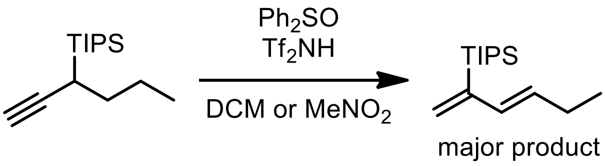

d. Internal propargyl silanes

Table S2. Optimization for internal propargyl silanes.

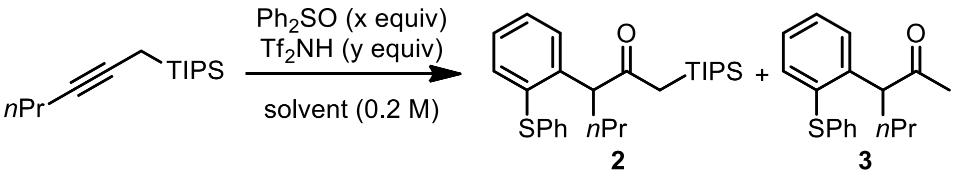

entry	temperature – time	equiv Ph_2SO	solvent	equiv Tf_2NH	2	3	starting material
**1**	rt – 5h	2	$MeNO_2$	0.1	42%	16%	traces
**2**	rt – 5h	3	$MeNO_2$	0.1	53%	12%	18%
**3**	rt – 5h	3	DCM	0.1	21%	6%	35%
**4**	0 °C – 18h	3	$MeNO_2$	0.1	13%	1%	66%
**5**	rt – 2h	3	$MeNO_2$	0.25	42%	33%	traces
**6**	**rt – 6 h**	**3**	**$MeNO_2$**	**0.1**	**59%**	**13%**	**14%**
**7**	rt – 8 h	3	$MeNO_2$	0.1	56%	18%	8%

General procedure C: A solution of Tf_2NH (5.6 mg, 0.02 mmol, 0.1 equiv) in $MeNO_2$ (0.1 mL) was added to a solution of alkyne (0.2 mmol, 1 equiv) and diphenylsulfoxide (121 mg, 0.6 mmol, 3 equiv) in $MeNO_2$ (0.9 mL) at room temperature. The mixture was stirred at room temperature for 6 hours and quenched with saturated aqueous $NaHCO_3$. The aqueous layer was extracted 3 times with DCM, the combined organic layers were dried over $MgSO_4$ and concentrated under reduced pressure. The crude material was purified by silica gel column chromatography (typically elution heptane-EtOAc 100:0 to 95:5).

3-(2-(Phenylthio)phenyl)-1-(triisopropylsilyl)hexan-2-one (**4a**)

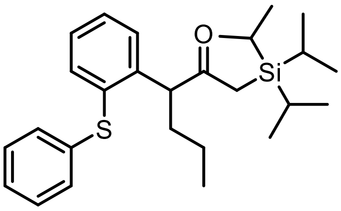

Prepared according to procedure C (52% yield).
^{1}H NMR (600 MHz, CDCl$_3$): δ 7.37 (dd, *J* = 7.8, 1.1 Hz, 1H), 7.28 – 7.16 (m, 8H), 4.55 (t, *J* = 6.9 Hz, 1H), 2.18 (d, *J* = 11.7 Hz, 1H), 1.94 – 1.86 (m, 2H), 1.57 (dddd, *J* = 13.4, 10.3, 8.0, 5.1 Hz, 1H), 1.22 – 1.14 (m, 1H), 1.10 – 0.97 (m, 22H), 0.79 (t, *J* = 7.3 Hz, 3H) ppm.
^{13}C NMR (150 MHz, CDCl$_3$): δ 210.0, 142.1, 136.8, 134.4, 134.4, 129.7 (2C), 129.3 (2C), 128.8, 128.3, 127.8, 126.7, 56.3, 35.2, 29.2, 20.6, 18.7 (3C), 18.7 (3C), 14.3, 11.6 (3C) ppm.
HRMS (ESI+): calculated for $[M+H]^+$ $C_{27}H_{41}OSSi^+$: 441.2642, found: 441.2643.
IR (cm^{-1}): 2955, 2866, 1691, 1466, 1438, 1246, 1197, 1034, 998, 882, 735, 689, 653.

4-Methyl-3-(2-(phenylthio)phenyl)-1-(triisopropylsilyl)pentan-2-one (**4b**)

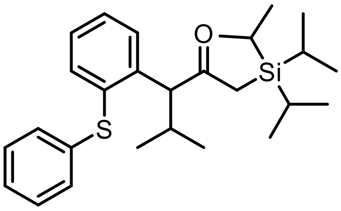

Prepared according to procedure C (58% yield).
^{1}H NMR (700 MHz, CDCl$_3$): δ 7.33 (dd, *J* = 7.8, 0.9 Hz, 1H), 7.29 – 7.23 (m, 6H), 7.23 – 7.19 (m, 1H), 7.17 (ddd, *J* = 7.9, 7.0, 1.9 Hz, 1H), 4.38 (d, *J* = 9.7 Hz, 1H), 2.42 – 2.34 (m, 1H), 2.17 (d, *J* = 12.4 Hz, 1H), 1.89 (dd, *J* = 12.4, 0.9 Hz, 1H), 1.04 – 0.99 (m, 24H), 0.59 (d, *J* = 6.9 Hz, 3H).
^{13}C NMR (175 MHz, CDCl$_3$): δ 209.9, 140.8, 136.6, 135.4, 134.0, 130.1 (2C), 129.4 (2C), 128.4, 128.4, 127.8, 126.8, 63.7, 31.1, 29.7, 22.2, 20.0, 18.9 (3C), 18.7 (3C), 11.7 (3C) ppm.
HRMS (ESI+): calculated for $[M+Na]^+$ $C_{27}H_{40}NaOSSi^+$: 463.2461, found: 463.2460.
IR (cm^{-1}): 2940, 2866, 1683, 1466, 1032, 882, 763, 735, 689.

4-Methyl-3-(2-(phenylthio)phenyl)-1-(triisopropylsilyl)heptan-2-one (**4c**)

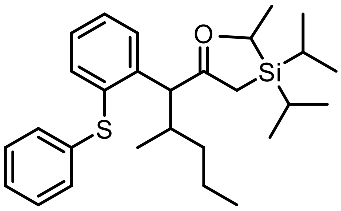

Prepared according to procedure C (40% yield, 1:1 dr).
^{1}H NMR (600 MHz, CDCl$_3$): δ 7.36 – 7.32 (m, 2H), 7.30 – 7.15 (m, 16H), 4.48 (d, *J* = 9.7 Hz, 1H), 4.45 (d, *J* = 10.2 Hz, 1H), 2.33 – 2.24 (m, 2H), 2.21 (d, *J* = 12.4 Hz, 1H), 2.16 (d, *J* = 12.5 Hz, 1H), 1.92 (dd, *J* = 12.6, 0.8 Hz, 1H), 1.91 (dd, *J* = 12.4, 0.9 Hz, 1H), 1.53 (tdd, *J* = 10.4, 5.9, 3.1 Hz, 1H), 1.44 – 1.37 (m, 1H).1.34 – 1.27 (m, 3H), 1.09 – 1.04 (m, 6H), 1.04 – 1.00 (m, 36H), 0.98 (d, *J* = 6.5 Hz, 3H), 0.92 – 0.87 (m, 5H), 0.83 – 0.77 (m, 1H), 0.71 (t, *J* = 7.3 Hz, 3H), 0.57 (d, *J* = 6.9 Hz, 3H) ppm.
^{13}C NMR (150 MHz, CDCl$_3$): δ 210.0 (2C), 140.6, 140.6, 136.7, 136.6, 135.5, 135.5, 134.0, 134.0, 130.1 (2C), 130.1 (2C), 129.4 (4C), 128.7, 128.6, 128.4, 128.3, 127.8, 127.7, 126.8 (2C), 63.1, 62.6, 38.1, 35.5, 35.5, 35.4, 29.8, 29.8, 20.4, 20.0, 18.9 (3C), 18.8 (3C), 18.7 (3C), 18.7 (3C), 18.5, 16.4, 14.4, 14.2, 11.7 (3C), 11.7 (3C) ppm.
HRMS (ESI+): calculated for $[M+Na]^+$ $C_{29}H_{44}NaOSSi^+$: 491.2774, found: 491.2773.
IR (cm^{-1}): 2938, 2865, 2690, 1582, 1465, 1439, 1237, 11971037, 1024, 999, 882, 735, 690, 652.

6-Oxo-5-(2-(phenylthio)phenyl)-7-(triisopropylsilyl)heptyl acetate (**4d**)

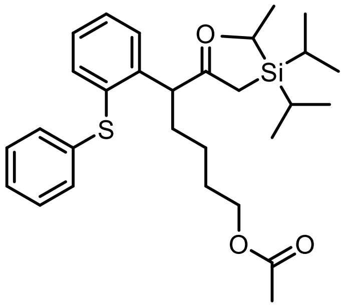

Prepared according to procedure C (57% yield).

^{1}H NMR (700 MHz, CDCl$_3$): δ 7.41 – 7.39 (m, 1H), 7.29 – 7.25 (m, 3H), 7.23 – 7.18 (m, 5H), 4.53 (bs, 1H), 3.91 (td, *J* = 6.8, 3.6 Hz, 2H), 2.17 (d, *J* = 11.8 Hz, 1H), 1.99 (s, 3H), 1.97 – 1.91 (m, 1H), 1.62 – 1.57 (m, 1H), 1.56 – 1.52 (m, 1H), 1.49 – 1.44 (m, 1H), 1.23 – 1.16 (m, 1H), 1.11 – 1.03 (m, 5H), 1.02 (dd, *J* = 6.7, 3.9 Hz, 18H) ppm.

^{13}C NMR (175 MHz, CDCl$_3$): δ 209.7, 171.3, 141.8, 136.7, 134.6, 134.3, 129.6 (2C), 129.4 (2C), 128.9, 128.2, 128.0, 126.8, 64.4, 56.4, 32.5, 29.2, 28.8, 23.8, 21.1, 18.7 (3C), 18.7 (3C), 11.6 (3C).

HRMS (ESI+): calculated for $[M+Na]^+$ $C_{30}H_{44}NaO_3SSi^+$: 535.2673, found: 535.2671.

IR (cm^{-1}): 2940, 2866, 1740, 1689, 1466, 1233, 1036, 763, 689.

7-Bromo-3-(2-(phenylthio)phenyl)-1-(triisopropylsilyl)heptan-2-one (**4e**)

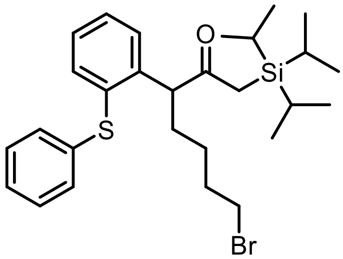

Prepared according to procedure C – 8 hours reaction time (42% yield).

^{1}H NMR (600 MHz, CDCl$_3$): δ 7.41 (d, *J* = 7.6 Hz, 1H), 7.31 – 7.25 (m, 4H), 7.25 – 7.18 (m, 4H), 4.56 – 4.51 (m, 1H), 3.25 (t, *J* = 6.9 Hz, 2H), 2.18 (d, *J* = 11.7 Hz, 1H), 1.97 – 1.91 (m, 1H), 1.89 (d, *J* = 11.9 Hz, 1H), 1.81 – 1.73 (m, 1H), 1.73 – 1.65 (m, 1H), 1.63 – 1.56 (m, 1H), 1.33 – 1.19 (m, 2H), 1.15 – 0.98 (m, 21H) ppm.

^{13}C NMR (150 MHz, CDCl$_3$): δ 209.7, 141.7, 136.7, 134.7, 134.3, 129.6 (2C), 129.4 (2C), 129.0, 128.3, 128.0, 126.8, 56.3, 33.6, 32.9, 32.0, 29.2, 26.1, 18.7 (3C), 18.6 (3C), 11.6 (3C).

HRMS (ESI+): calculated for $[M+Na]^+$ $C_{28}H_{41}BrOSSi^+$: 555.1723, found: 555.1722.

IR (cm^{-1}): 2940, 2866, 1691, 1466, 1438, 1246, 1201, 1024, 882, 735, 689.

2-(6-Oxo-5-(2-(phenylthio)phenyl)-7-(triisopropylsilyl)heptyl)isoindoline-1,3-dione (**4f**)

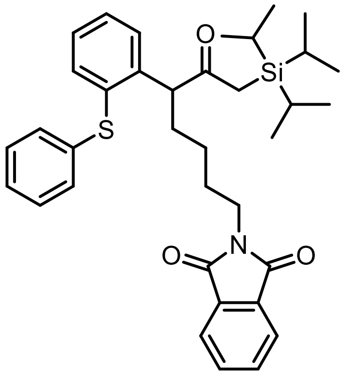

Prepared according to procedure C (41% yield).

^{1}H NMR (700 MHz, CDCl$_3$): δ 7.81 (dd, *J* = 5.4, 3.0 Hz, 2H), 7.69 (dd, *J* = 5.4, 3.0 Hz, 2H), 7.38 (dd, *J* = 7.7, 1.0 Hz, 1H), 7.28 – 7.22 (m, 3H), 7.22 – 7.18 (m, 4H), 7.17 – 7.14 (m, 1H), 4.55 – 4.47 (m, 1H), 3.52 (t, *J* = 7.5 Hz, 2H), 2.16 (d, *J* = 11.7 Hz, 1H), 1.99 – 1.92 (m, 1H), 1.88 (dd, *J* = 11.7, 0.8 Hz, 1H), 1.60 – 1.53 (m, 2H), 1.52 – 1.45 (m, 1H), 1.22 – 1.15 (m, 1H), 1.07 – 0.98 (m, 22H) ppm.

^{13}C NMR (175 MHz, CDCl$_3$): δ 209.6, 168.4 (2C), 141.9, 136.7, 134.7, 134.2, 133.9 (2C), 132.3 (2C), 129.6 (2C), 129.3 (2C), 129.0, 128.3, 127.9, 126.7, 123.2 (2C), 56.4, 37.9, 32.4, 29.2, 28.8, 24.9, 18.7 (3C), 18.7 (3C), 11.6 (3C) ppm.

HRMS (ESI+): calculated for $[M+Na]^+$ $C_{36}H_{45}NNaO_3SSi^+$: 622.2782, found: 622.2777.

IR (cm^{-1}): 2940, 2866, 1711, 1691, 1466, 1438, 1394, 1364, 1024, 882, 717, 689.

e. Competition experiments

With a mixture of sulfoxides

A solution of Tf_2NH (5.6 mg, 0.02 mmol, 0.1 equiv) in DCM (0.1 mL) was added to a solution of alkyne (39 mg, 0.2 mmol, 1 equiv), *p*-methyl diphenyl sulfoxide (46 mg, 0.2 mmol, 1 equiv) and *p*-chloro diphenyl sulfoxide (54 mg, 0.2 mmol, 1 equiv) in DCM (0.9 mL) at room temperature. The mixture was stirred at room temperature for 2 hours and quenched with saturated aqueous $NaHCO_3$. The aqueous layer was extracted 3 times with DCM, the combined organic layers were dried over $MgSO_4$ and concentrated under reduced pressure.

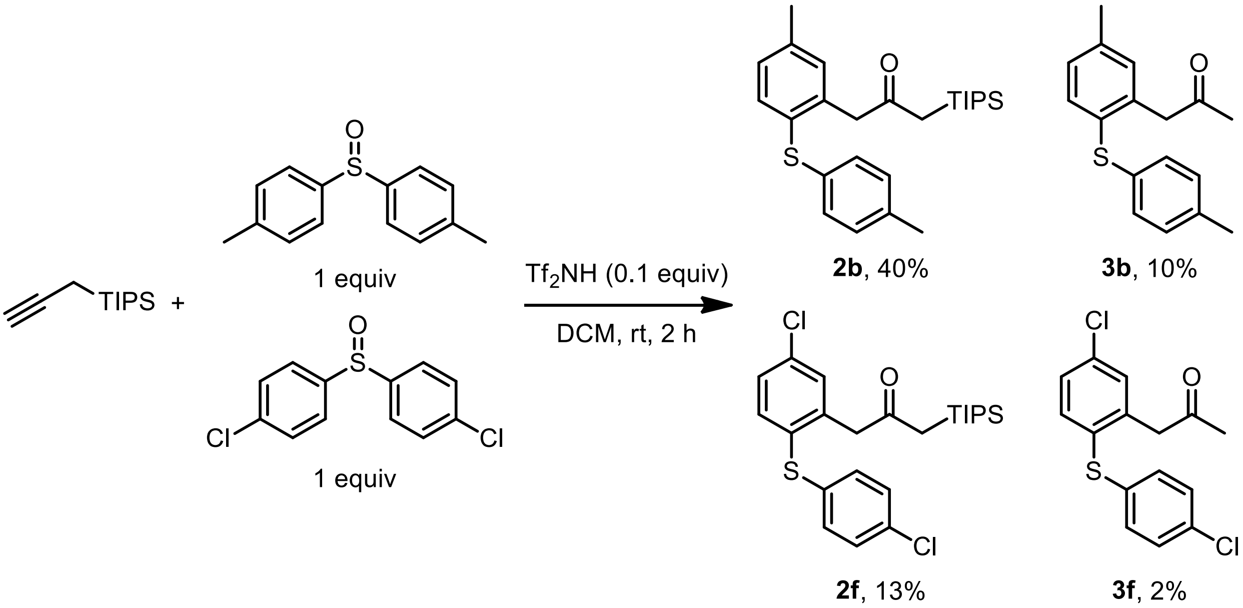

(**2b**+**3b**)/(**2f**+**3f**) = 77:23

This result suggests that the addition step of the sulfoxide onto the vinyl cation is faster for the most nucleophilic sulfoxide.

With an asymmetric sulfoxide

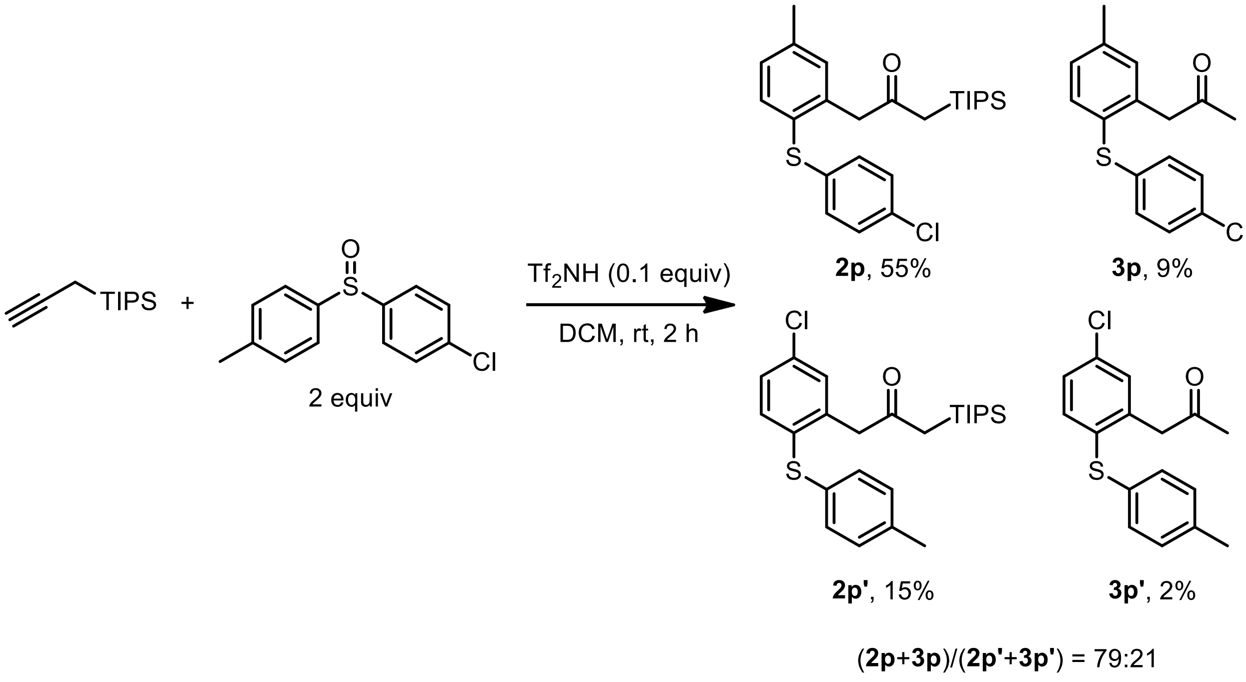

In this case, another mechanistic step is taken into consideration: this result indicates that the sulfonium rearrangement is faster onto the most electron-rich ring.

The results of competition experiments with a mixture of sulfoxides of different nucleophilicity as well as with an unsymmetrical diaryl sulfoxide with different aromatic substituents suggest that the overall reaction is faster as the sulfoxide gets more electron rich. However, we observed the opposite trend (Figure 1 of the main manuscript), which suggests that the Bronsted basicity of the sulfoxide plays a role in the mechanism. The alkyne is protonated by the protonated sulfoxide, then the same sulfoxide adds to the vinyl cation, and this is followed by rearrangement and deprotonation. The sulfoxide plays the role of a proton carrier and only then it acts as a nucleophile.
Hence the lower the basicity, the easier the proton transfer between the sulfoxide and the alkyne, hence the faster the reaction. The result of the intermolecular competition experiment with a mixture of sulfoxides shows that the most electron rich sulfoxide gets protonated preferentially. Even if it reacts more slowly, it still needs to be protonated for the reaction to be initiated. So the most basic sulfoxide is more likely to get captured because it is more likely to be protonated.
The results of the mechanistic experiments suggest that the alkyne protonation is the rate determining step. Calculations with selenoalkynes suggest a similar mechanism.[11]

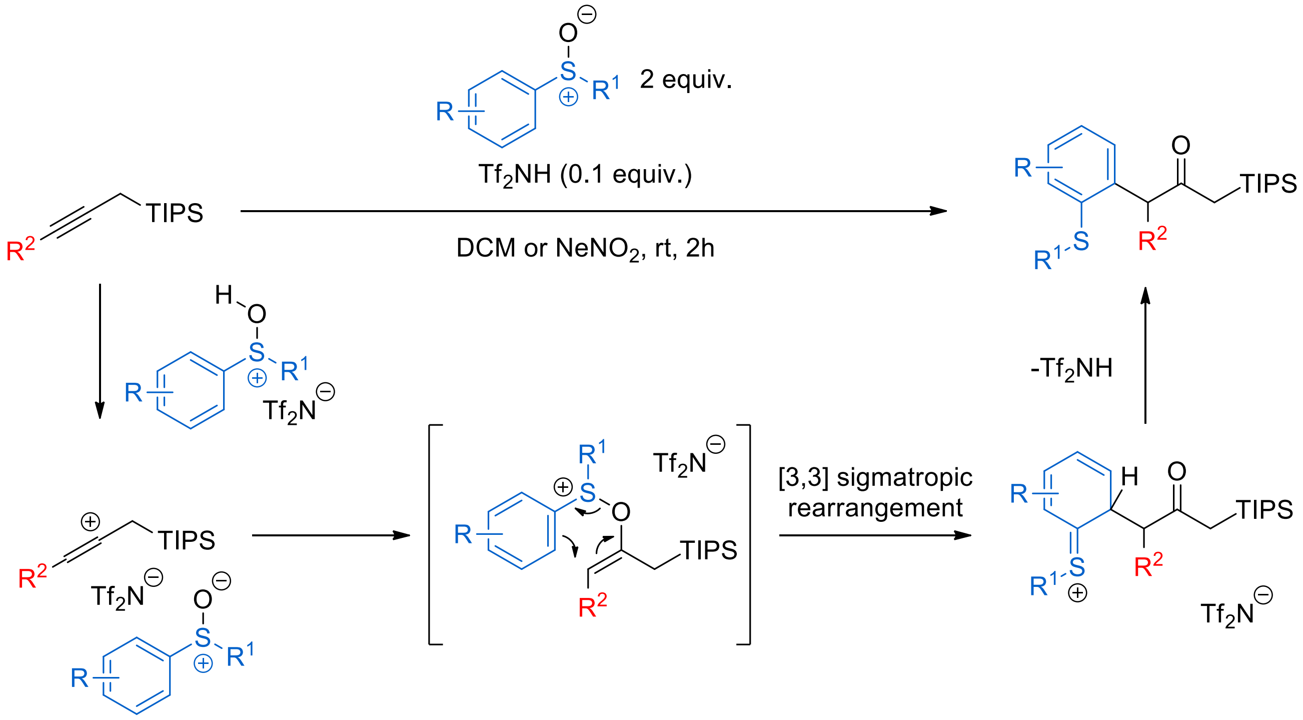

1-(2-((4-Chlorophenyl)thio)-5-methylphenyl)-3-(triisopropylsilyl)propan-2-one (**2p**)

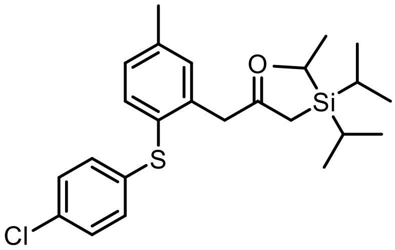

Prepared according to procedure B in $MeNO_2$ (22% yield).

^{1}H NMR (600 MHz, $CDCl_3$): δ 7.35 (d, *J* = 7.8 Hz, 1H), 7.17 (d, *J* = 8.5 Hz, 2H), 7.09 (s, 1H), 7.07 (d, *J* = 8.0 Hz, 1H), 6.98 (d, *J* = 8.5 Hz, 2H), 3.91 (s, 2H), 2.35 (s, 3H), 2.22 (s, 2H), 1.14 – 1.08 (m, 3H), 1.05 (d, *J* = 6.8 Hz, 18H) ppm.

^{13}C NMR (150 MHz, $CDCl_3$): δ 207.2, 139.6, 138.5, 136.9, 135.8, 132.3, 131.6, 129.3, 129.2 (2C), 129.1, 129.0 (2C), 49.9, 29.8, 21.4, 18.7 (6C), 11.6 (3C) ppm.

HRMS (ESI+): calculated for $[M+H]^+$ $C_{25}H_{36}ClOSSi^+$: 447.1939, found: 447.1937.

IR (cm^{-1}): 2941, 2865, 1694, 1473, 1387, 1314, 1231, 1191, 1091, 1043, 1010, 882, 811, 757, 697, 644.

1-(5-Chloro-2-(p-tolylthio)phenyl)-3-(triisopropylsilyl)propan-2-one (**2p'**)

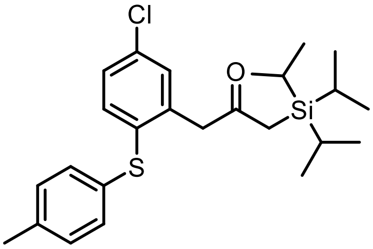

Prepared according to procedure B in $MeNO_2$ (7% yield).

^{1}H NMR (600 MHz, $CDCl_3$): δ 7.20 (m, 2H), 7.14 (dd, *J* = 8.4, 1.9 Hz, 1H), 7.09 (s, 4H), 3.92 (s, 2H), 2.31 (s, 3H), 2.26 (s, 2H), 1.18 – 1.11 (m, 3H), 1.07 (d, *J* = 7.0 Hz, 18H) ppm.

^{13}C NMR (150 MHz, $CDCl_3$): δ 206.3, 138.3, 137.2, 134.4, 134.2, 133.6, 132.2, 131.1, 130.5 (2C), 130.2 (2C), 128.2, 49.6, 29.9, 21.2, 18.7 (6C), 11.6 (3C) ppm.

HRMS (ESI+): calculated for $[M+H]^+$ $C_{25}H_{36}ClOSSi^+$: 447.1939, found: 447.1939.

IR (cm^{-1}): 2942, 2891, 2866, 1697, 1581, 1491, 1463, 1309, 1201, 1102, 1043, 1017, 883, 805, 745, 693, 666, 644.

3) Post-functionalization

4-Hydroxy-4-phenyl-1-(2-(phenylthio)phenyl)butan-2-one (**5**)

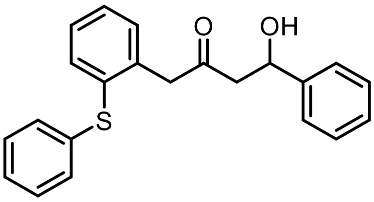

TMAF (9 mg, 0.1 mmol, 1 equiv) was added to a solution of α-silyl ketone **2a** (40 mg, 0.1 mmol, 1 equiv) and benzaldehyde (31 µL, 0.3 mmol, 3 equiv) in DMF (1 mL) at 0 °C. The mixture was stirred for 2 hours at 0 °C, then quenched with saturated aqueous $NaHCO_3$. EtOAc was added, the organic layer was washed 3 times with water, dried over $MgSO_4$ and concentrated under reduced pressure. The crude material was purified by silica gel column chromatography (heptane/EtOAc 8:2) to afford 16 mg (46% yield) of compound **5**.

^{1}H NMR (600 MHz, $CDCl_3$): δ 7.44 (d, J = 7.6 Hz, 1H), 7.37 – 7.30 (m, 5H), 7.29 – 7.23 (m, 5H), 7.22 – 7.17 (m, 1H), 7.17 – 7.13 (m, 2H), 5.13 – 5.06 (m, 1H), 3.95 (d, J = 16.8 Hz, 1H), 3.90 (d, J = 16.8 Hz, 1H), 3.27 (d, J = 2.5 Hz, 1H), 2.85 (dd, J = 17.6, 9.1 Hz, 1H), 2.79 (dd, J = 17.6, 3.2 Hz, 1H) ppm.

^{13}C NMR (150 MHz, $CDCl_3$): δ 208.4, 142.8, 136.7, 136.4, 134.8, 134.1, 131.7, 129.4 (2C), 129.3 (2C), 128.8, 128.6 (2C), 128.6, 127.7, 126.7, 125.8 (2C), 70.0, 51.0, 49.0 ppm.

HRMS (ESI+): calculated for $[M+H]^+$ $C_{22}H_{21}O_2Si^+$: 349.1257, found: 349.1251.

IR (cm^{-1}): 3419, 3058, 3030, 2923, 1709, 1581, 1493, 1439, 1403, 1327, 1195, 1157, 1057, 1024, 910, 739, 694.

3-(2-(Phenylthio)phenyl)-1-(triisopropylsilyl)butan-2-one (**6**)

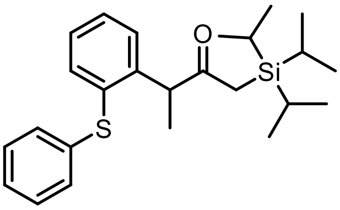

To a solution of NaHMDS (2.0 M in THF, 0.12 mL, 0.24 mmol, 1.2 equiv.) in anhydrous THF (1.5 mL) at -78 C was added dropwise a solution of **2a** (79.7 mg, 0.2 mmol, 1 equiv.) in anhydrous THF (0.5 mL). The reaction mixture was was stirred at -78 °C for 30 min, then warmed up to 0 °C and stirred for 45 min. Then MeI (31.4 µL, 0.5 mmol, 2.5 equiv.) was added at 0 °C, the reaction mixture was stirred at 0 °C for further 60 minutes, then it was allowed to warm up to room temperature and stirred for 24 h. The reaction mixture was quenched with saturated aqueous solution of $NaHCO_3$. The reaction mixture was extracted with DCM 3 times, dried over $MgSO_4$ and concentrated under reduced pressure. The crude material was purified by silica gel column chromatography (heptane/EtOAc 97:3 to 95:5) to afford 70 mg (85%) of compound **6**.
^{1}H NMR (400 MHz, $CDCl_3$) δ 7.39 – 7.30 (m, 1H), 7.26 – 7.08 (m, 8H), 4.52 (qd, J = 6.7, 1.1 Hz, 1H), 2.17 – 2.07 (m, 1H), 1.85 (dd, J = 11.5, 1.3 Hz, 1H), 1.15 (d, J = 6.8 Hz, 3H), 1.06 – 0.98 (m, 3H), 0.97 – 0-93 (m, 18H) ppm.
^{13}C NMR (101 MHz, $CDCl_3$) δ 210.3, 143.8, 136.9, 134.9, 133.5, 129.4 (2C), 129.4 (2C), 129.1, 128.3, 128.0, 126.6, 51.0, 29.0, 18.6, 18.0, 11.6 ppm.
HRMS (ESI+): calculated for $[M+H]^+$ $C_{25}H_{37}OSSi^+$: 413.2329, found: 413.2327.
IR (cm^{-1}): 2941, 2865, 1692, 1468, 1023, 882, 735, 689.

1-Phenyl-3-(triisopropylsilyl)propan-2-one (**7**)

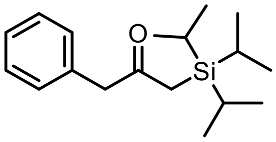

To a solution of **2a** (341 mg, 0.855 mmol) in methanol (10 mL) was added an excess of Raney 2800 Ni (washed with methanol three times just prior to use; Caution! Raney Ni is pyrophoric if allowed to dry out). The resulting mixture was stirred vigorously at room temperature for 48 h under an atmosphere of H_2 (1atm). The progress of the reaction was monitored by ^{1}H NMR. The mixture was diluted with EtOAc and filtered over a pad of Celite®, and the filter cake was washed with copious amount of EtOAc. The Raney Ni was washed with EtOAc. The combined solution was concentrated and purified by silica flash column chromatography (heptane/EtOAc 97:3 to 95:5) to afford 204 mg (82% yield) of compound **7**.
^{1}H NMR (400 MHz, $CDCl_3$) δ 7.37 – 7.16 (m, 5H), 3.73 (s, 2H), 2.24 (s, 2H), 1.20 – 1.11 (m, 3H), 1.09 – 1.07 (d, J = 6.0 Hz 18H).
^{13}C NMR (101 MHz, $CDCl_3$) δ 207.7, 134.9, 129.6 (2C), 128.7 (2C), 127.0, 52.1, 29.2, 18.7, 11.6.
HRMS (ESI+): calculated for $[M+Na]^+$ $C_{18}H_{30}OSiNa^+$: 313.1958, found: 313.1957.
IR (cm^{-1}): 2941, 2980, 2865, 1693, 1463, 1242, 1110, 1043, 882, 732, 696, 663.

NMR spectra

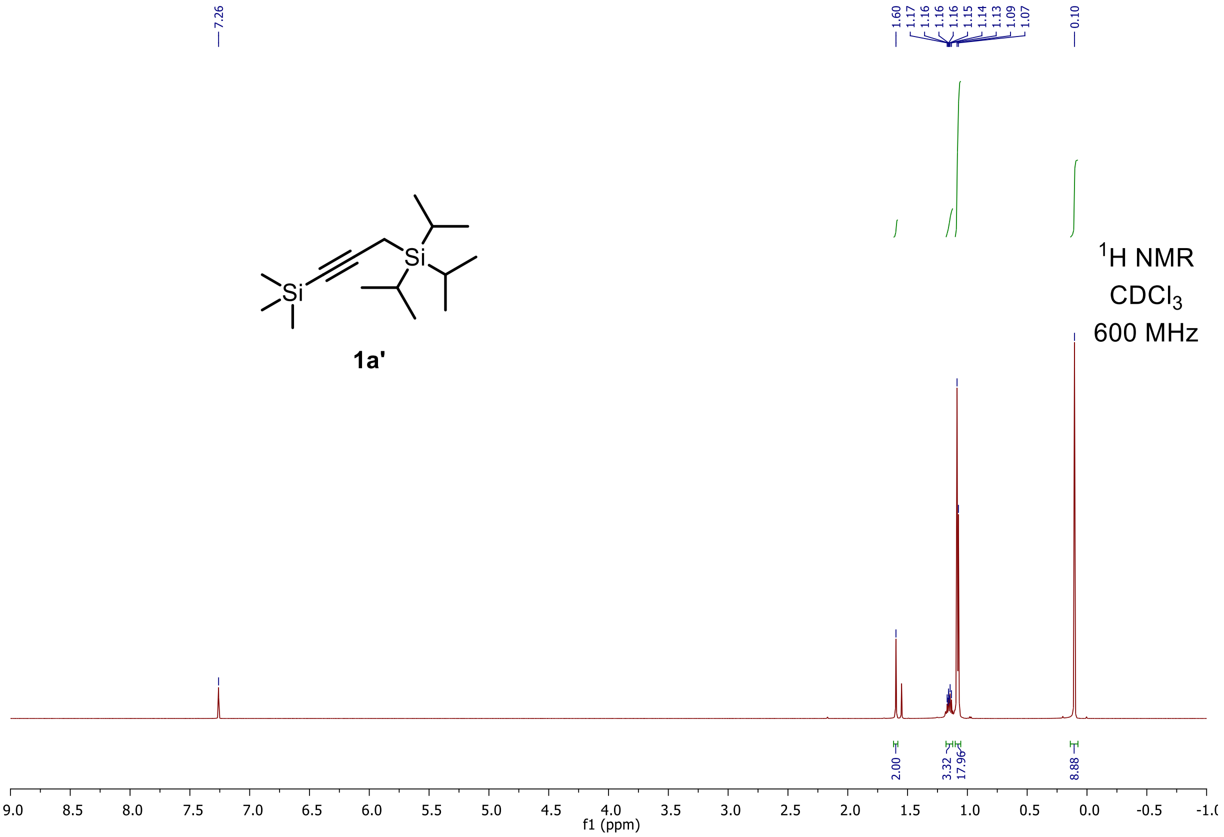
7.26
1.60
1.17
1.16
1.16
1.16
1.15
1.14
1.13
1.09
1.07
0.10
Si
Si
1a'
^{1}H NMR
$CDCl_3$
600 MHz
2.00
3.32
17.96
8.88
9.0 8.5 8.0 7.5 7.0 6.5 6.0 5.5 5.0 4.5 4.0 3.5 3.0 2.5 2.0 1.5 1.0 0.5 0.0 -0.5
f1 (ppm)

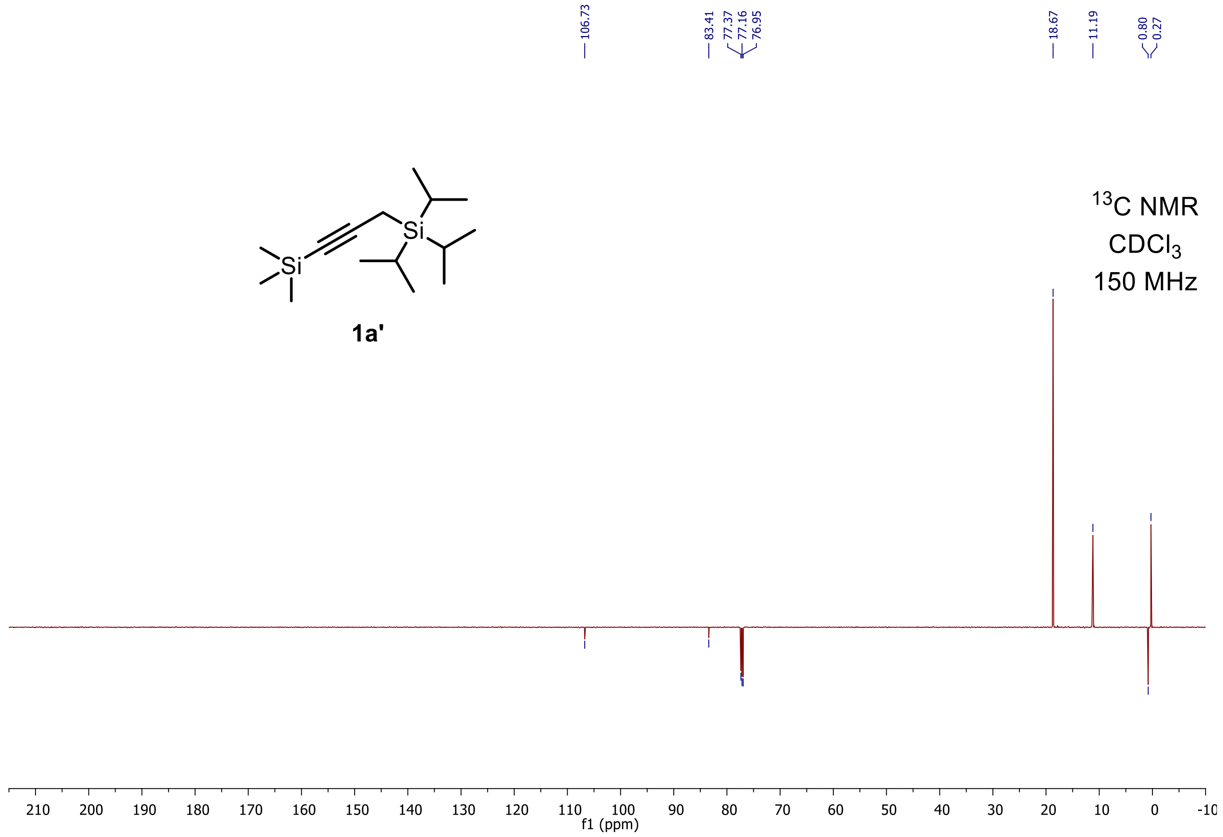
106.73
83.41
77.37
77.16
76.95
18.67
11.19
0.80
0.27
Si
Si
1a'
^{13}C NMR
$CDCl_3$
150 MHz
210 200 190 180 170 160 150 140 130 120 110 100 90 80 70 60 50 40 30 20 10 0
f1 (ppm)

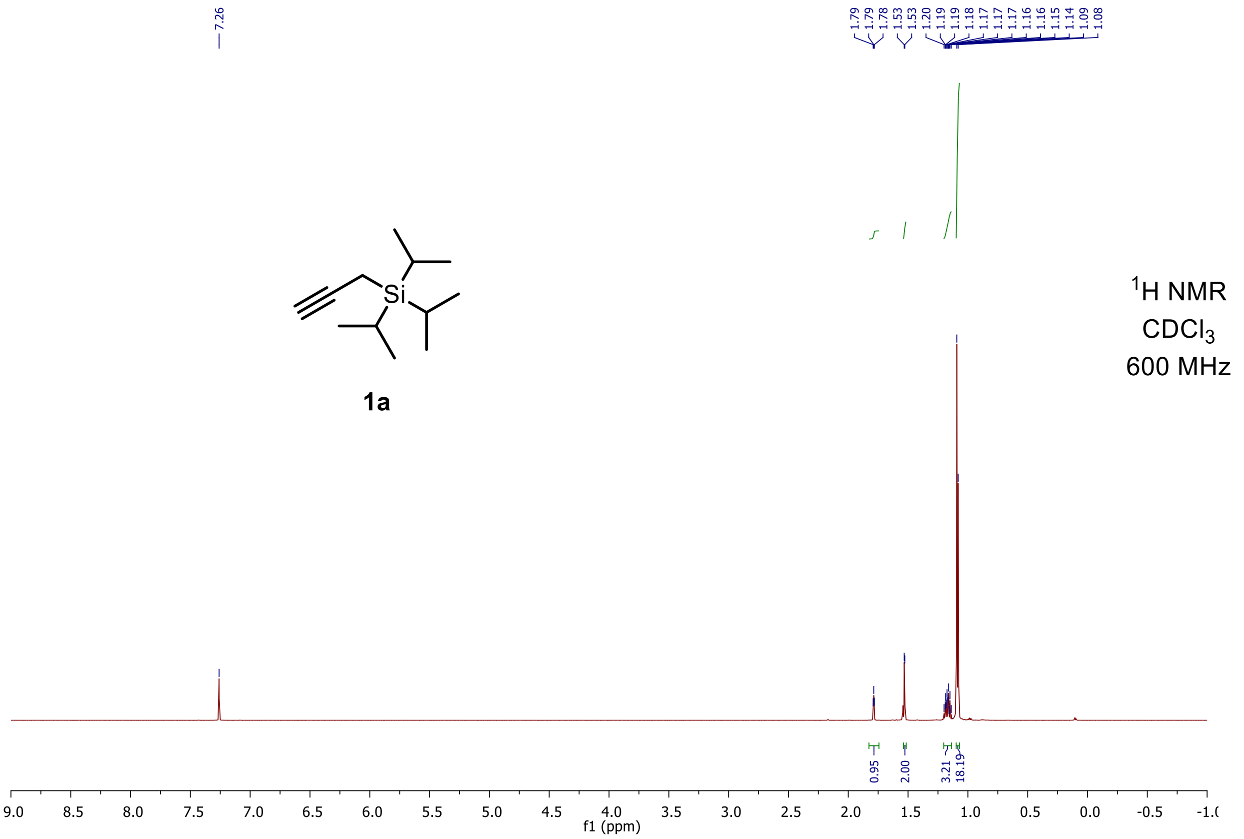
7.26
1.79
1.79
1.78
1.53
1.53
1.20
1.19
1.19
1.18
1.17
1.17
1.17
1.16
1.15
1.14
1.09
1.08
Si
1a
^{1}H NMR
$CDCl_3$
600 MHz
0.95
2.00
3.21
18.19
9.0 8.5 8.0 7.5 7.0 6.5 6.0 5.5 5.0 4.5 4.0 3.5 3.0 2.5 2.0 1.5 1.0 0.5 0.0 -0.5
f1 (ppm)

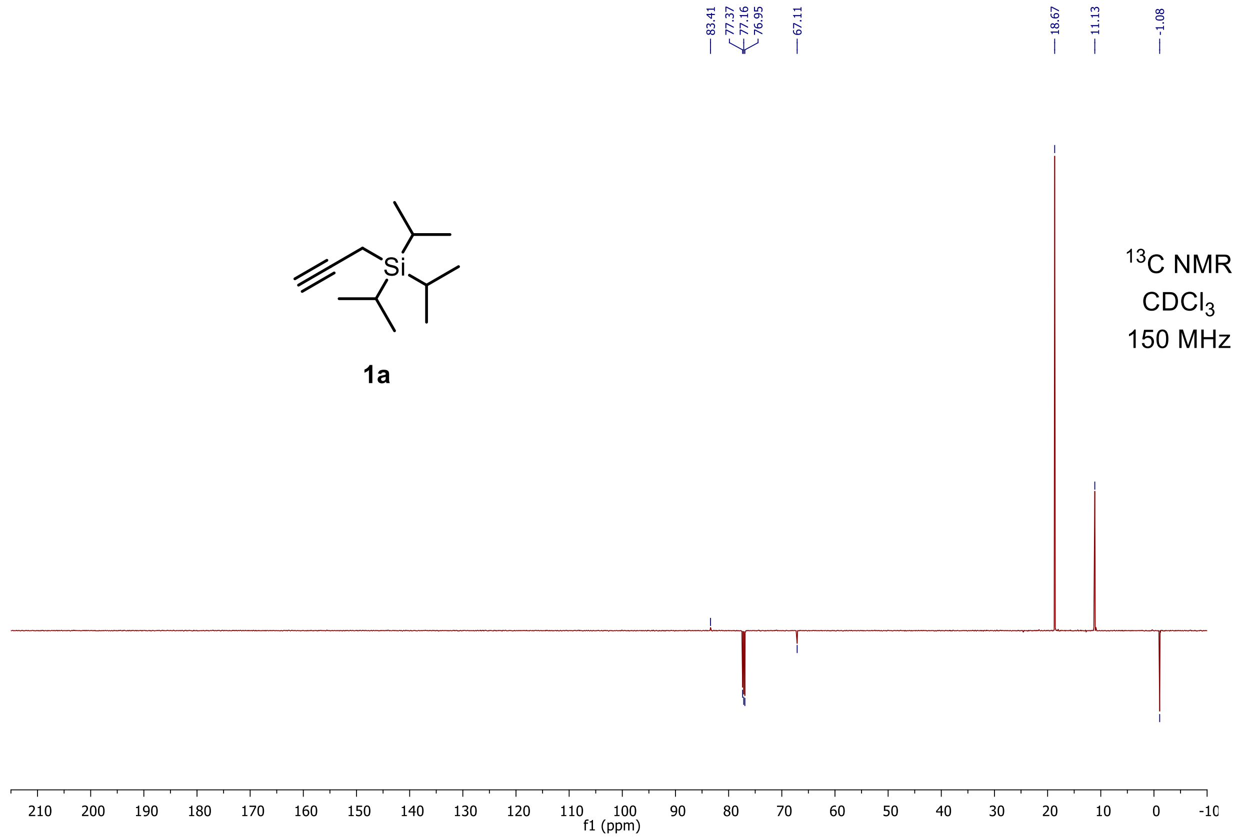
83.41
77.37
77.16
76.95
67.11
18.67
11.13
-1.08
Si
1a
^{13}C NMR
$CDCl_3$
150 MHz
210 200 190 180 170 160 150 140 130 120 110 100 90 80 70 60 50 40 30 20 10 0
f1 (ppm)

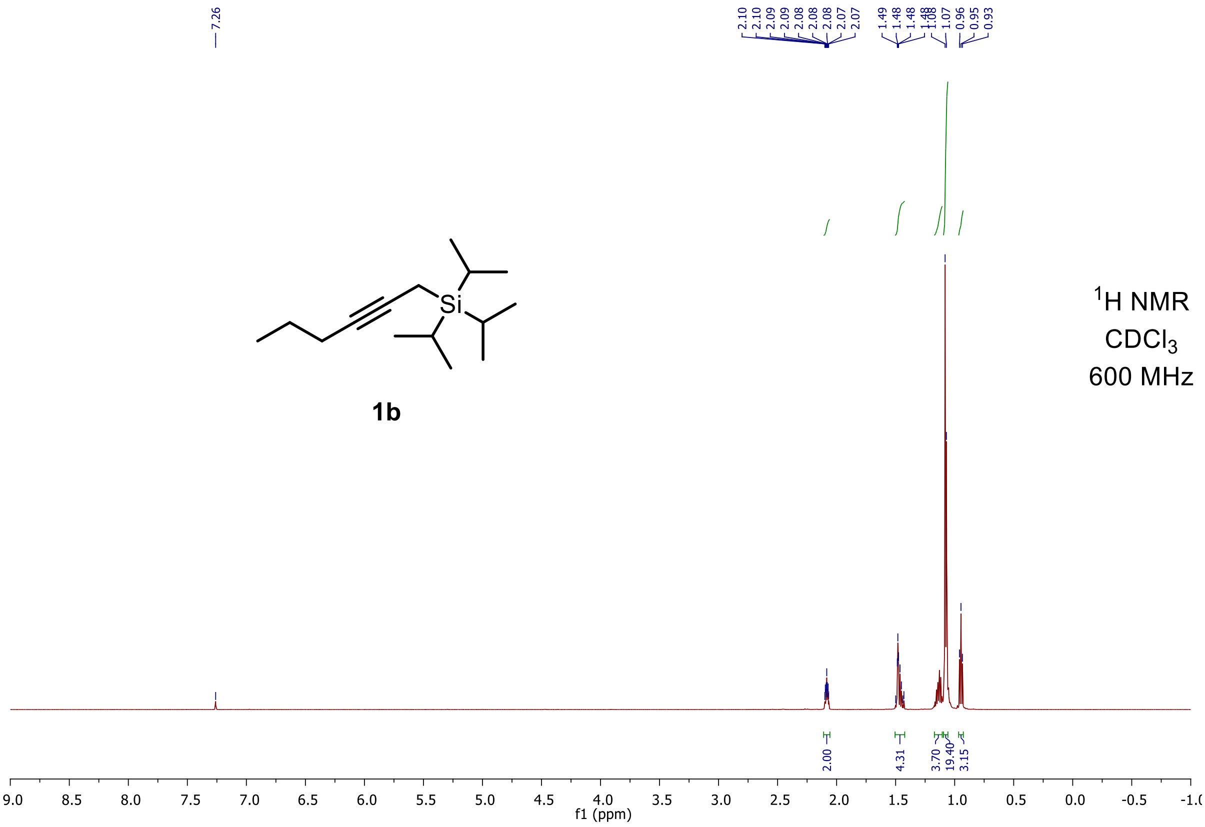
1b
1H NMR
CDCl3
600 MHz
f1 (ppm)

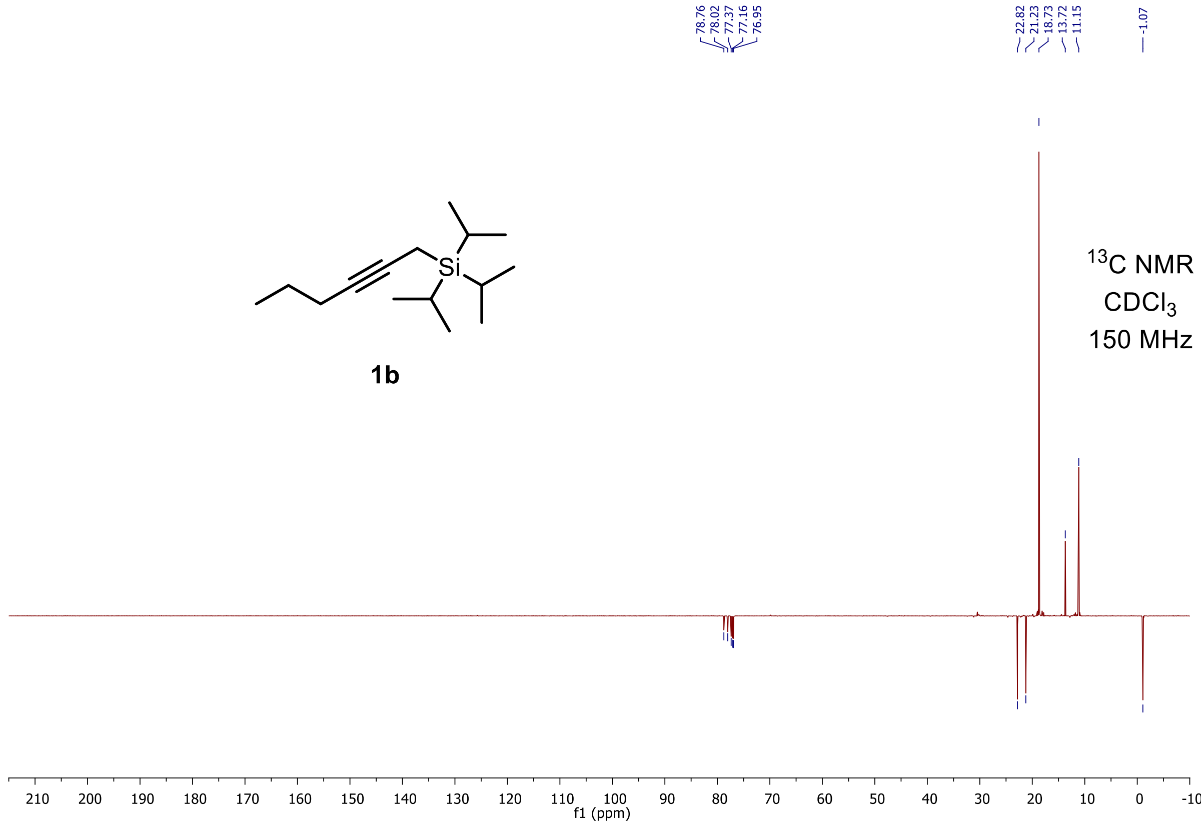
1b
13C NMR
CDCl3
150 MHz
f1 (ppm)

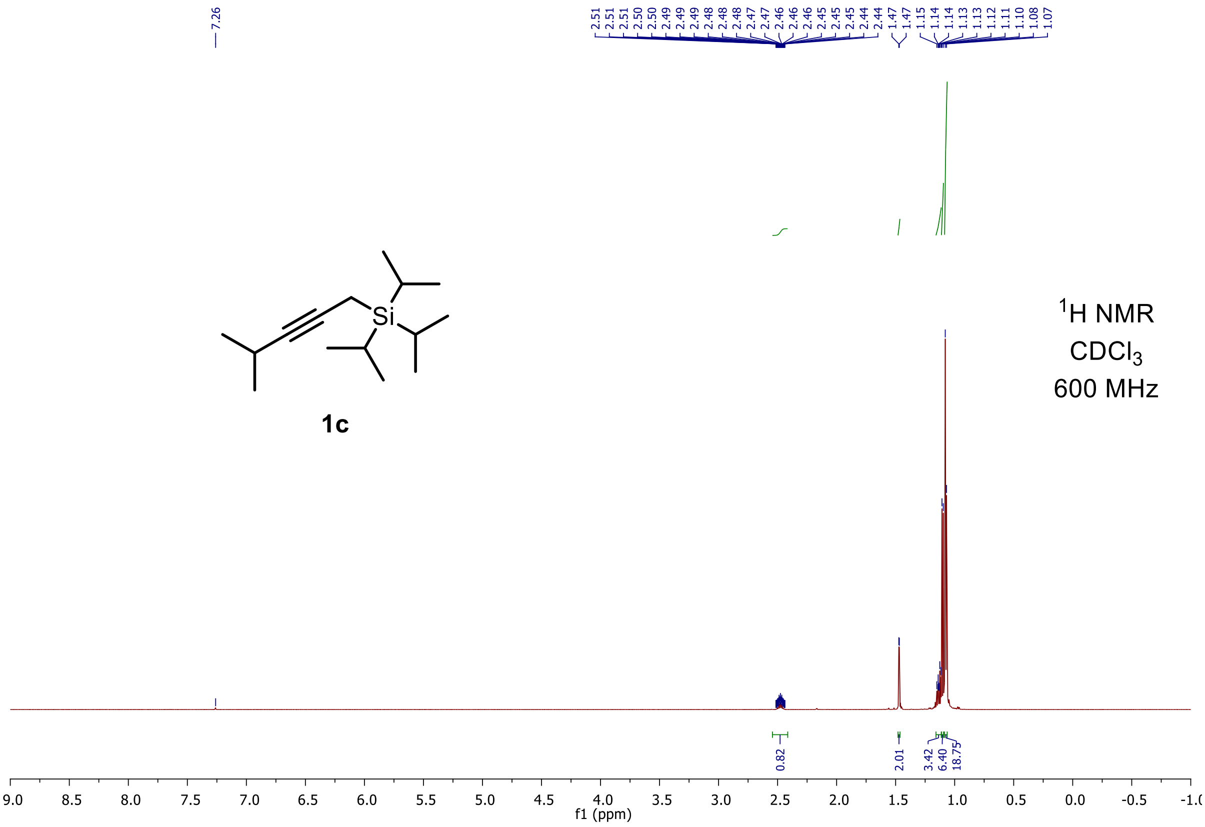

1c
1H NMR
CDCl3
600 MHz
7.26
2.51 2.51 2.51 2.50 2.50 2.49 2.49 2.49 2.48 2.48 2.48 2.48 2.47 2.47 2.46 2.46 2.46 2.45 2.45 2.45 2.44 2.44 1.47 1.47 1.15 1.14 1.14 1.13 1.13 1.12 1.11 1.10 1.08 1.07
0.82
2.01
3.42
6.40
18.75
f1 (ppm)

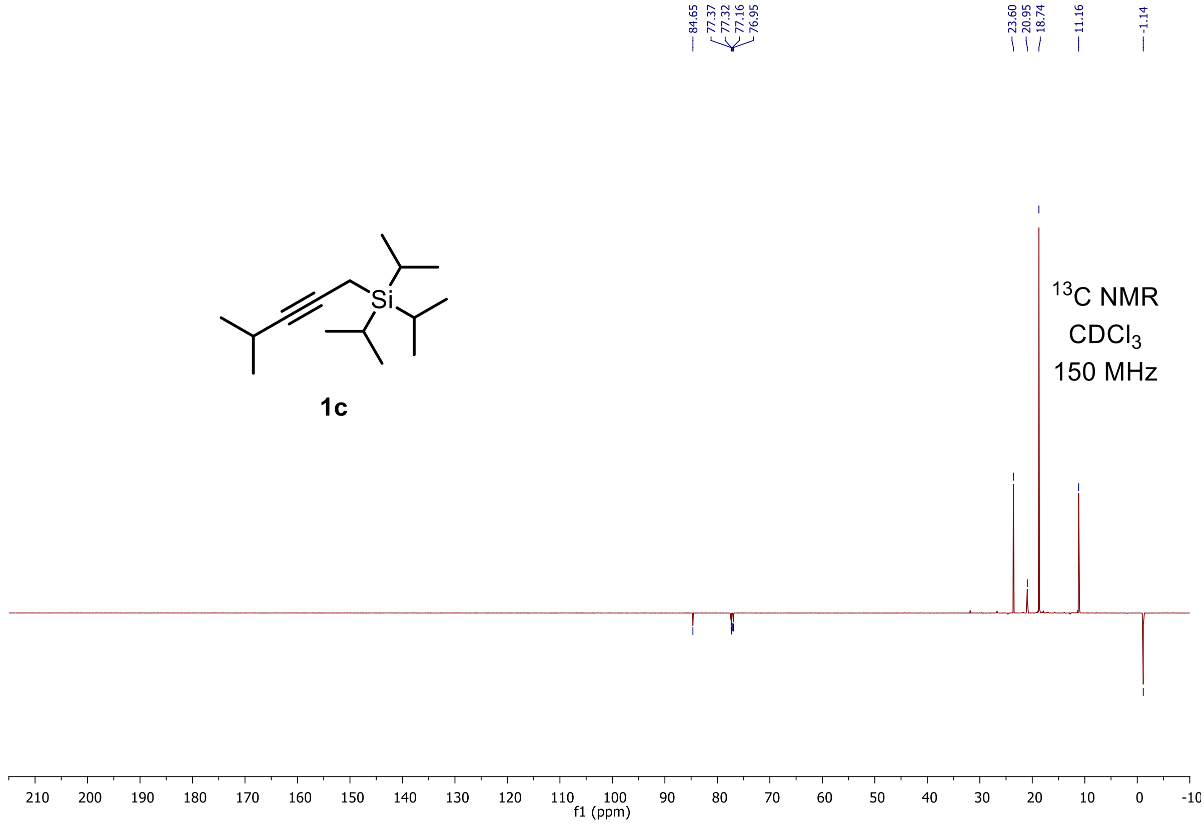

1c
13C NMR
CDCl3
150 MHz
84.65 77.37 77.32 77.16 76.95
23.60 20.95 18.74 11.16 -1.14
f1 (ppm)

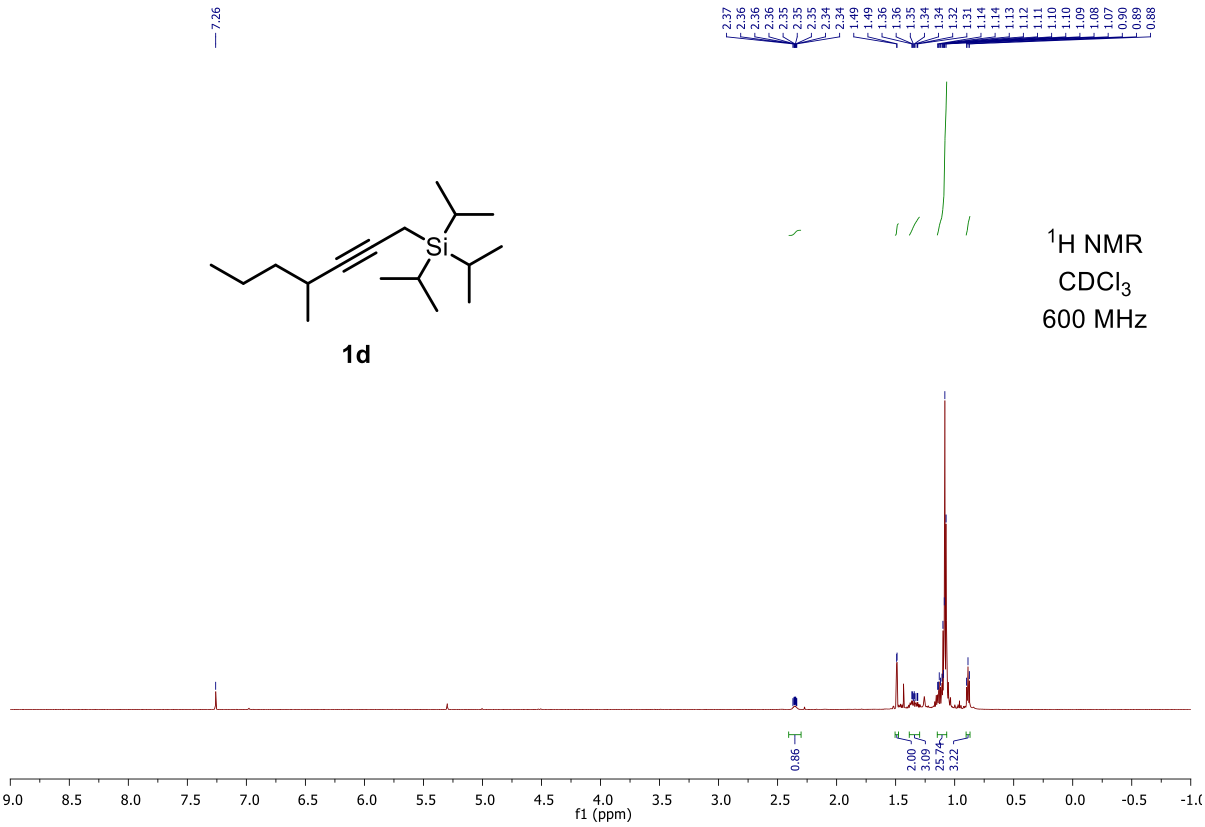
7.26
2.37 2.36 2.36 2.36 2.35 2.35 2.35 2.34 2.34 1.49 1.49 1.36 1.36 1.35 1.34 1.34 1.34 1.32 1.31 1.14 1.14 1.13 1.12 1.11 1.10 1.10 1.09 1.08 1.07 0.90 0.89 0.88
Si
1d
^{1}H NMR
$CDCl_3$
600 MHz
0.86
2.00
3.09
25.74
3.22
9.0 8.5 8.0 7.5 7.0 6.5 6.0 5.5 5.0 4.5 4.0 3.5 3.0 2.5 2.0 1.5 1.0 0.5 0.0 -0.5
f1 (ppm)

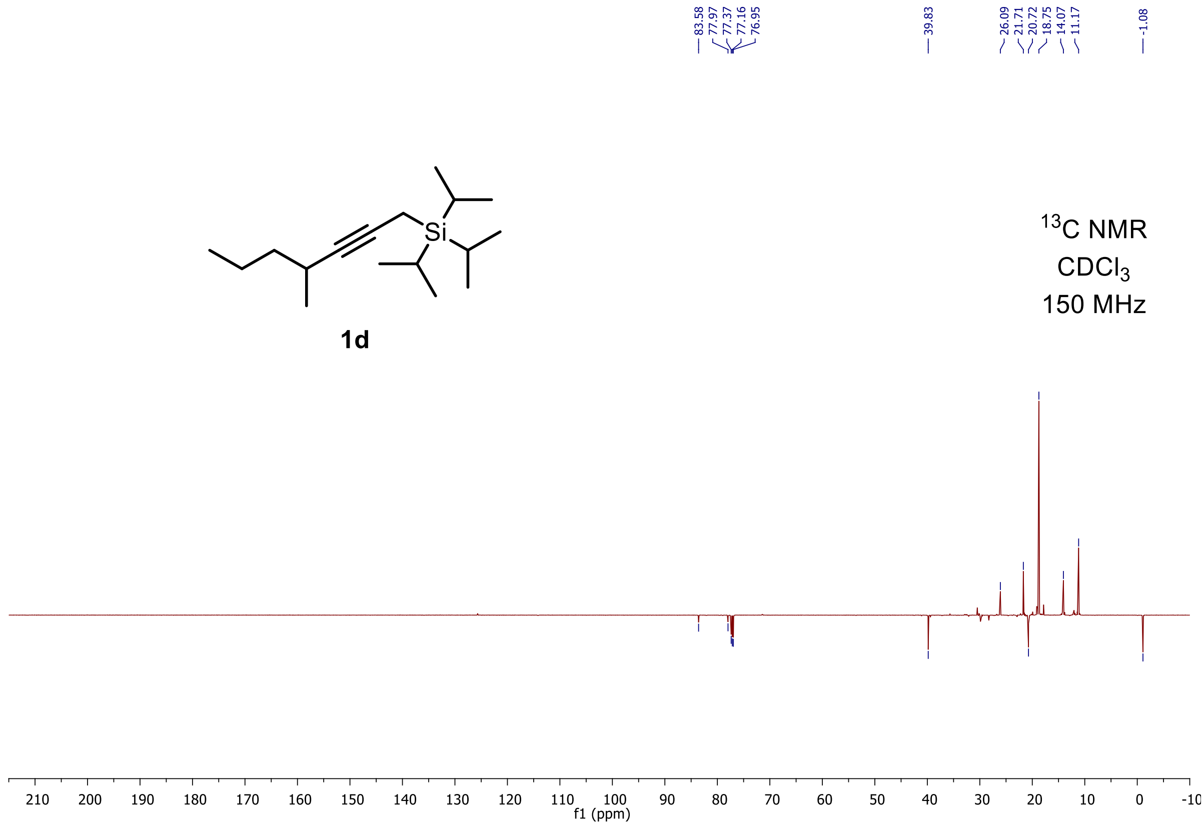
83.58 77.97 77.37 77.16 76.95
39.83
26.09 21.71 20.72 18.75 14.07 11.17
-1.08
Si
1d
^{13}C NMR
$CDCl_3$
150 MHz
210 200 190 180 170 160 150 140 130 120 110 100 90 80 70 60 50 40 30 20 10 0
f1 (ppm)

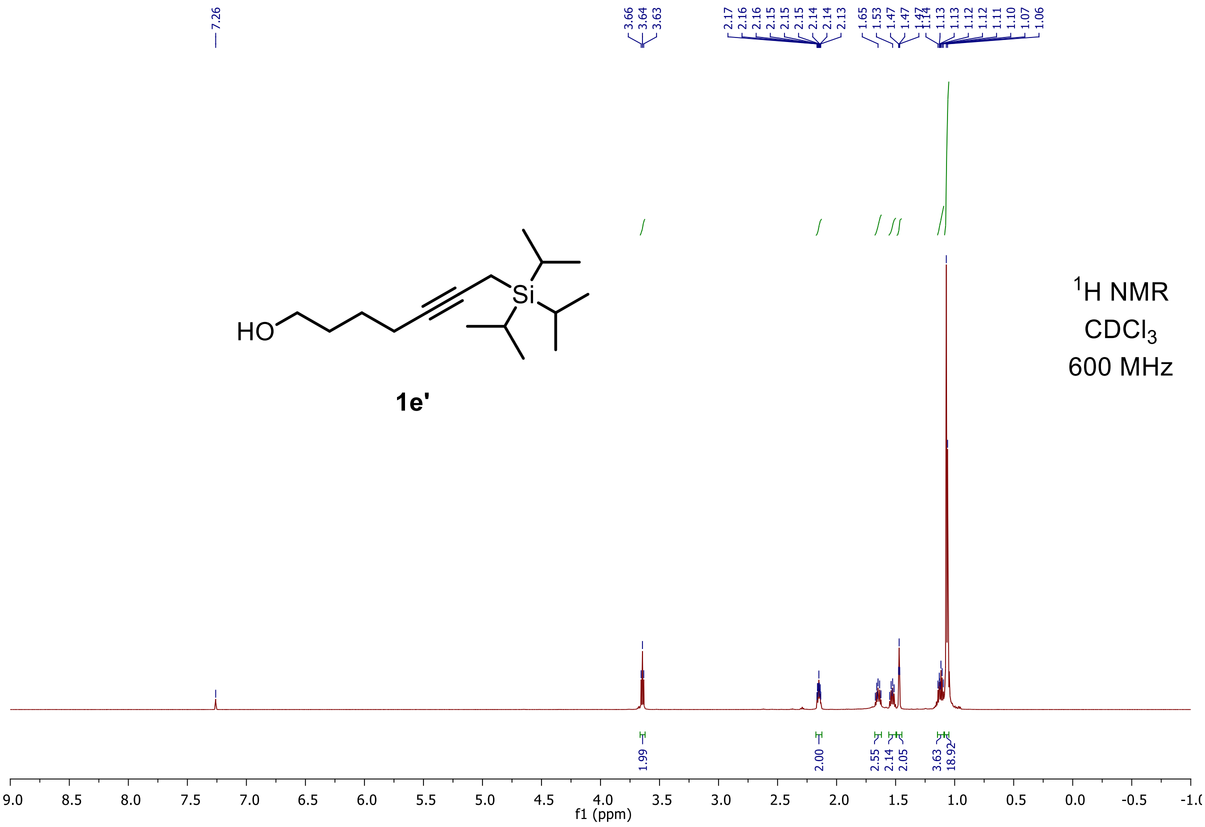
7.26
3.66
3.64
3.63
2.17
2.16
2.16
2.15
2.15
2.15
2.14
2.14
2.13
1.65
1.53
1.47
1.47
1.14
1.13
1.13
1.12
1.12
1.11
1.10
1.07
1.06
HO
Si
1e'
^{1}H NMR
$CDCl_3$
600 MHz
1.99
2.00
2.55
2.14
2.05
3.63
18.92
f1 (ppm)

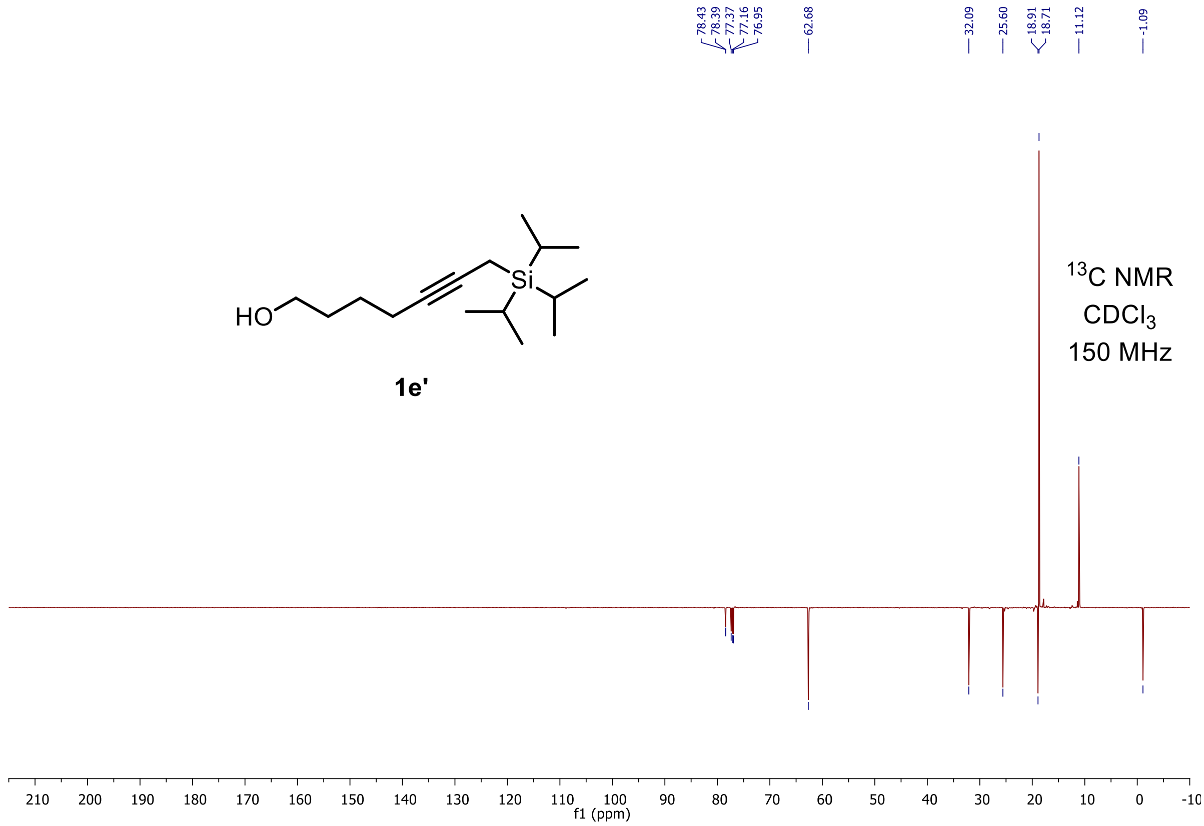
78.43
78.39
77.37
77.16
76.95
62.68
32.09
25.60
18.91
18.71
11.12
-1.09
HO
Si
1e'
^{13}C NMR
$CDCl_3$
150 MHz
f1 (ppm)

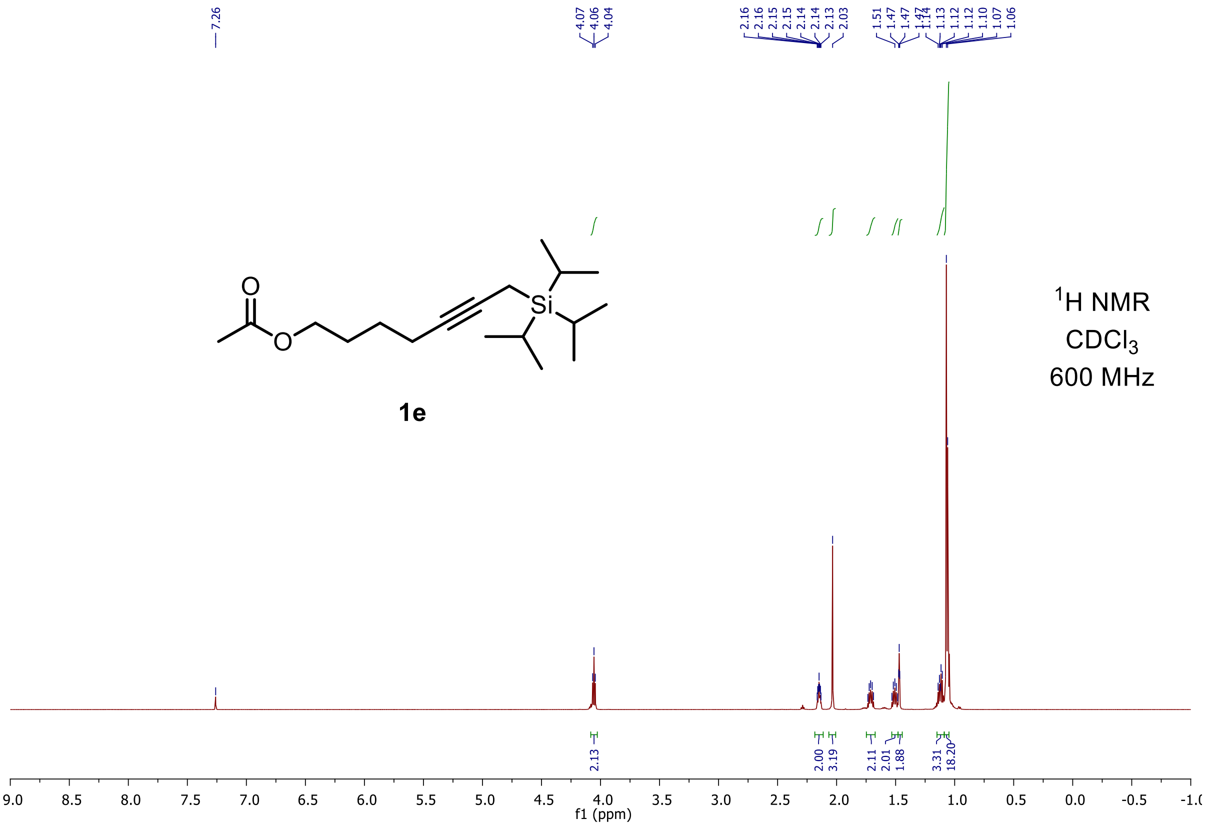
7.26
4.07
4.06
4.04
2.03
Si
O
O
1e
^{1}H NMR
CDCl$_3$
600 MHz
2.13
2.00
3.19
2.11
2.01
1.88
3.31
18.20
f1 (ppm)

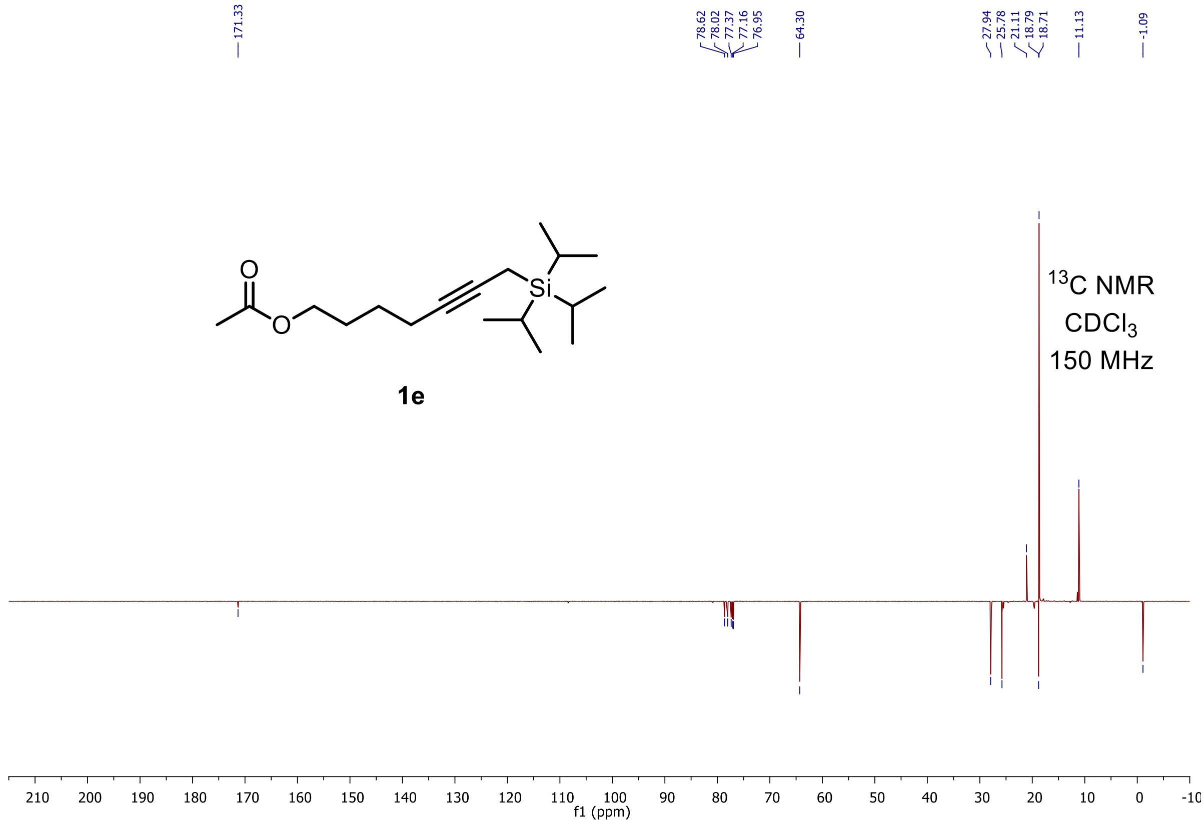
171.33
78.62
78.02
77.37
77.16
76.95
64.30
27.94
25.78
21.11
18.79
18.71
11.13
-1.09
Si
O
O
1e
^{13}C NMR
CDCl$_3$
150 MHz
f1 (ppm)

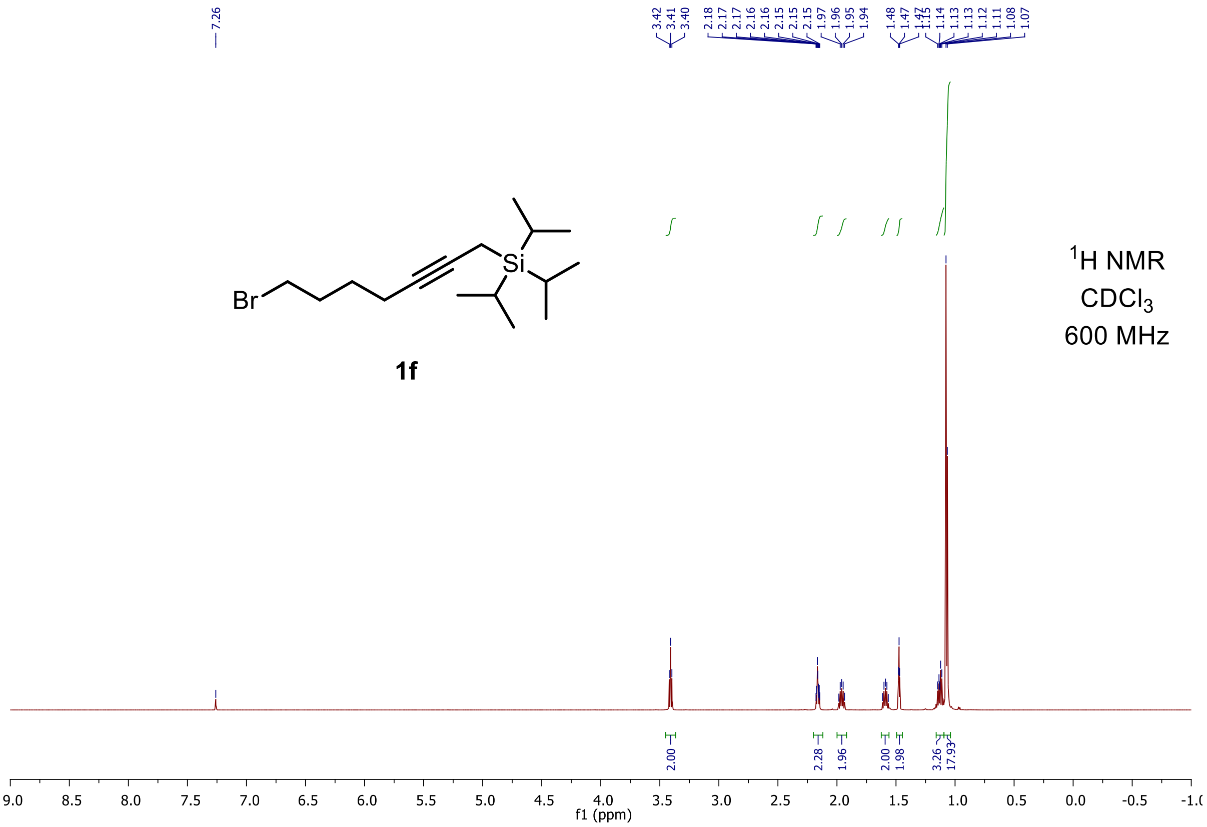

Br
Si
1f
^{1}H NMR
$CDCl_3$
600 MHz
f1 (ppm)

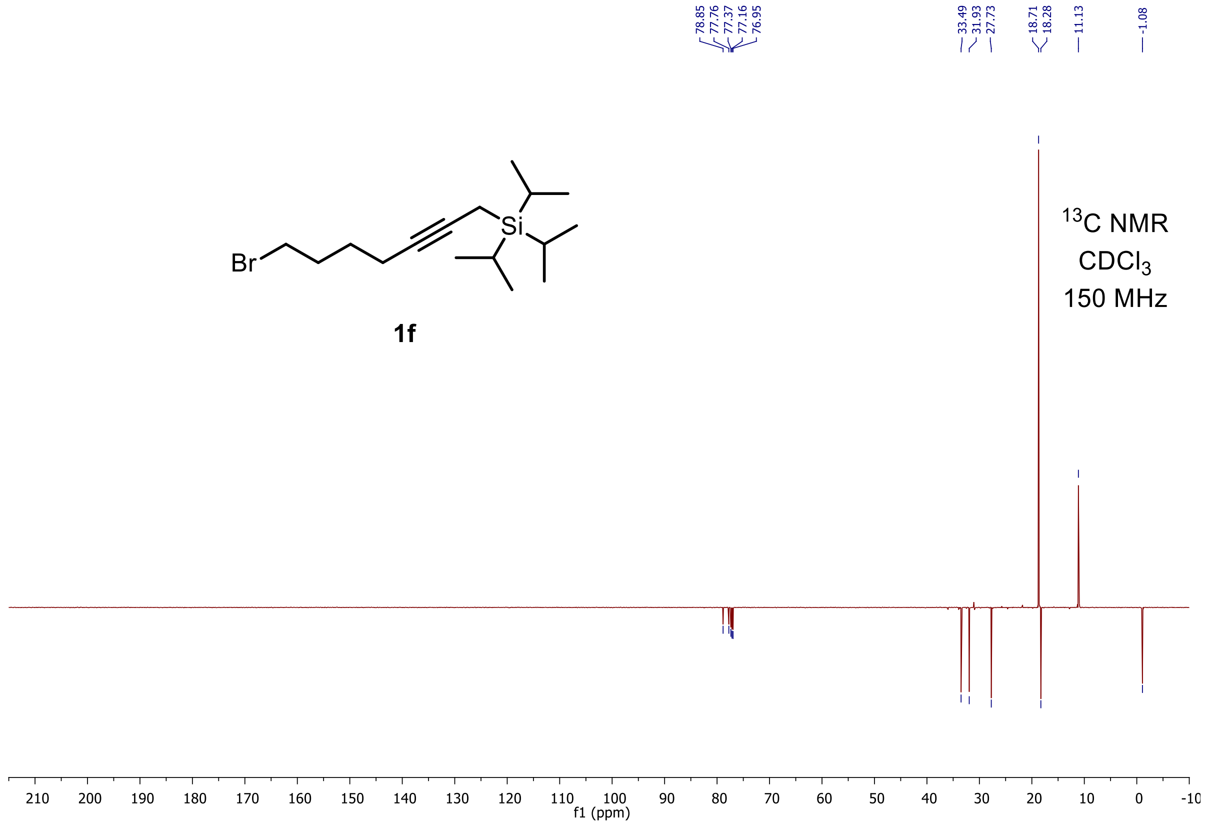

Br
Si
1f
^{13}C NMR
$CDCl_3$
150 MHz
f1 (ppm)

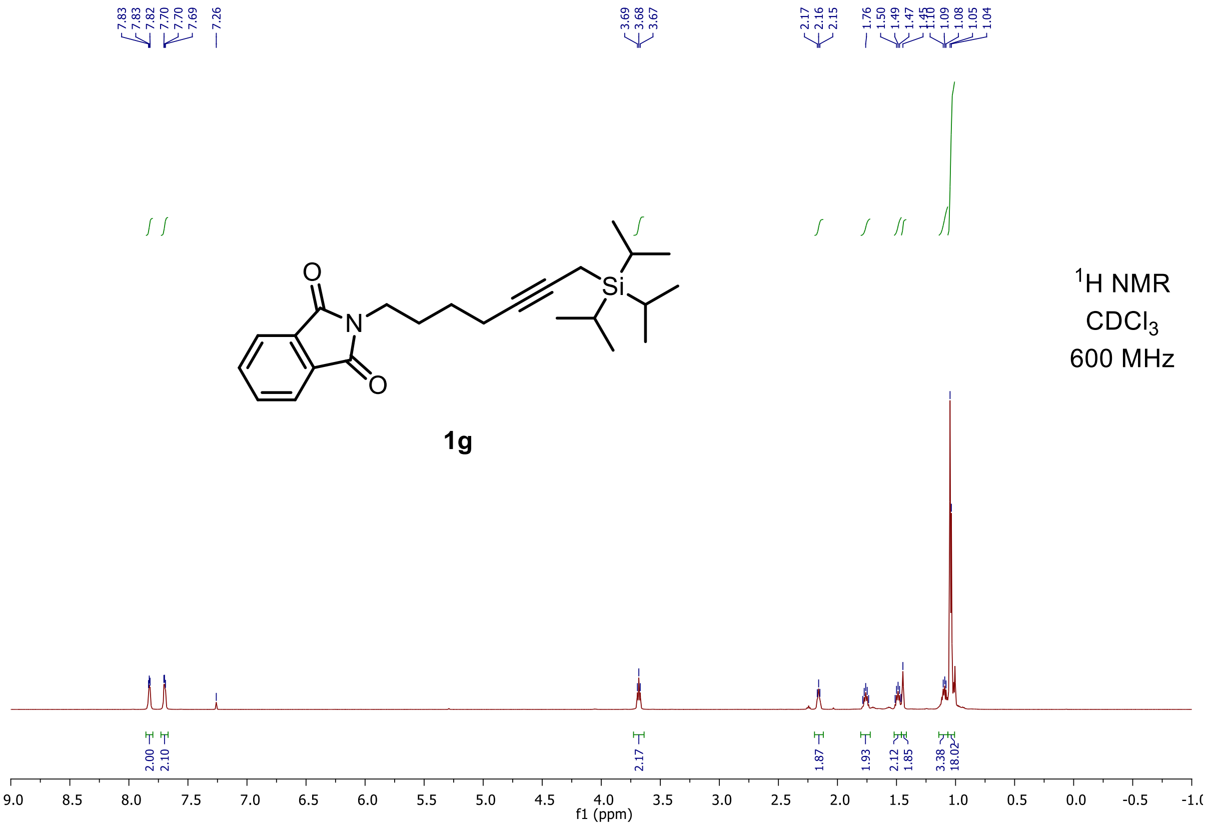
1g
^{1}H NMR
CDCl$_3$
600 MHz
f1 (ppm)

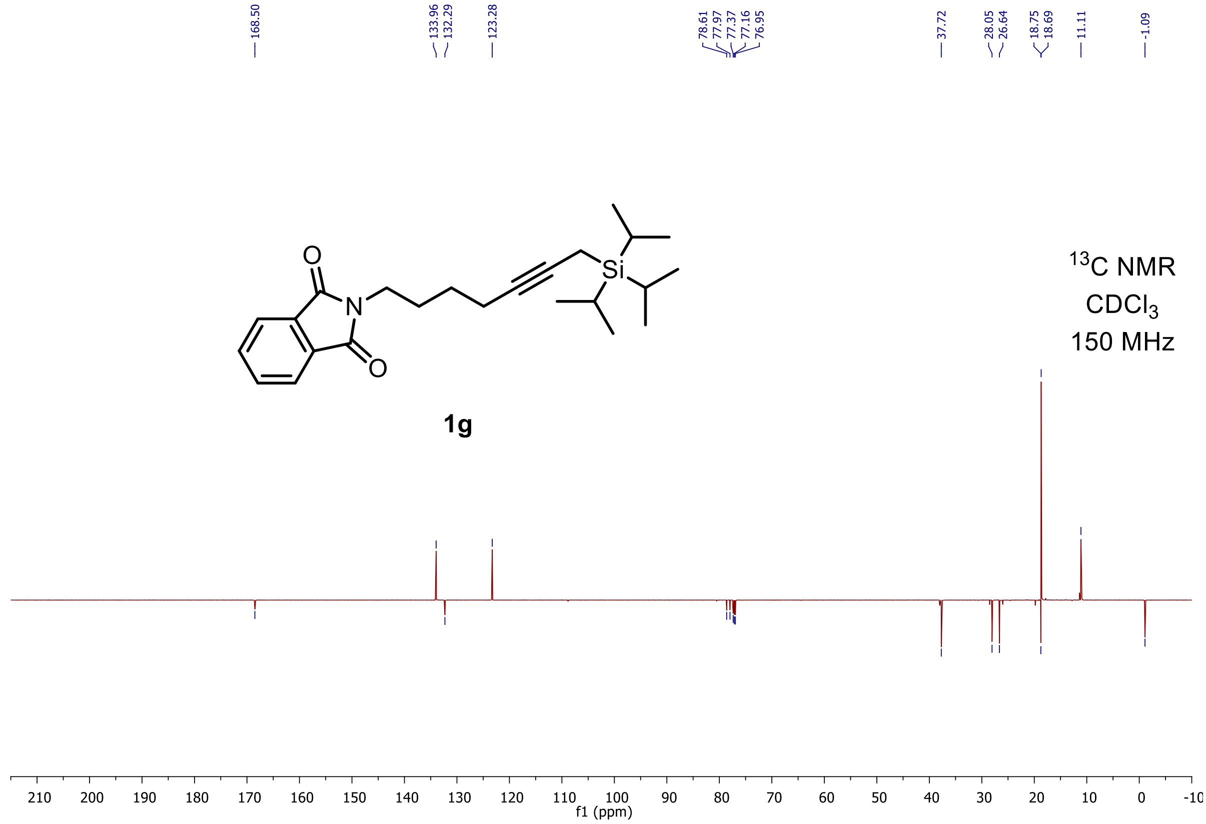
1g
^{13}C NMR
CDCl$_3$
150 MHz
f1 (ppm)

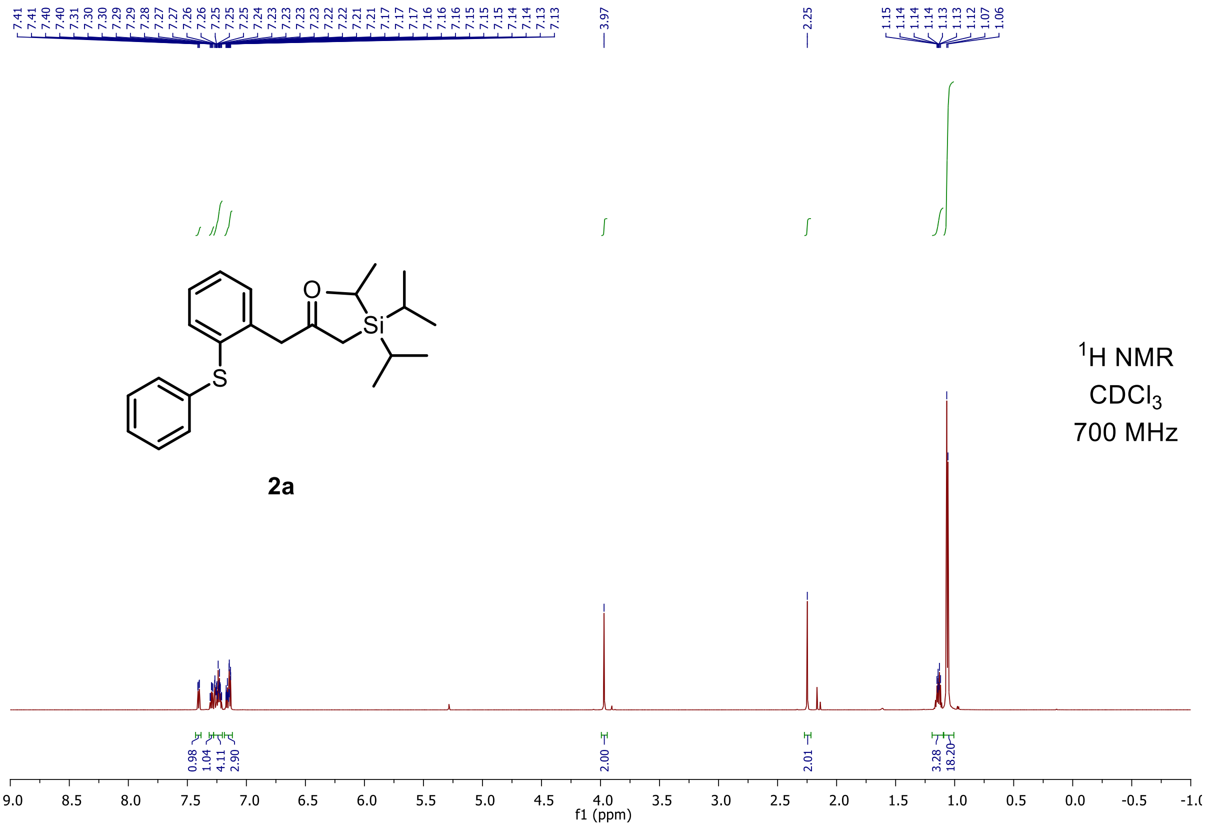

2a
^{1}H NMR
$CDCl_3$
700 MHz
f1 (ppm)

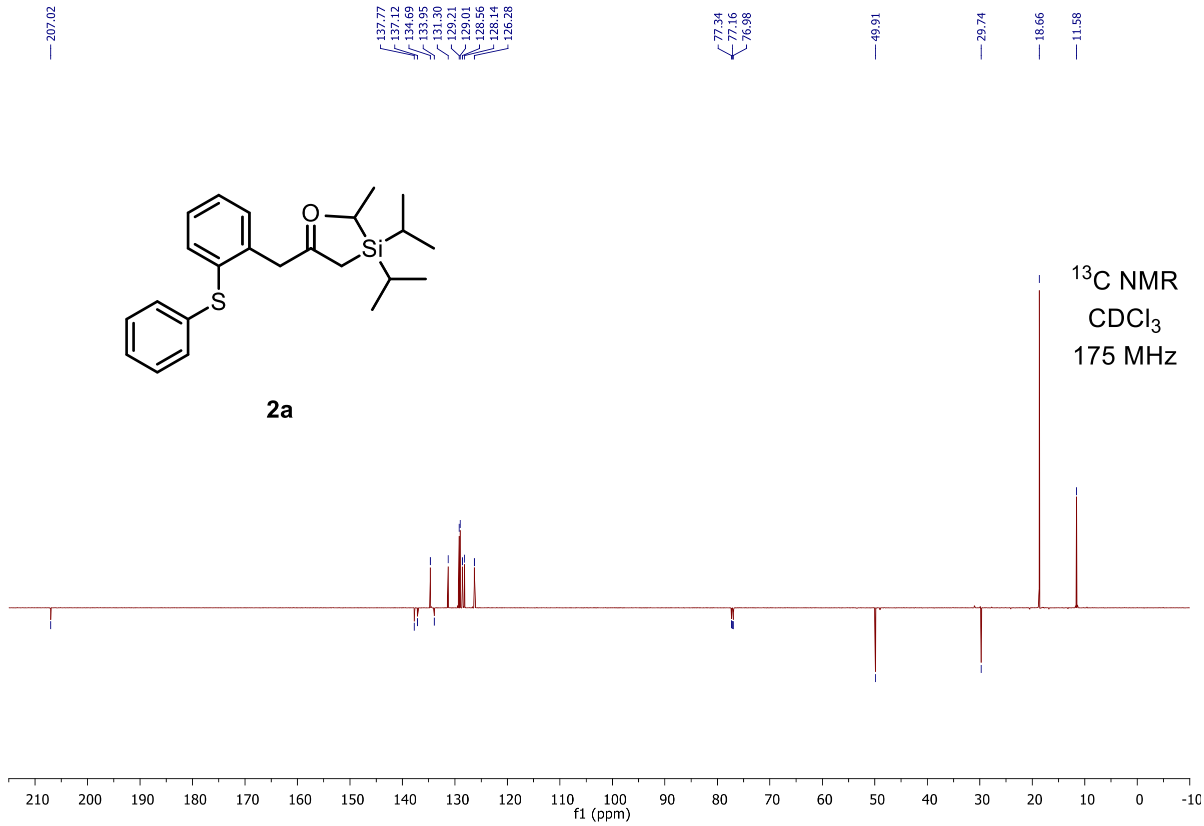

2a
^{13}C NMR
$CDCl_3$
175 MHz
207.02
137.77
137.12
134.69
133.95
131.30
129.21
129.01
128.56
128.14
126.28
77.34
77.16
76.98
49.91
29.74
18.66
11.58
f1 (ppm)

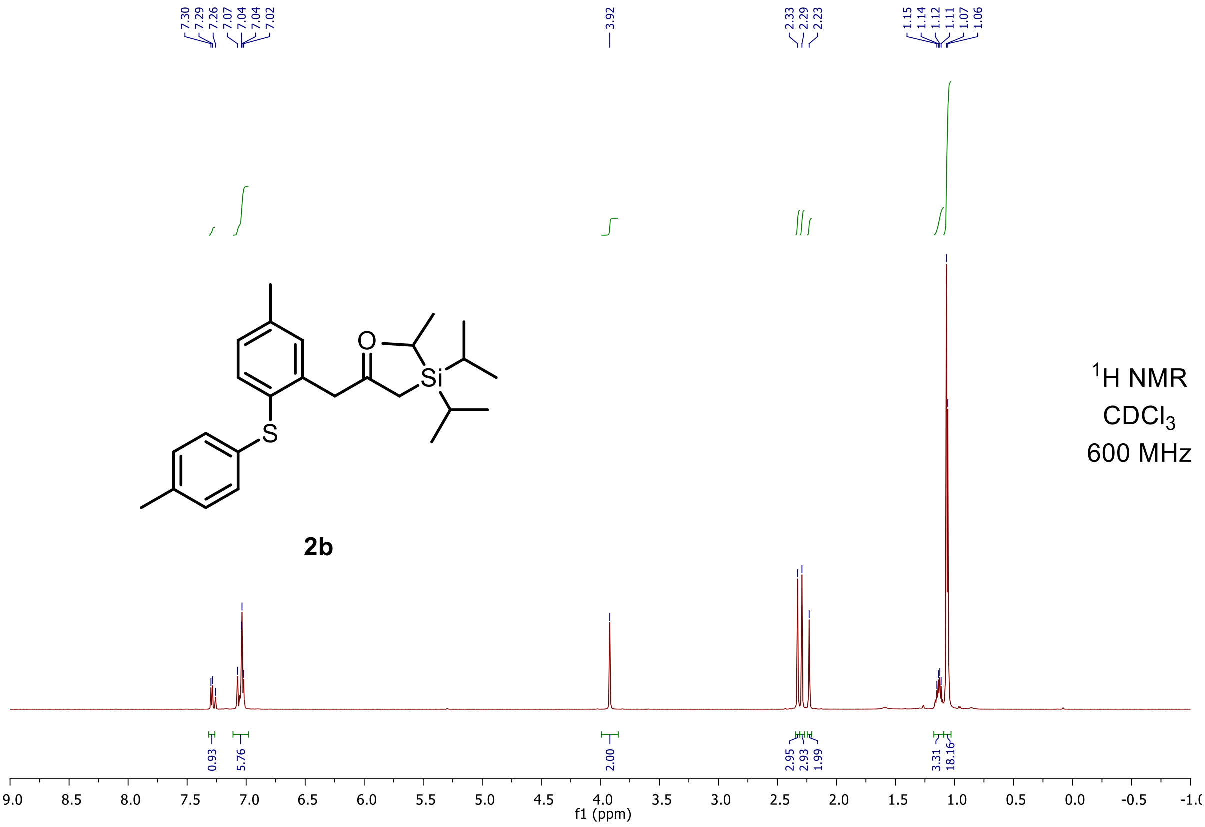
7.30
7.29
7.26
7.07
7.04
7.04
7.02
3.92
2.33
2.29
2.23
1.15
1.14
1.12
1.11
1.07
1.06
O
Si
S
2b
^{1}H NMR
$CDCl_3$
600 MHz
0.93
5.76
2.00
2.95
2.93
1.99
3.31
18.16
9.0 8.5 8.0 7.5 7.0 6.5 6.0 5.5 5.0 4.5 4.0 3.5 3.0 2.5 2.0 1.5 1.0 0.5 0.0 -0.5
f1 (ppm)

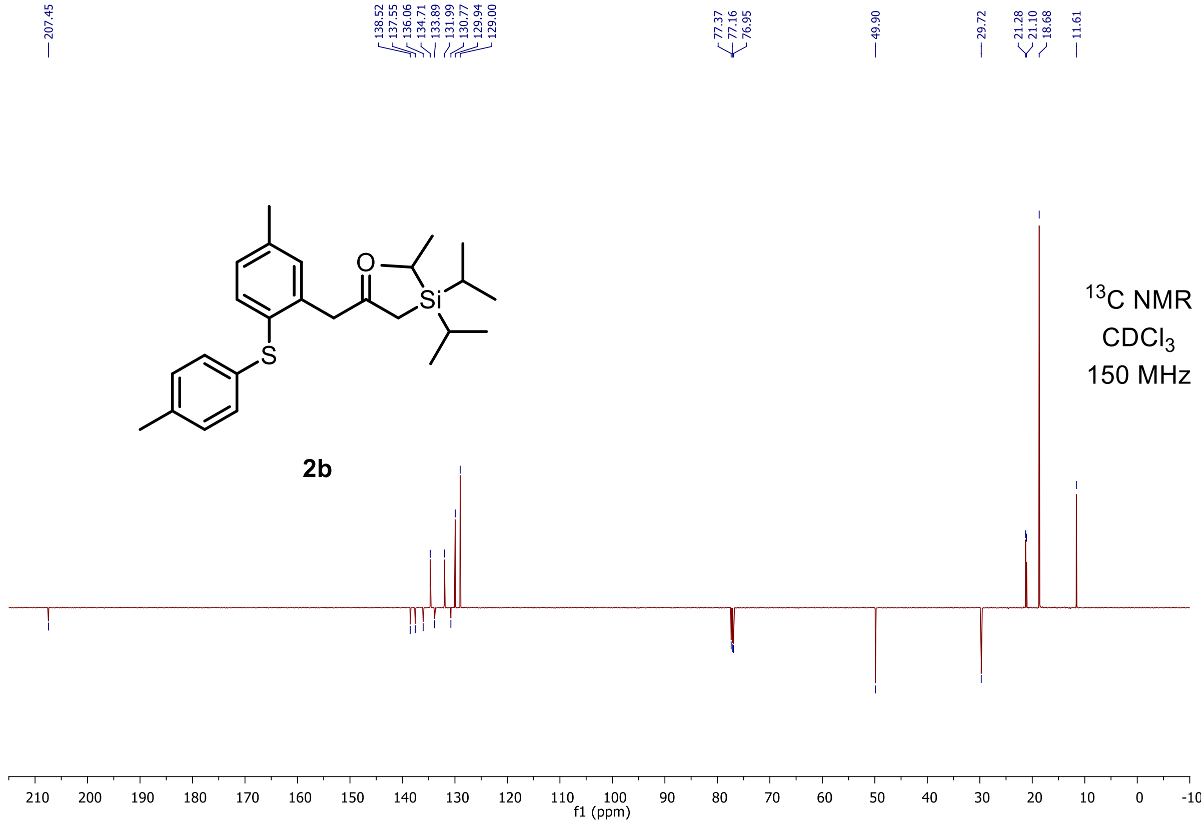
207.45
138.52
137.55
136.06
134.71
133.89
131.99
130.77
129.94
129.00
77.37
77.16
76.95
49.90
29.72
21.28
21.10
18.68
11.61
O
Si
S
2b
^{13}C NMR
$CDCl_3$
150 MHz
210 200 190 180 170 160 150 140 130 120 110 100 90 80 70 60 50 40 30 20 10 0
f1 (ppm)

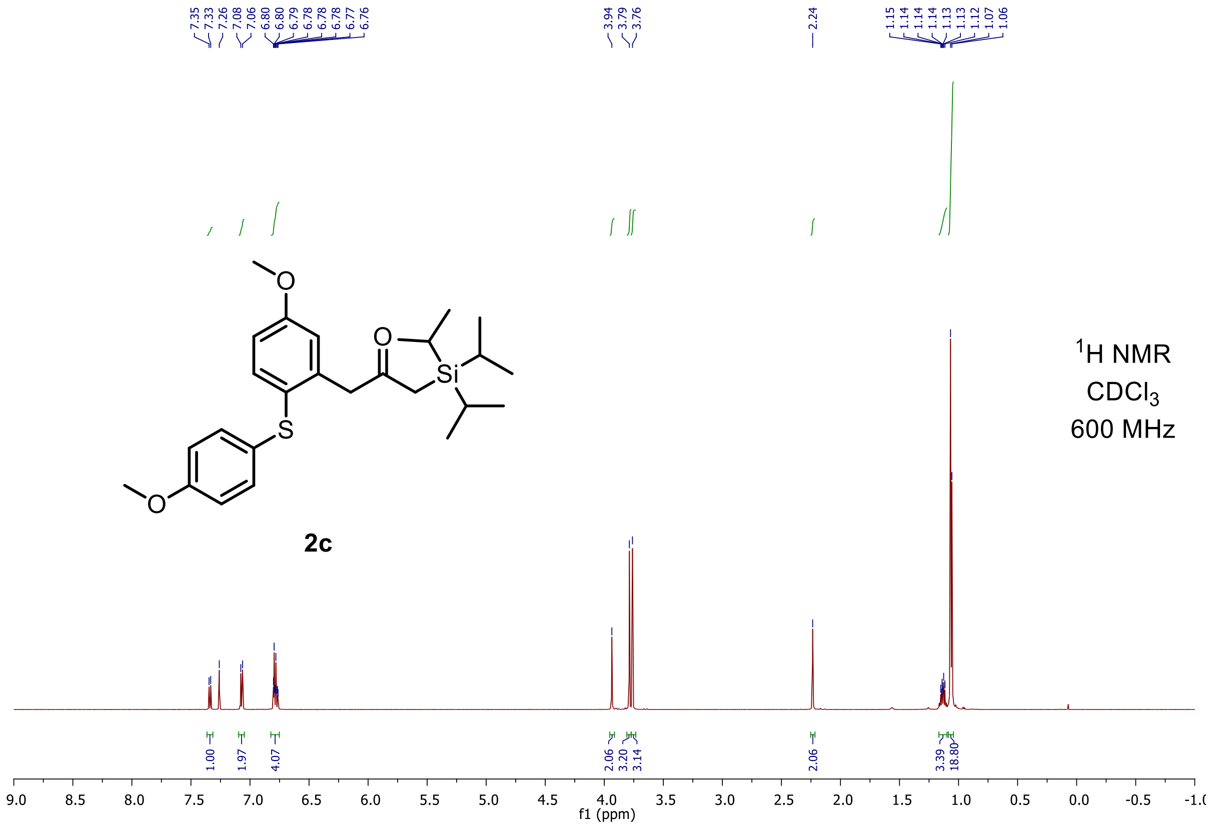

2c
1H NMR
CDCl3
600 MHz
f1 (ppm)

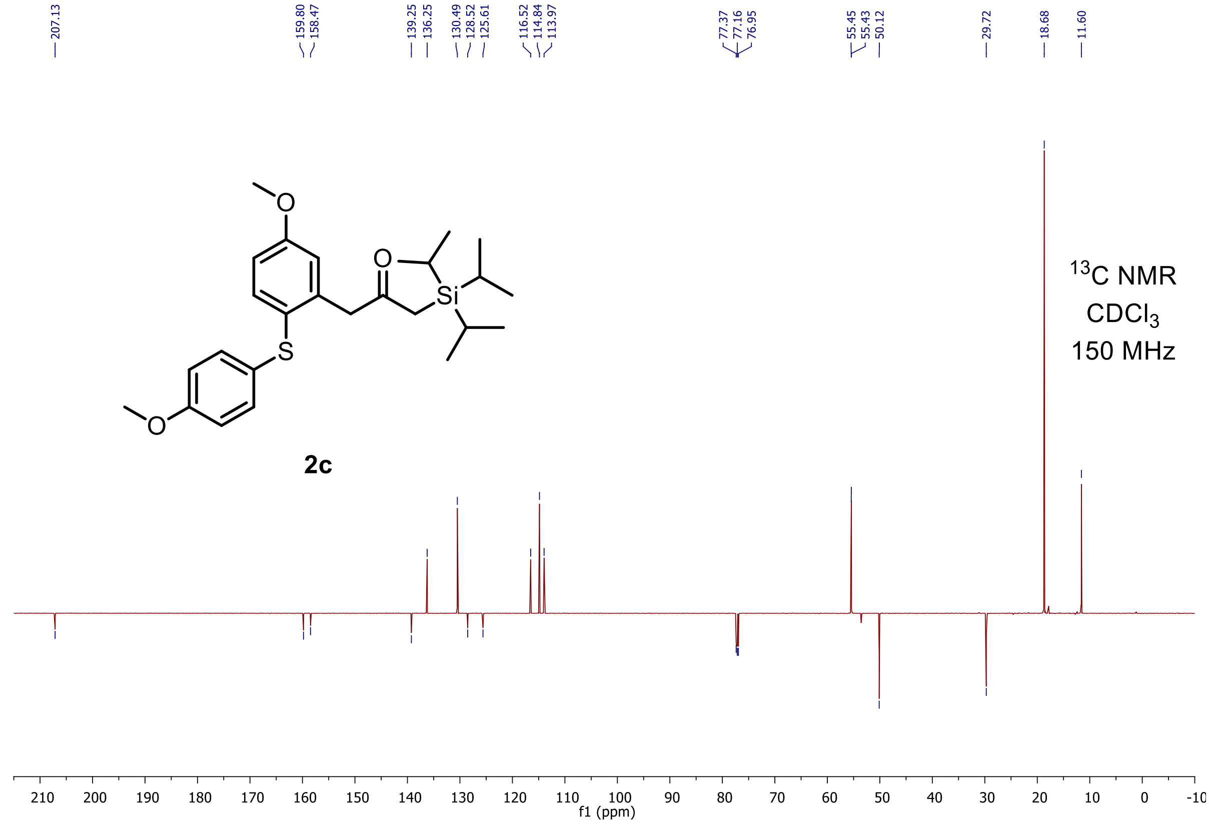

2c
13C NMR
CDCl3
150 MHz
f1 (ppm)

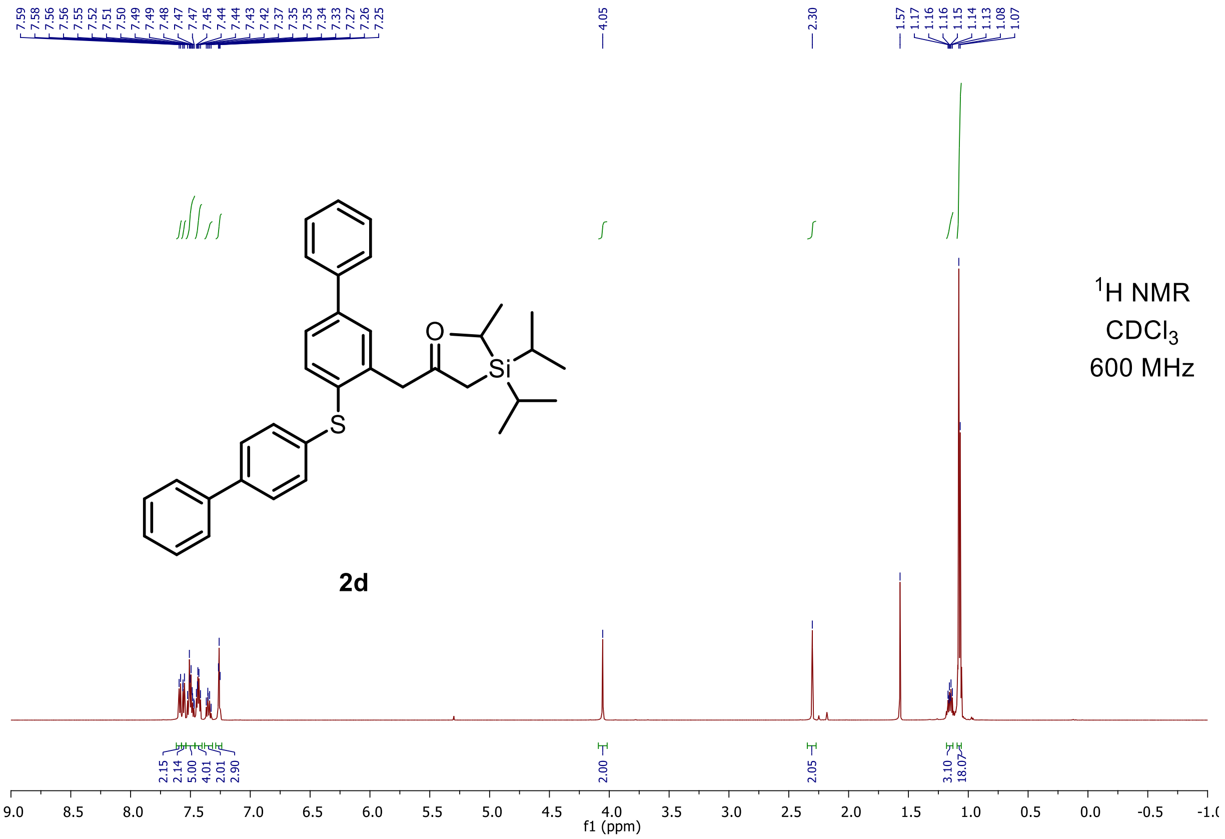

7.59 7.58 7.56 7.56 7.55 7.52 7.51 7.50 7.49 7.49 7.48 7.47 7.47 7.45 7.44 7.44 7.43 7.42 7.37 7.35 7.35 7.34 7.33 7.27 7.26 7.25
4.05
2.30
1.57 1.17 1.16 1.16 1.15 1.14 1.13 1.08 1.07
O
Si
S
2d
^{1}H NMR
$CDCl_3$
600 MHz
2.15 2.14 5.00 4.01 2.01 2.90
2.00
2.05
3.10 18.07
f1 (ppm)

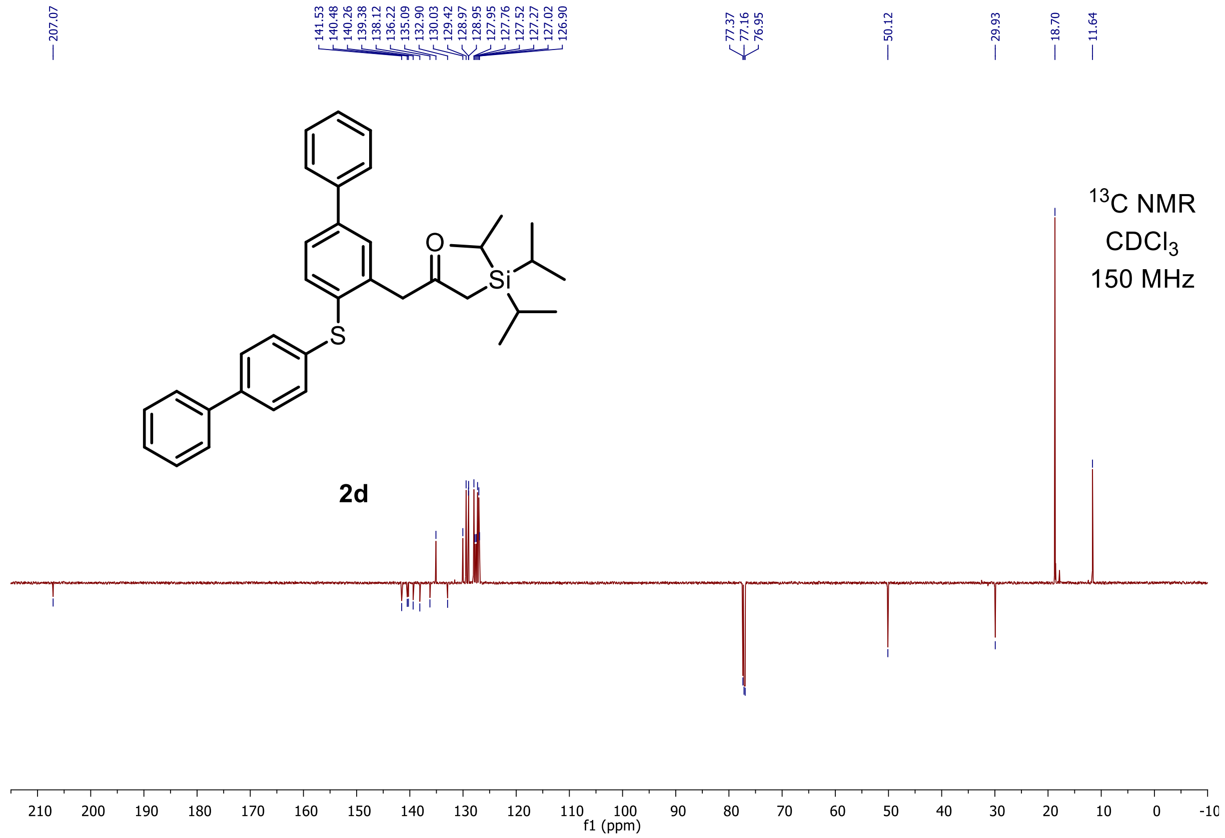

207.07
141.53 140.48 140.26 139.38 138.12 136.22 135.09 132.90 130.03 129.42 128.97 128.95 127.95 127.76 127.52 127.27 127.02 126.90
77.37 77.16 76.95
50.12
29.93
18.70
11.64
O
Si
S
2d
^{13}C NMR
$CDCl_3$
150 MHz
f1 (ppm)

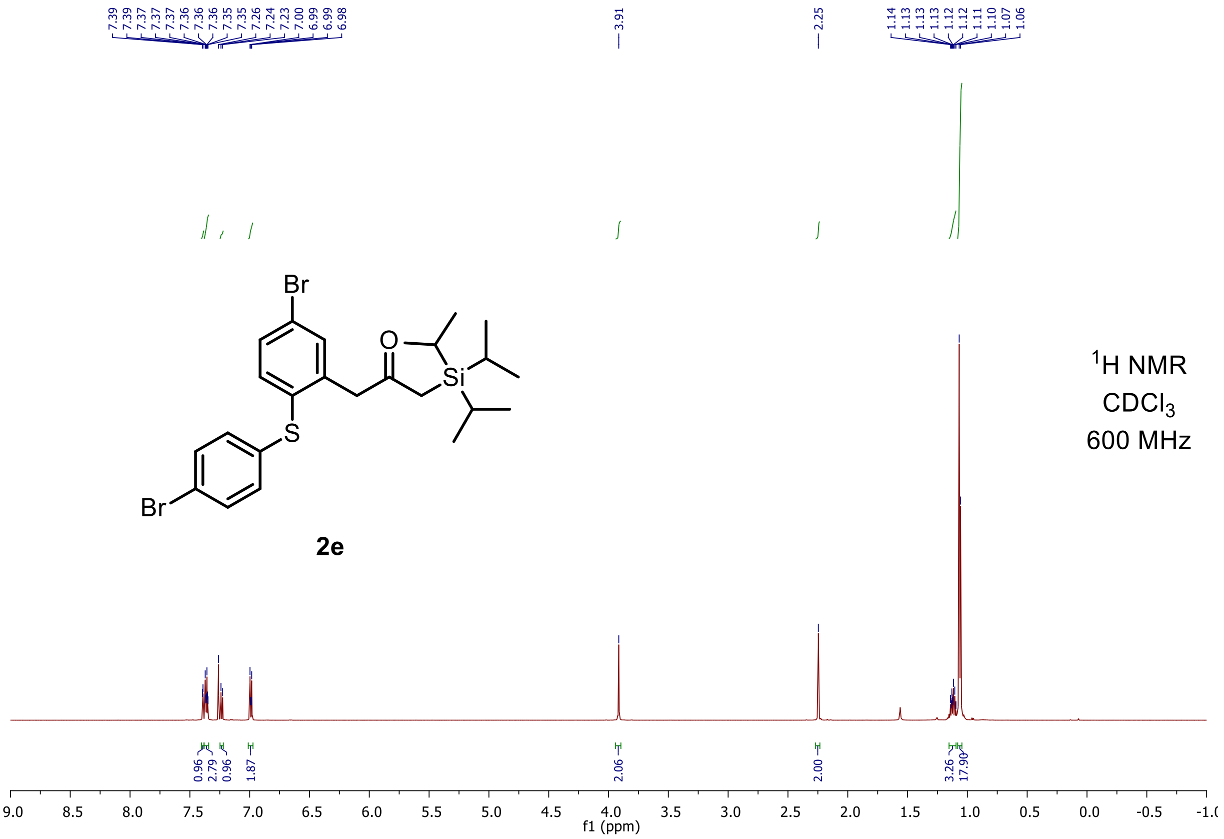
7.39
7.39
7.37
7.37
7.37
7.36
7.36
7.36
7.35
7.35
7.26
7.24
7.23
7.00
6.99
6.99
6.98
3.91
2.25
1.14
1.13
1.13
1.13
1.12
1.12
1.11
1.10
1.07
1.06
Br
O
Si
S
Br
2e
1H NMR
CDCl3
600 MHz
0.96
2.79
0.96
1.87
2.06
2.00
3.26
17.90
f1 (ppm)

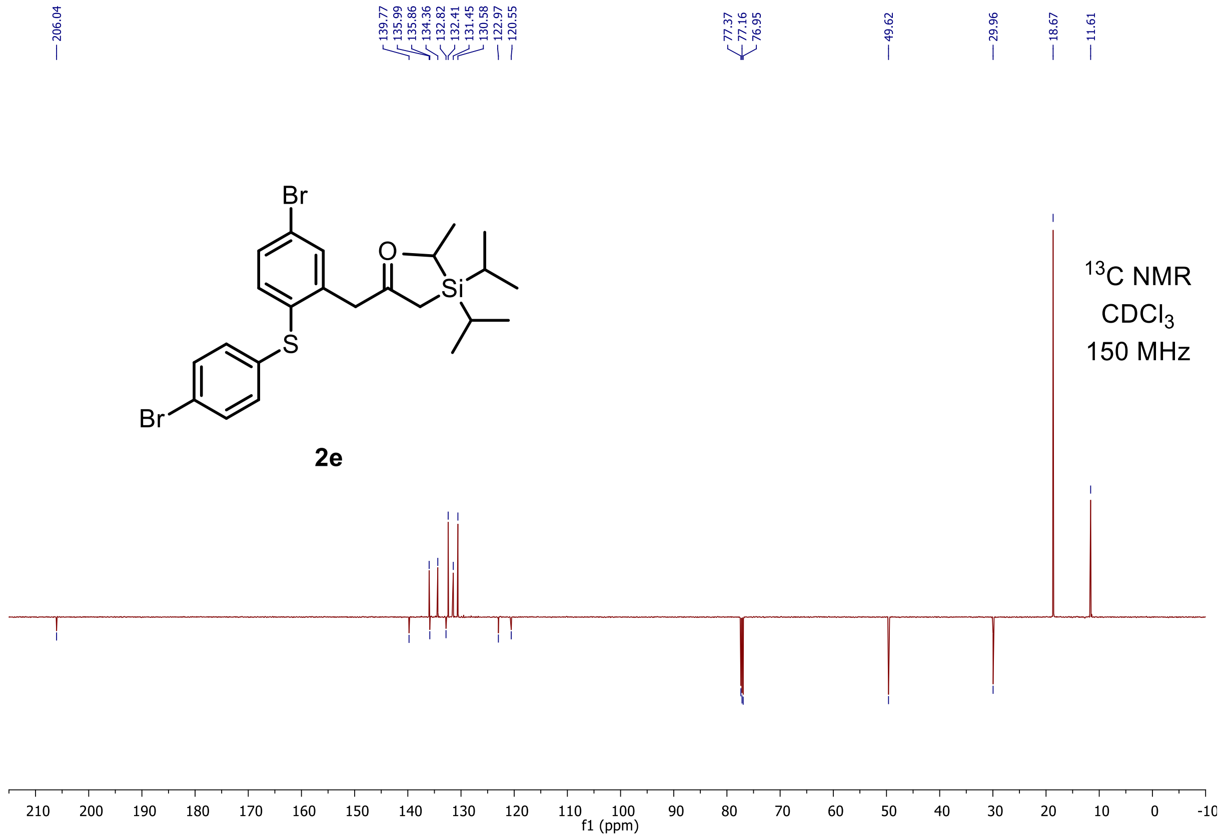
206.04
139.77
135.99
135.86
134.36
132.82
132.41
131.45
130.58
122.97
120.55
77.37
77.16
76.95
49.62
29.96
18.67
11.61
Br
O
Si
S
Br
2e
13C NMR
CDCl3
150 MHz
f1 (ppm)

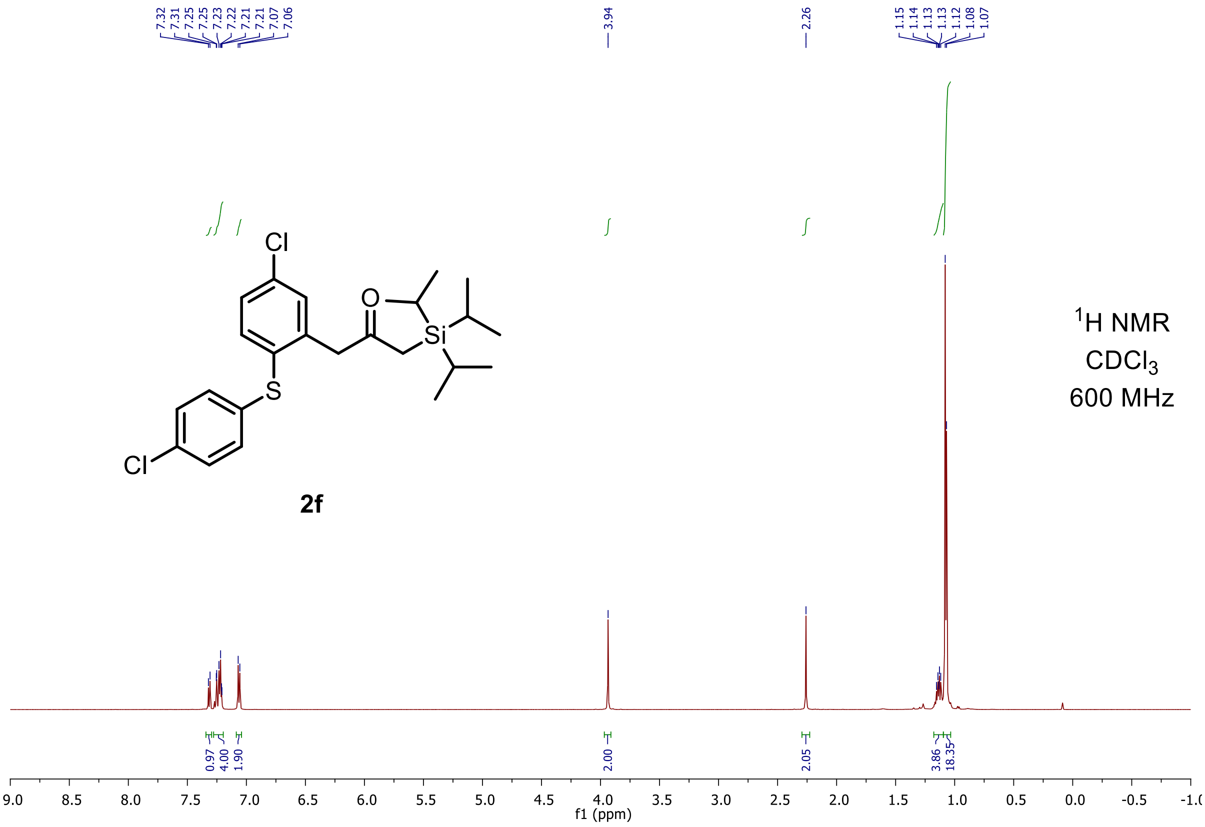
7.32
7.31
7.25
7.25
7.23
7.22
7.21
7.21
7.07
7.06
3.94
2.26
1.15
1.14
1.13
1.13
1.12
1.08
1.07
Cl
O
Si
S
Cl
2f
^{1}H NMR
CDCl$_3$
600 MHz
0.97
4.00
1.90
2.00
2.05
3.86
18.35
f1 (ppm)

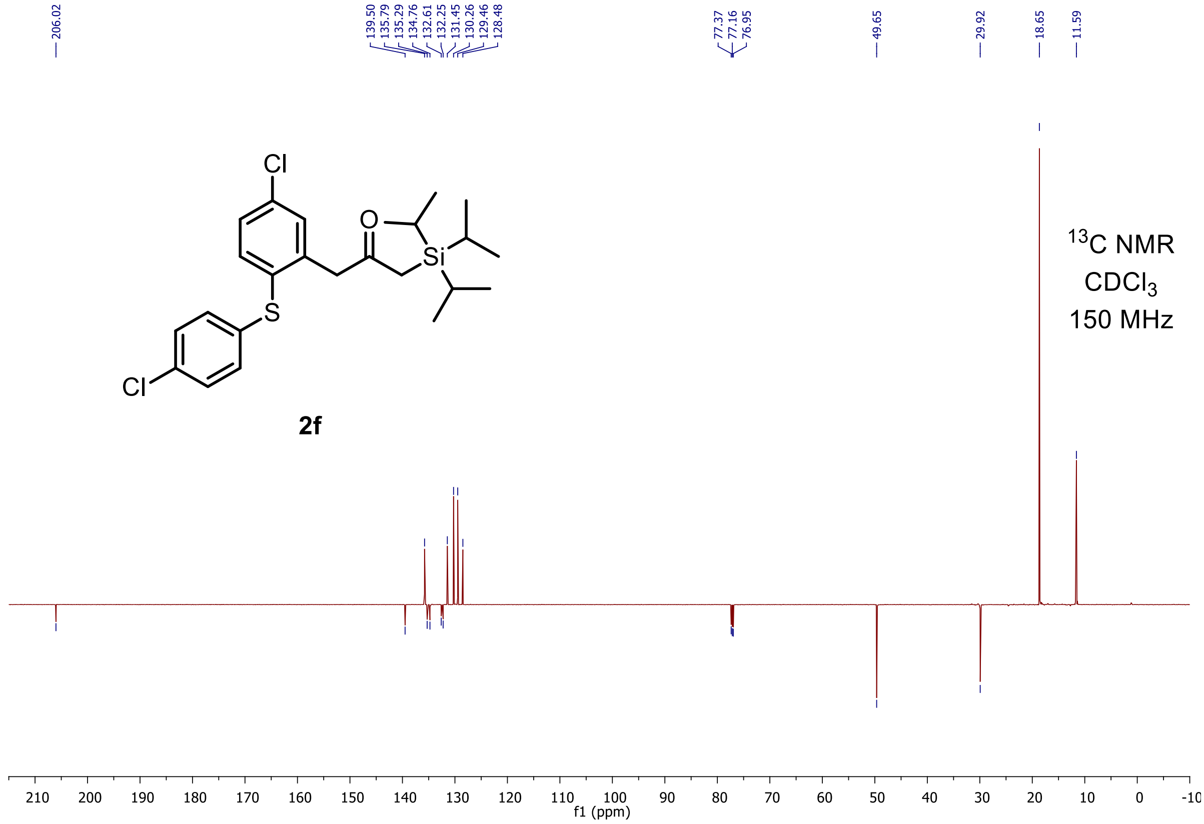
206.02
139.50
135.79
135.29
134.76
132.61
132.25
131.45
130.26
129.46
128.48
77.37
77.16
76.95
49.65
29.92
18.65
11.59
Cl
O
Si
S
Cl
2f
^{13}C NMR
CDCl$_3$
150 MHz
f1 (ppm)

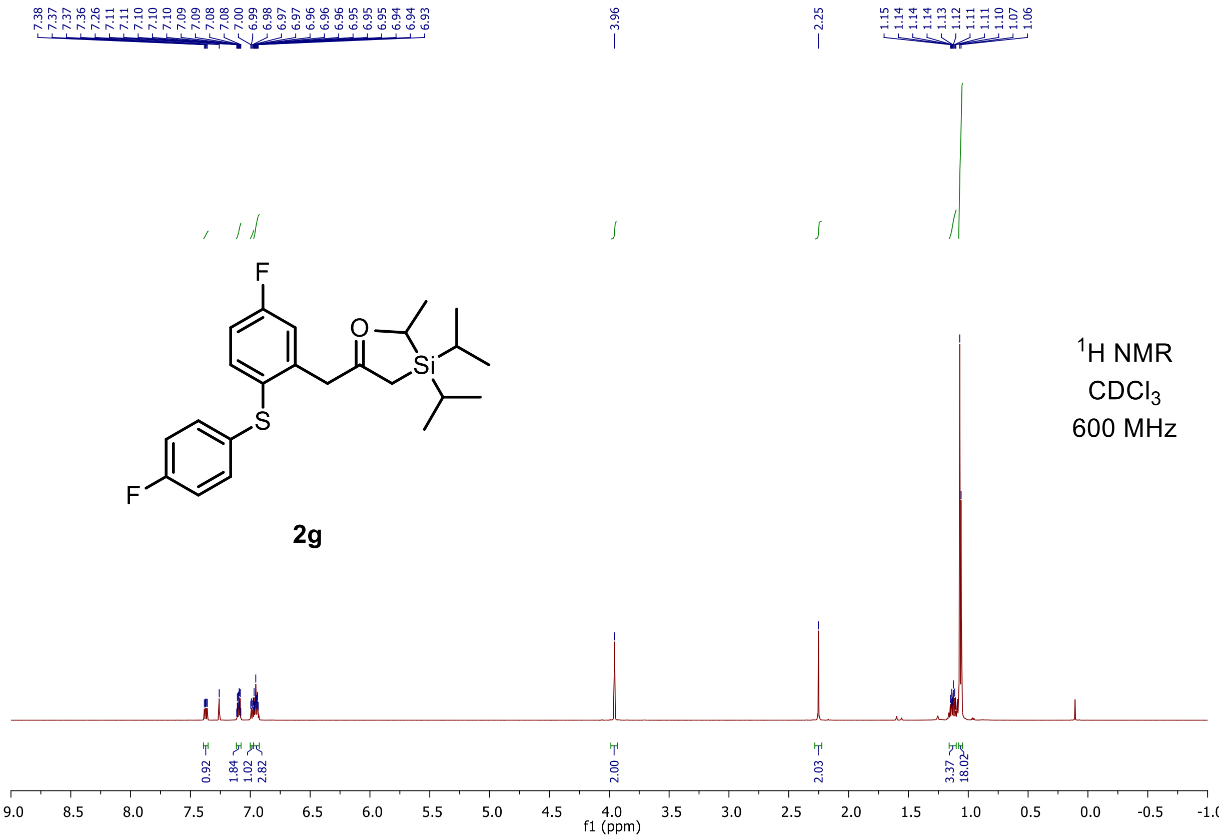
2g
^{1}H NMR
$CDCl_3$
600 MHz
f1 (ppm)

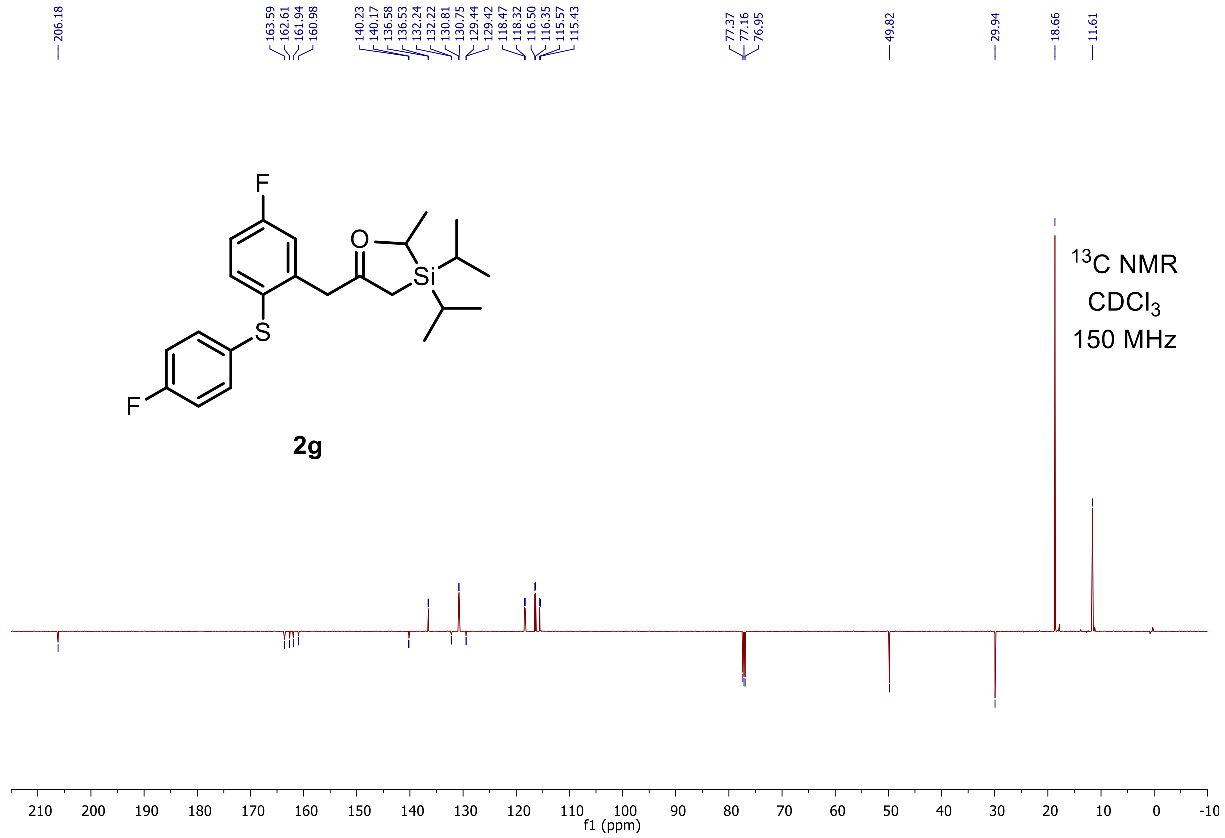
2g
^{13}C NMR
$CDCl_3$
150 MHz
f1 (ppm)

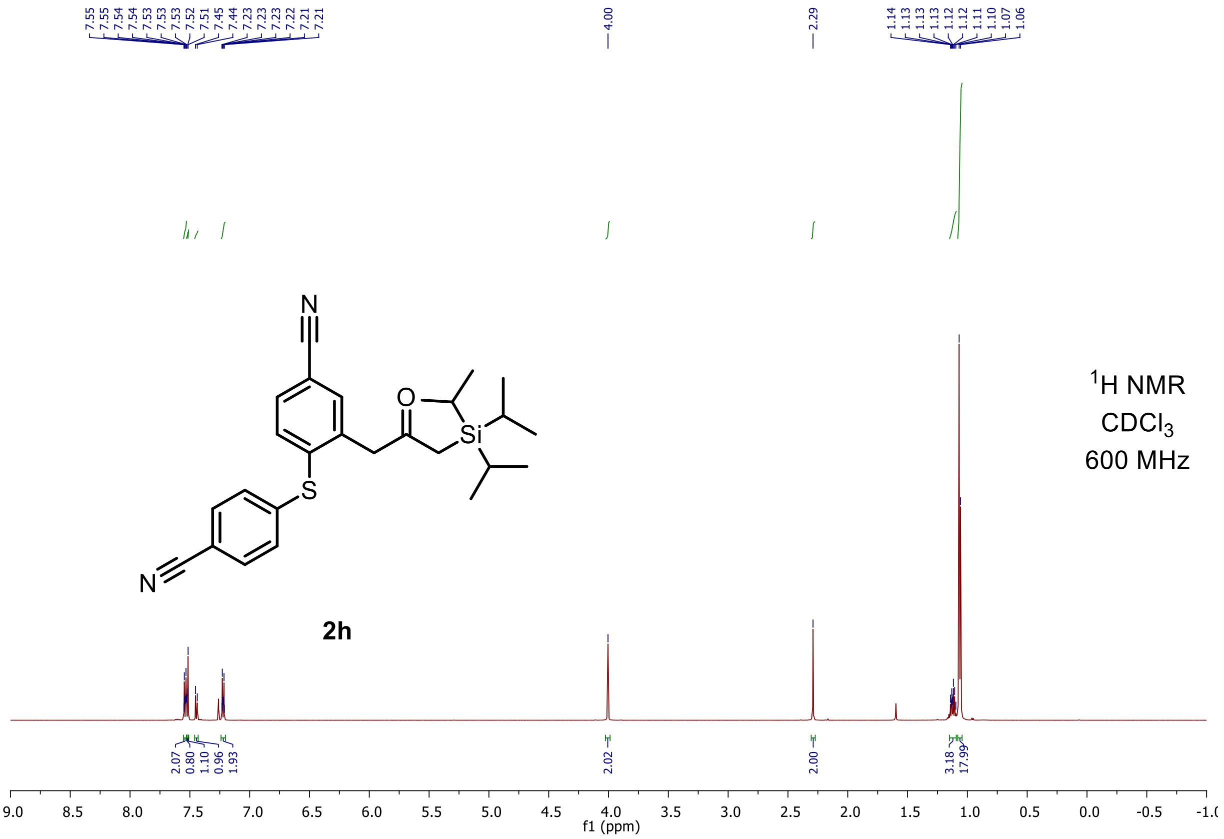

2h
1H NMR
CDCl3
600 MHz
f1 (ppm)

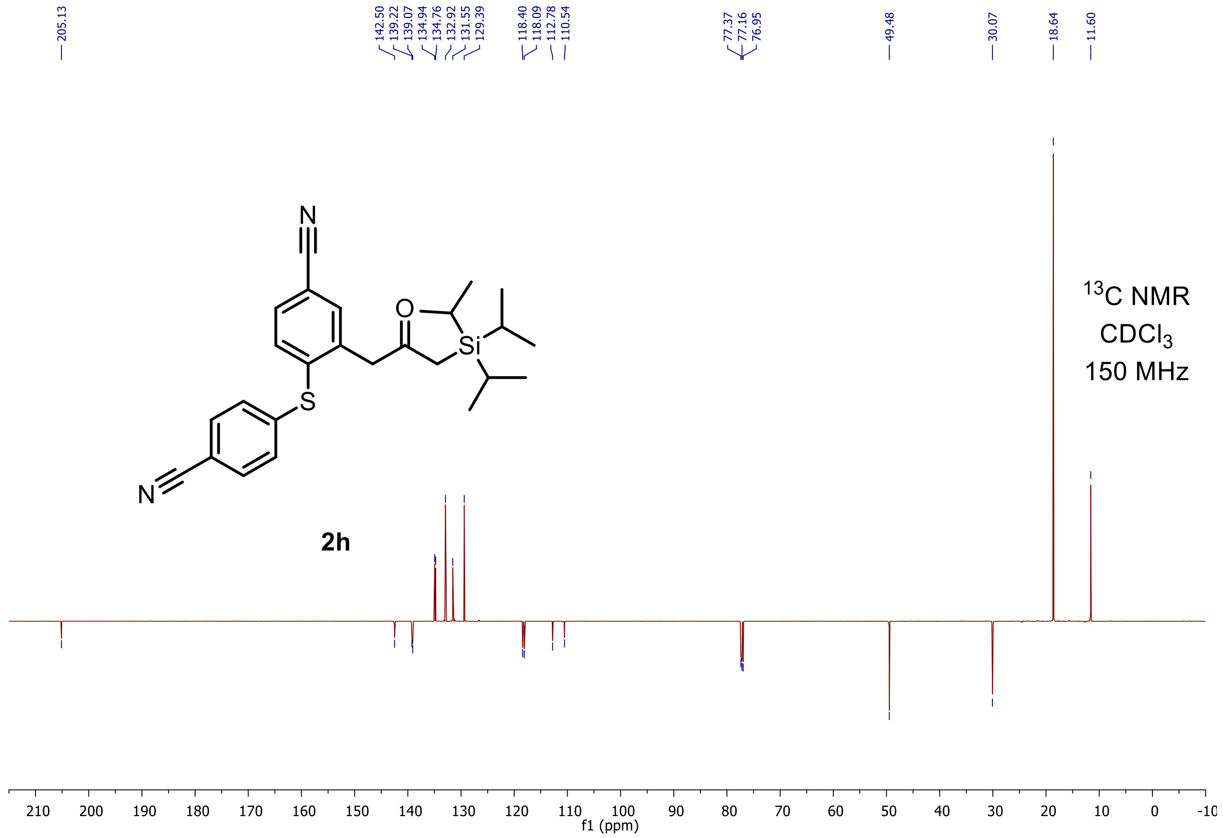

2h
13C NMR
CDCl3
150 MHz
f1 (ppm)

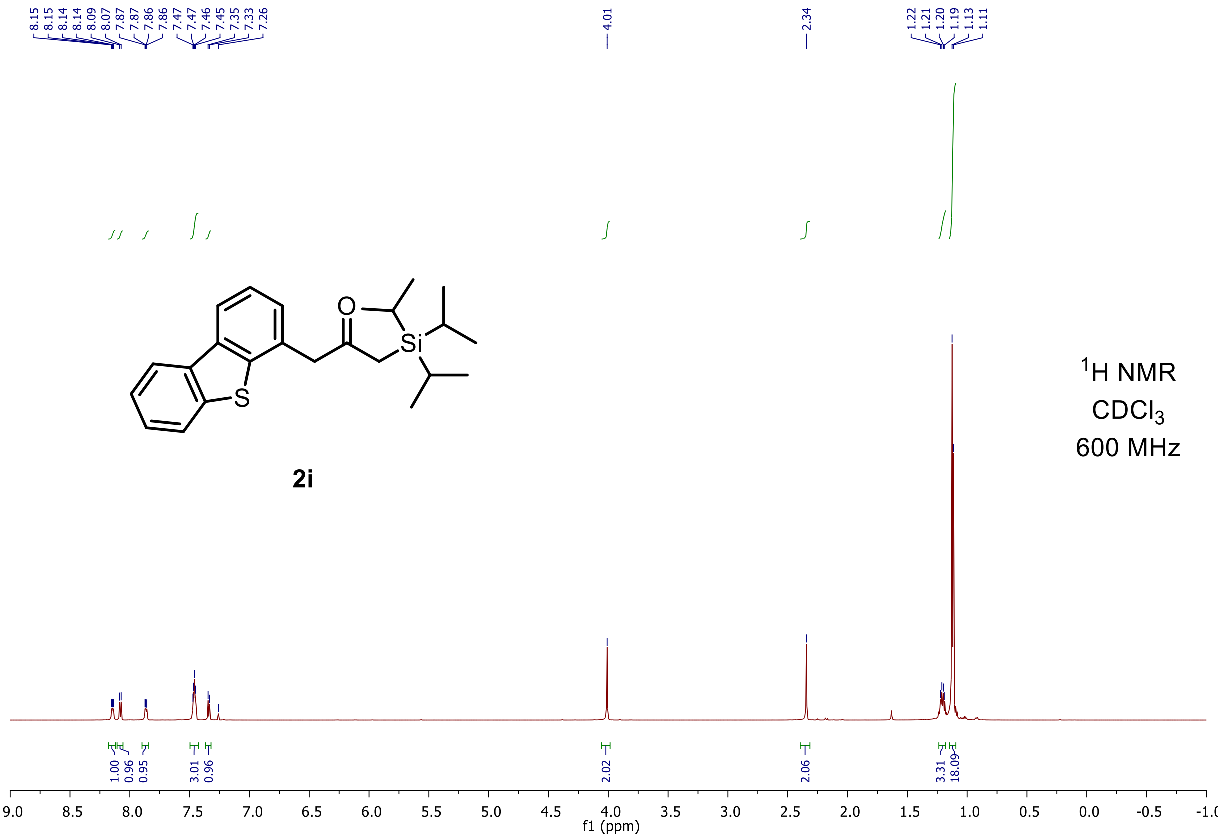
8.15
8.15
8.14
8.14
8.09
8.07
7.87
7.87
7.86
7.86
7.47
7.47
7.46
7.45
7.35
7.33
7.26
4.01
2.34
1.22
1.21
1.20
1.19
1.13
1.11
O
Si
S
2i
^{1}H NMR
$CDCl_3$
600 MHz
1.00
0.96
0.95
3.01
0.96
2.02
2.06
3.31
18.09
f1 (ppm)

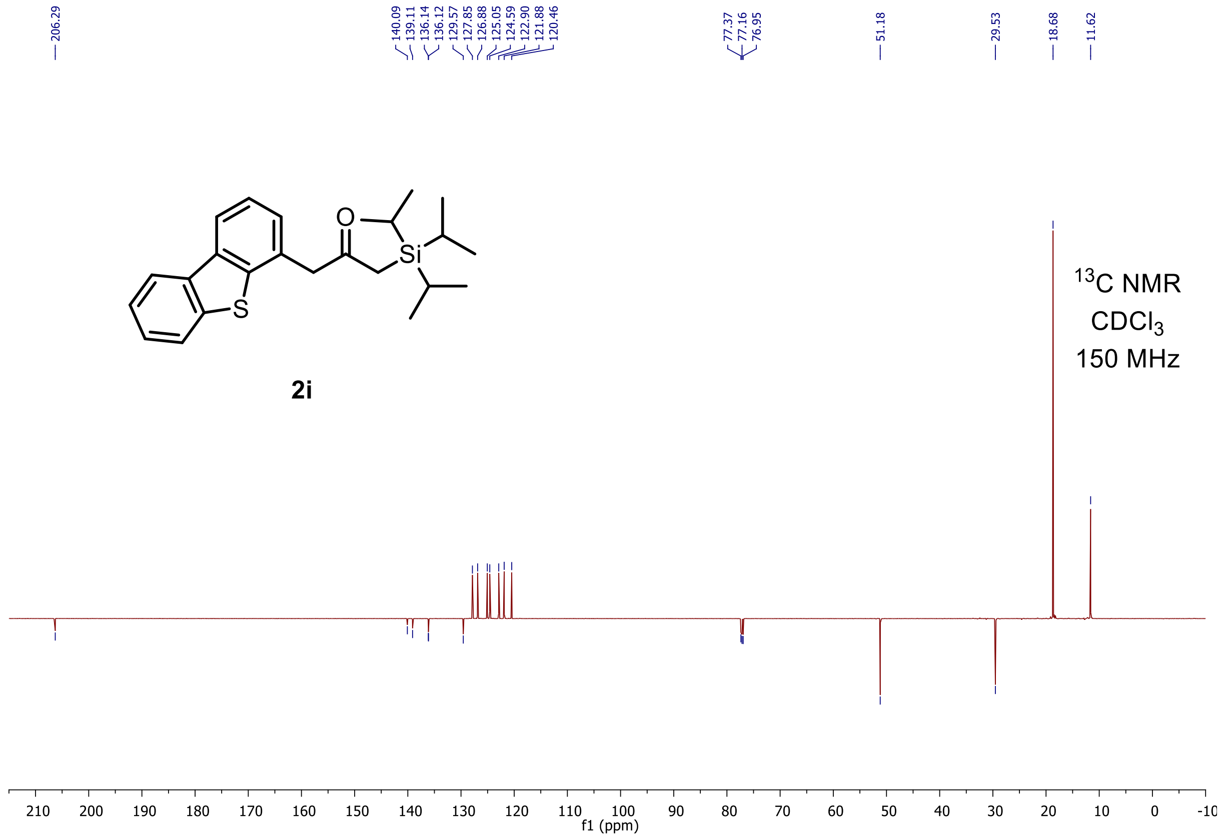
206.29
140.09
139.11
136.14
136.12
129.57
127.85
126.88
125.05
124.59
122.90
121.88
120.46
77.37
77.16
76.95
51.18
29.53
18.68
11.62
O
Si
S
2i
^{13}C NMR
$CDCl_3$
150 MHz
f1 (ppm)

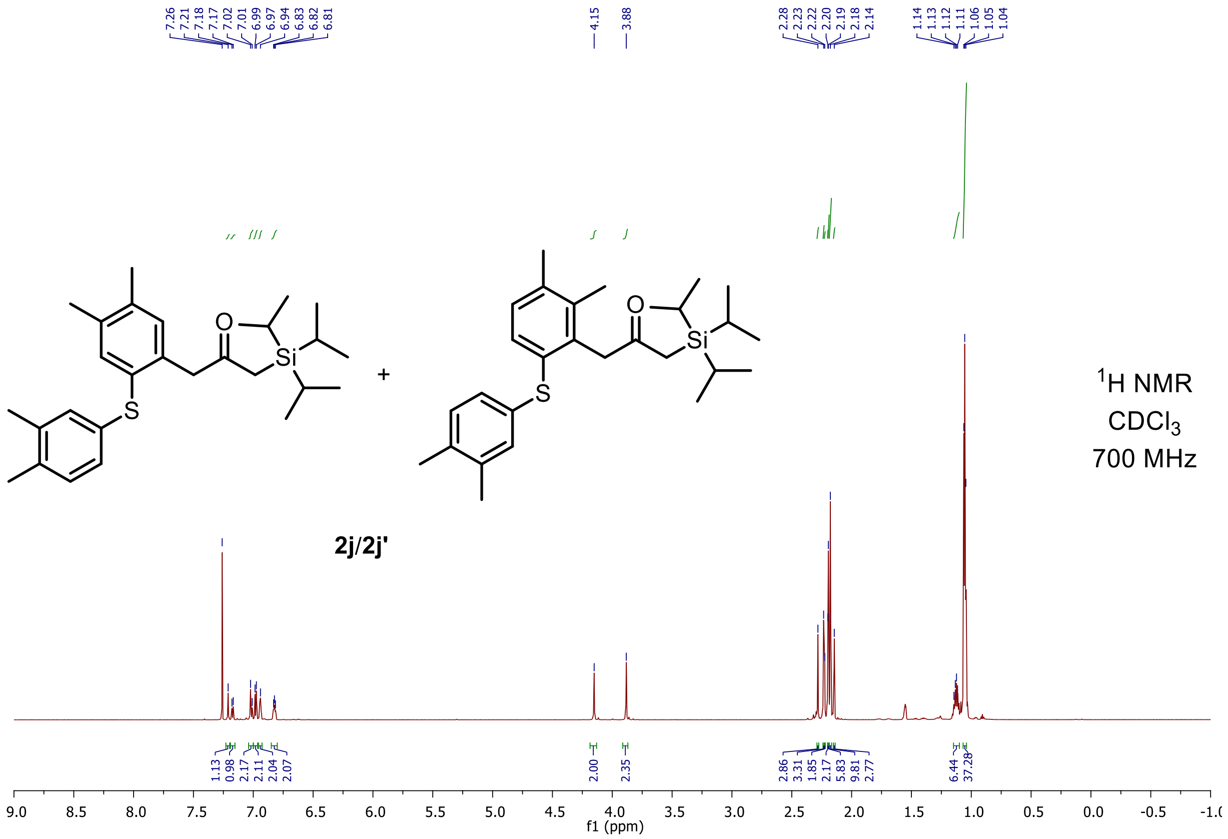

2j/2j'
^{1}H NMR
$CDCl_3$
700 MHz
f1 (ppm)

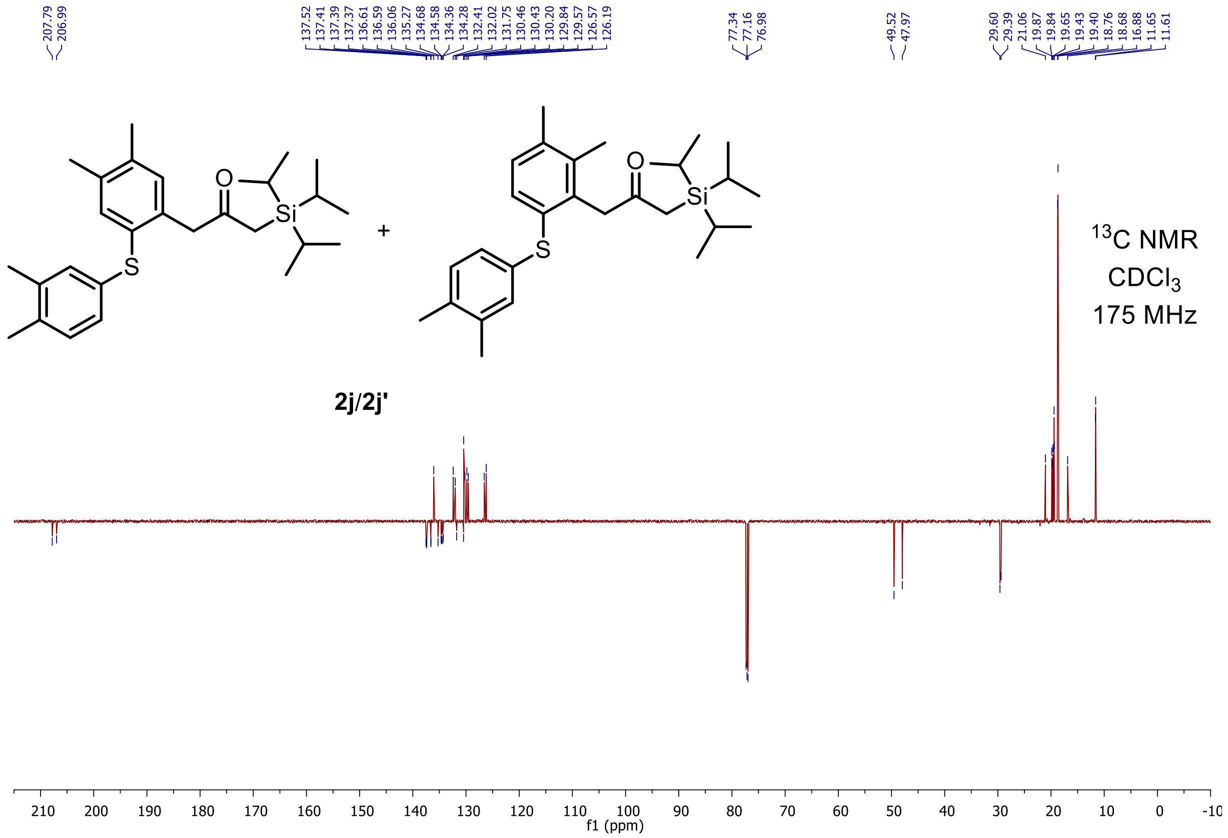

2j/2j'
^{13}C NMR
$CDCl_3$
175 MHz
f1 (ppm)

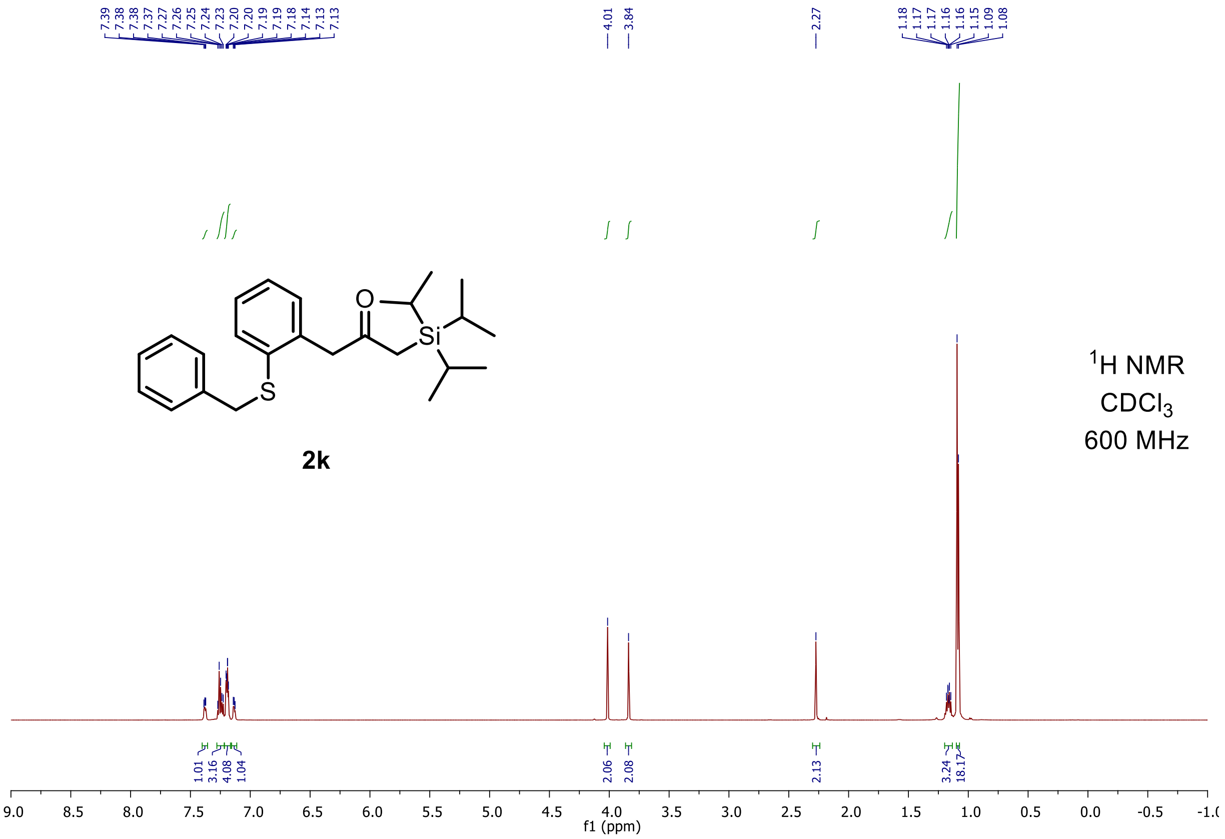
7.39
7.38
7.38
7.37
7.27
7.26
7.25
7.24
7.23
7.20
7.20
7.19
7.19
7.18
7.14
7.13
7.13
4.01
3.84
2.27
1.18
1.17
1.17
1.16
1.16
1.15
1.09
1.08
O
Si
S
2k
1H NMR
CDCl3
600 MHz
1.01
3.16
4.08
1.04
2.06
2.08
2.13
3.24
18.17
f1 (ppm)

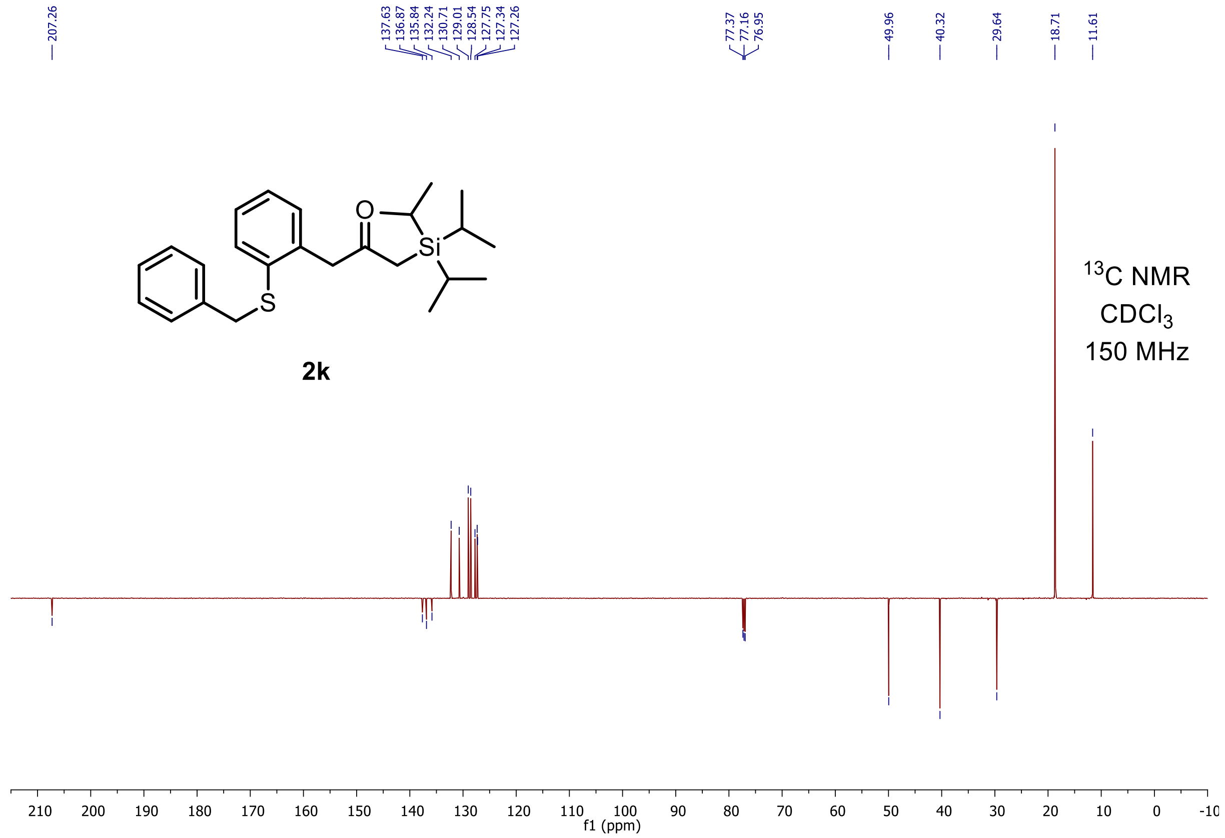
207.26
137.63
136.87
135.84
132.24
130.71
129.01
128.54
127.75
127.34
127.26
77.37
77.16
76.95
49.96
40.32
29.64
18.71
11.61
O
Si
S
2k
13C NMR
CDCl3
150 MHz
f1 (ppm)

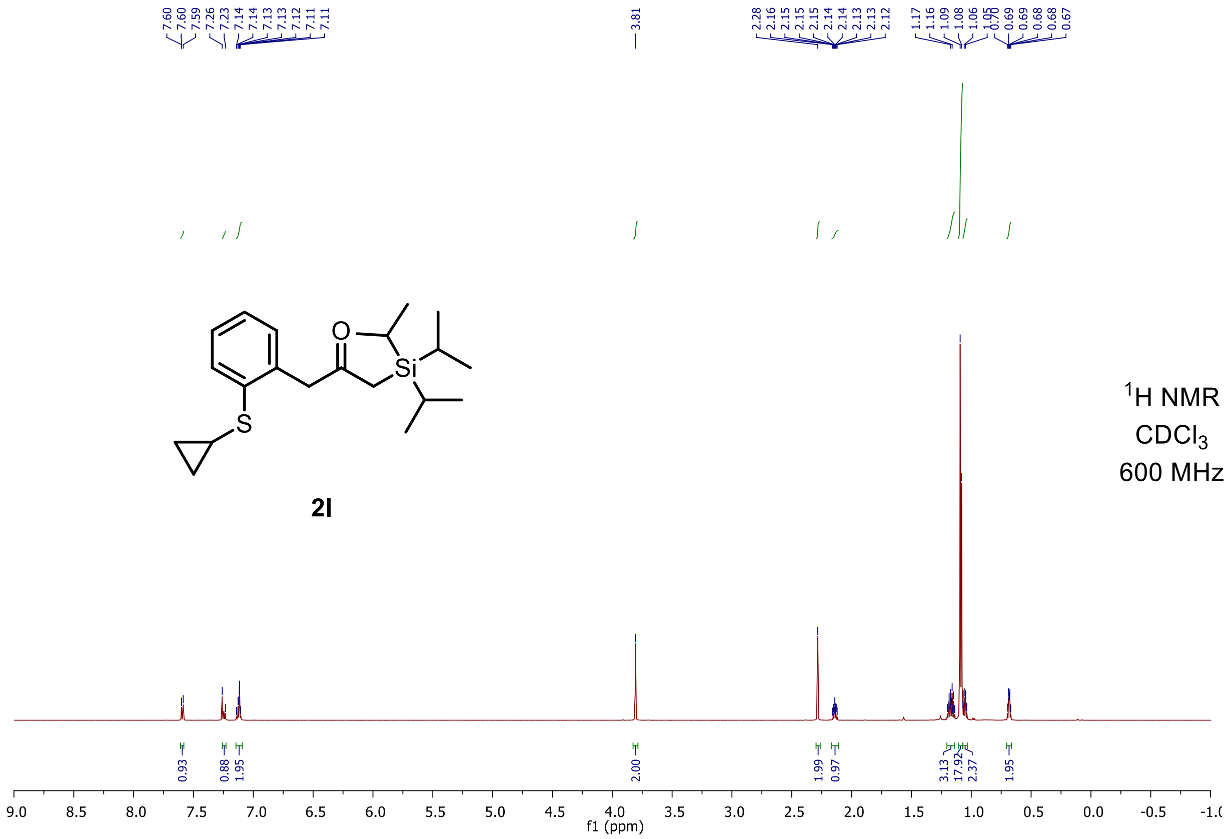
2l
1H NMR
CDCl3
600 MHz
f1 (ppm)

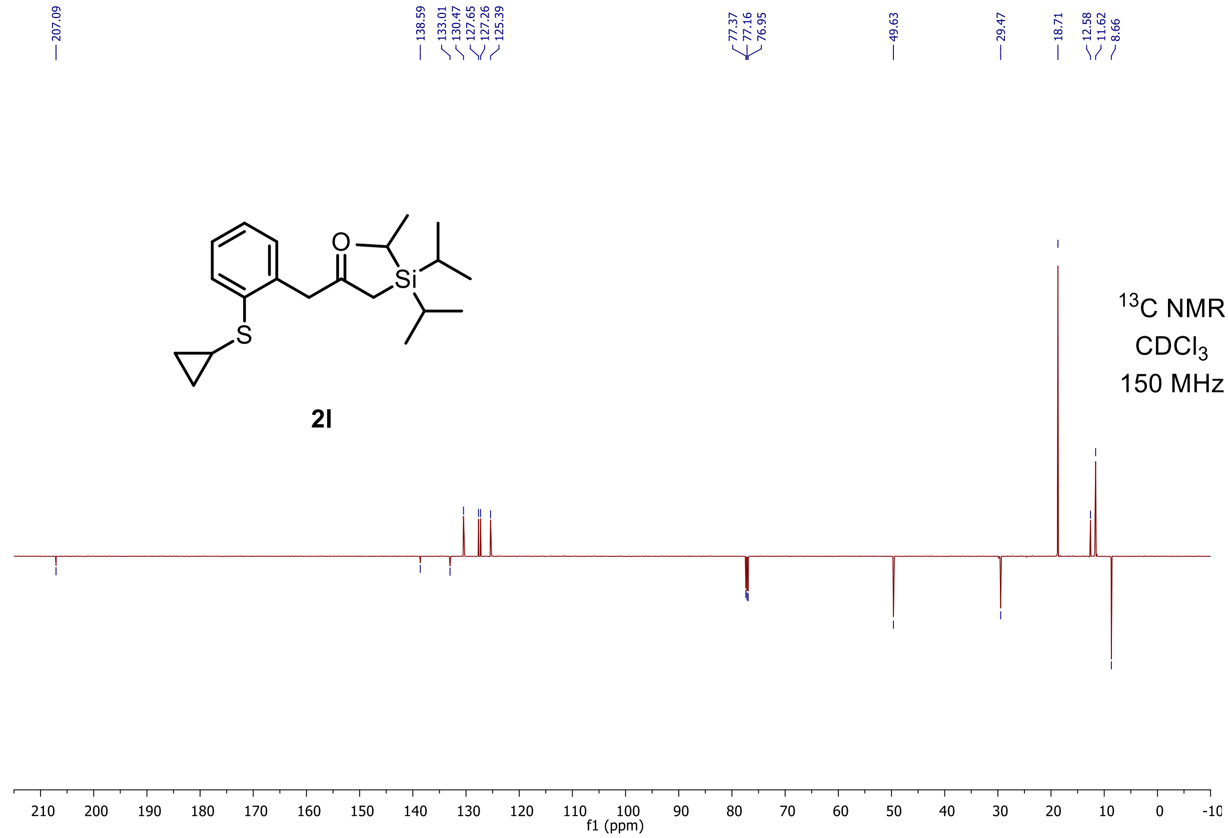
2l
13C NMR
CDCl3
150 MHz
f1 (ppm)

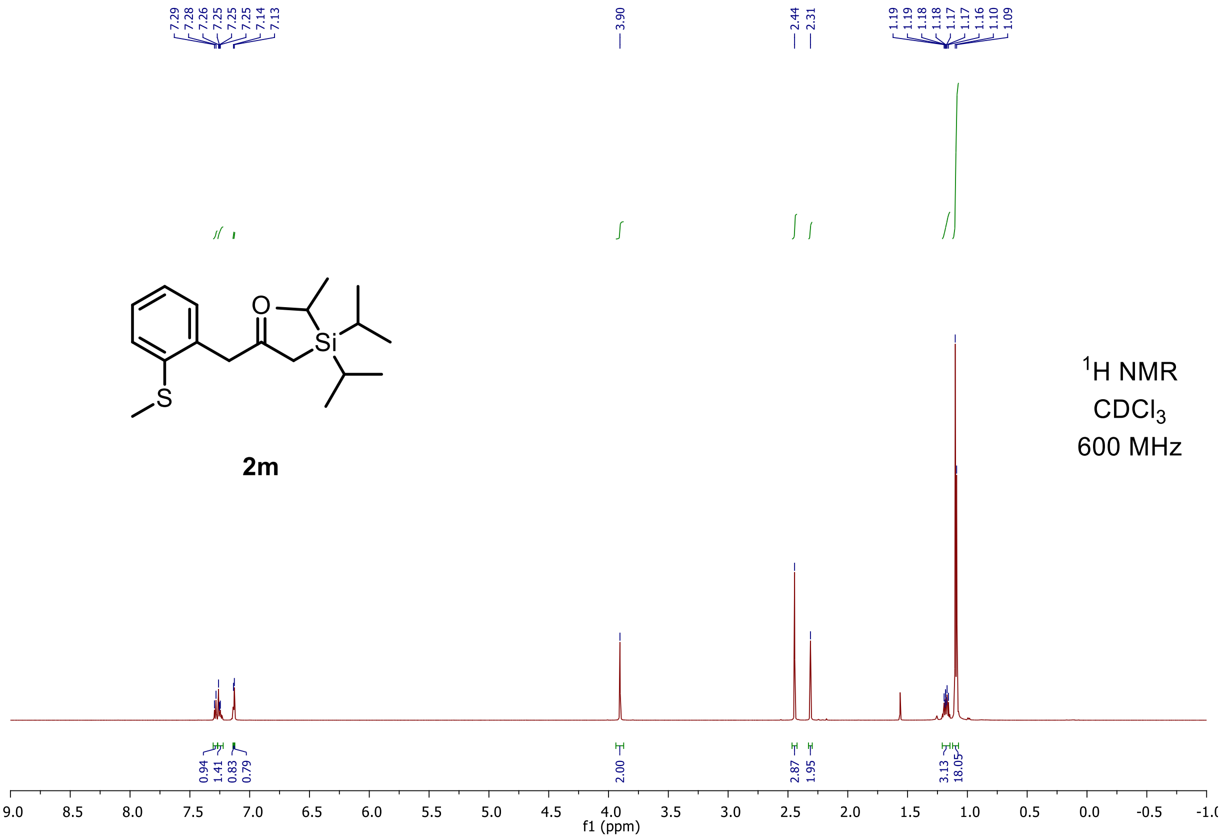
7.29
7.28
7.26
7.25
7.25
7.25
7.14
7.13
3.90
2.44
2.31
1.19
1.19
1.18
1.18
1.17
1.17
1.16
1.10
1.09
O
Si
S
2m
^{1}H NMR
$CDCl_3$
600 MHz
0.94
1.41
0.83
0.79
2.00
2.87
1.95
3.13
18.05
f1 (ppm)

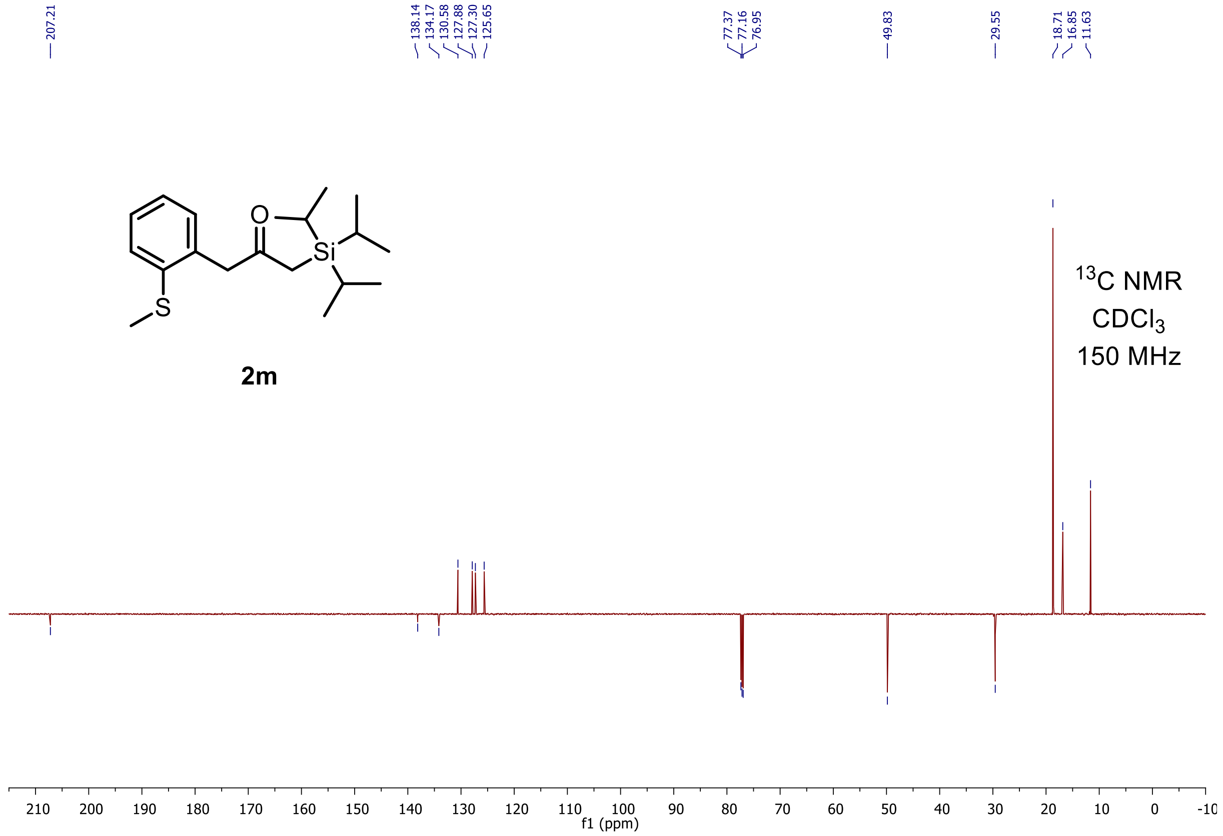
207.21
138.14
134.17
130.58
127.88
127.30
125.65
77.37
77.16
76.95
49.83
29.55
18.71
16.85
11.63
O
Si
S
2m
^{13}C NMR
$CDCl_3$
150 MHz
f1 (ppm)

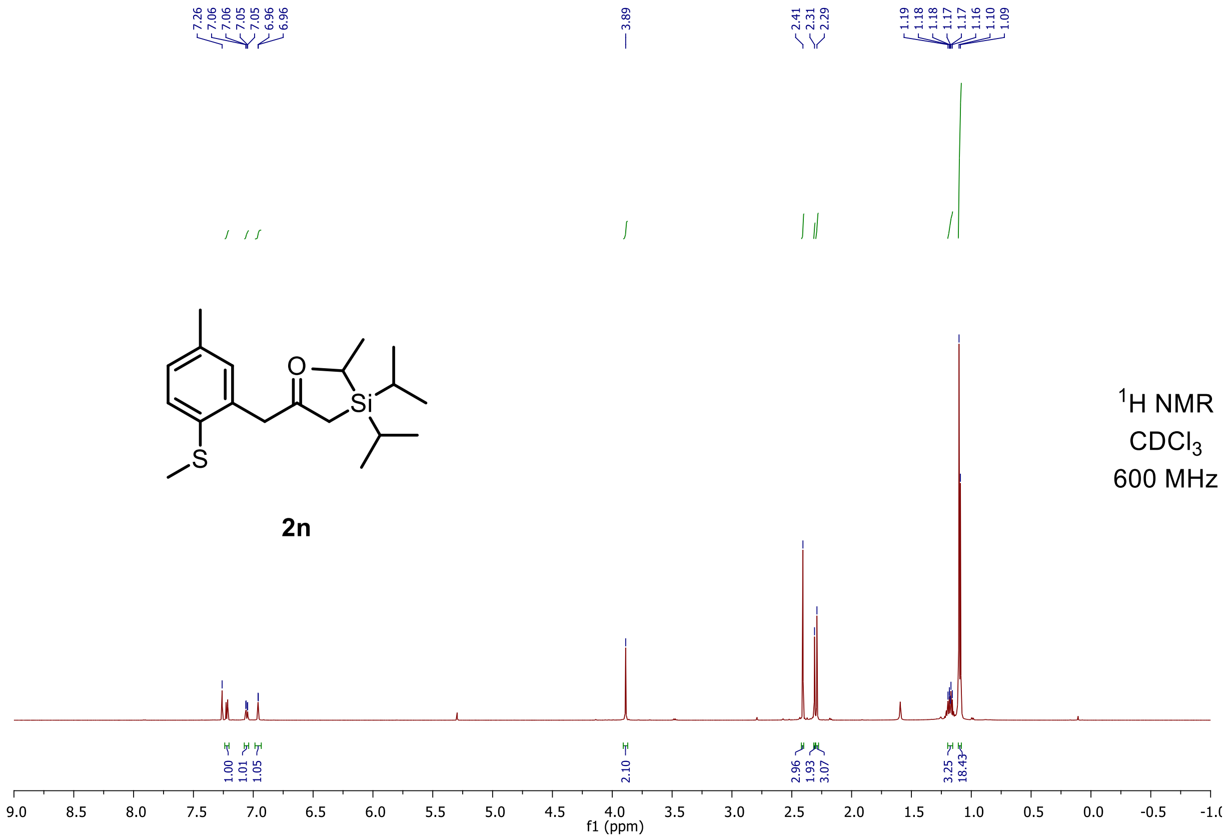
7.26
7.06
7.06
7.05
7.05
6.96
6.96
3.89
2.41
2.31
2.29
1.19
1.18
1.18
1.17
1.17
1.16
1.10
1.09
O
Si
S
2n
1H NMR
CDCl3
600 MHz
1.00
1.01
1.05
2.10
2.96
1.93
3.07
3.25
18.43
f1 (ppm)

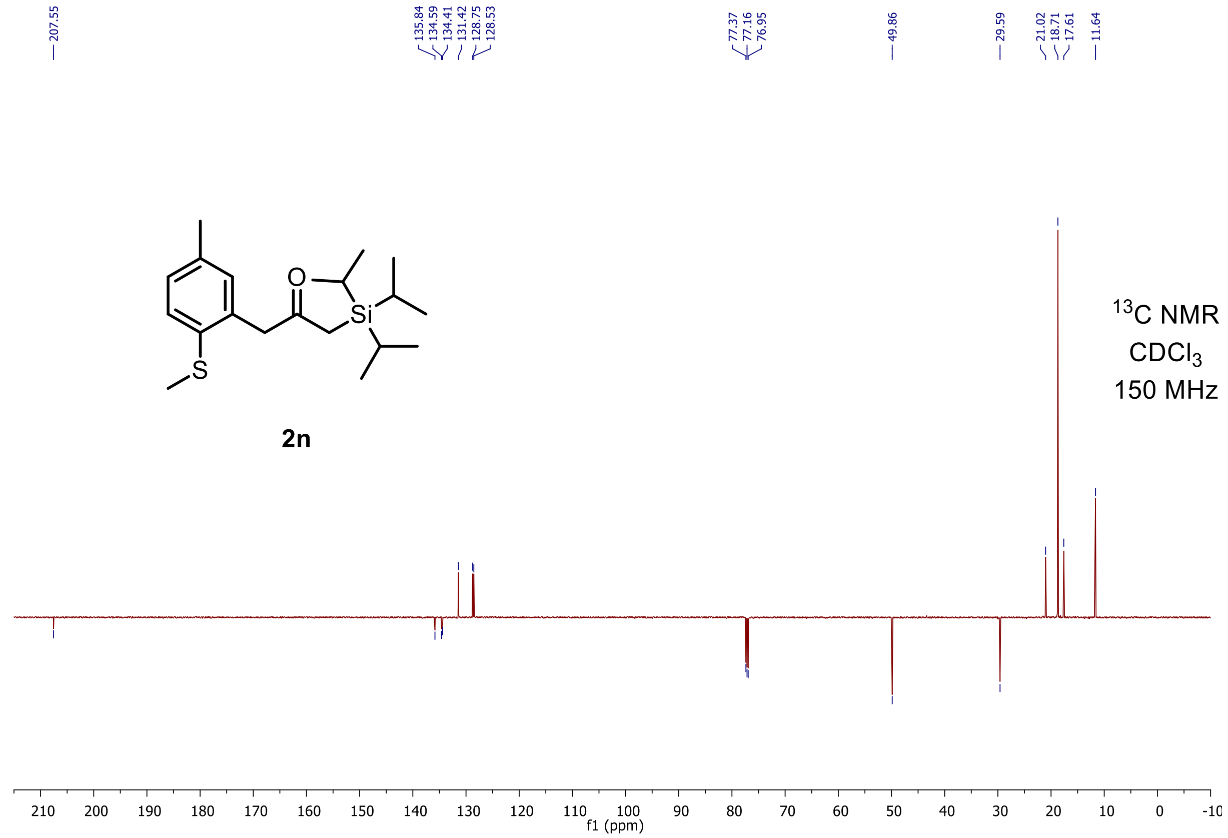
207.55
135.84
134.59
134.41
131.42
128.75
128.53
77.37
77.16
76.95
49.86
29.59
21.02
18.71
17.61
11.64
O
Si
S
2n
13C NMR
CDCl3
150 MHz
f1 (ppm)

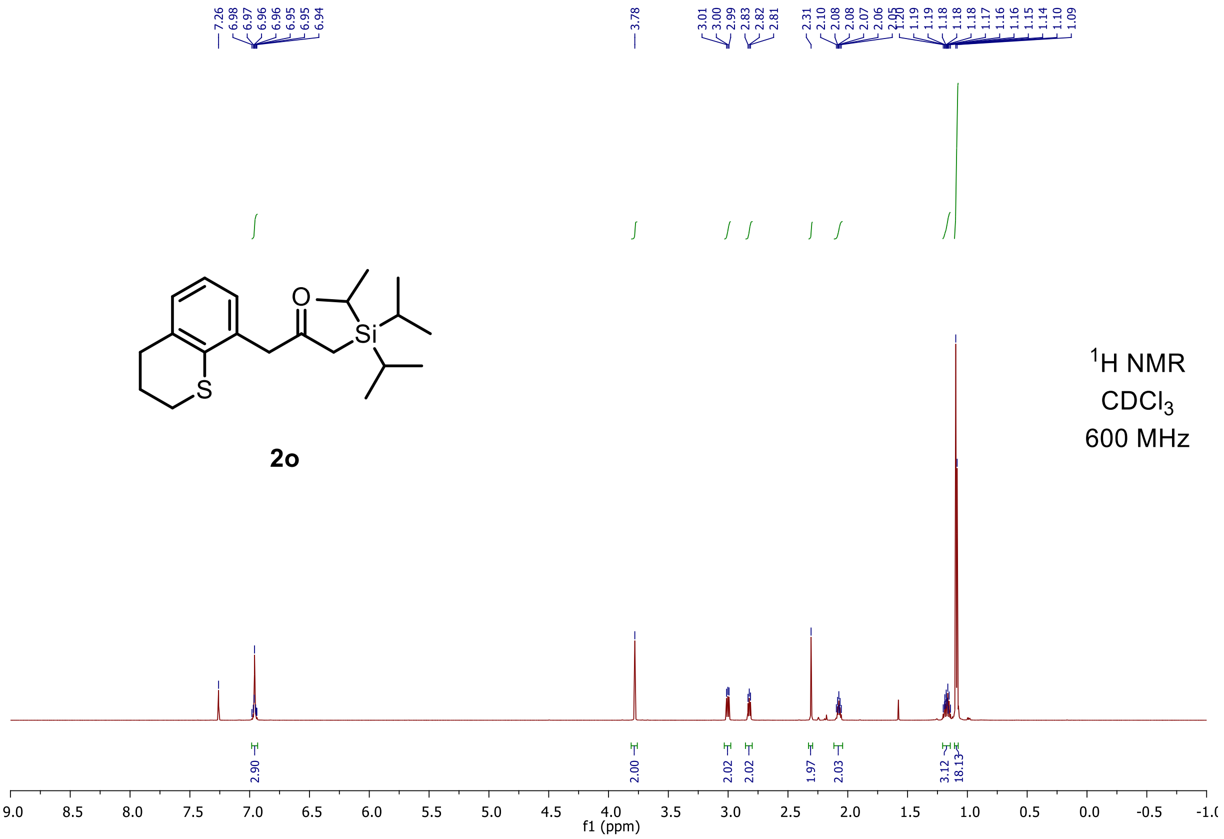
2o
^{1}H NMR
$CDCl_3$
600 MHz
f1 (ppm)

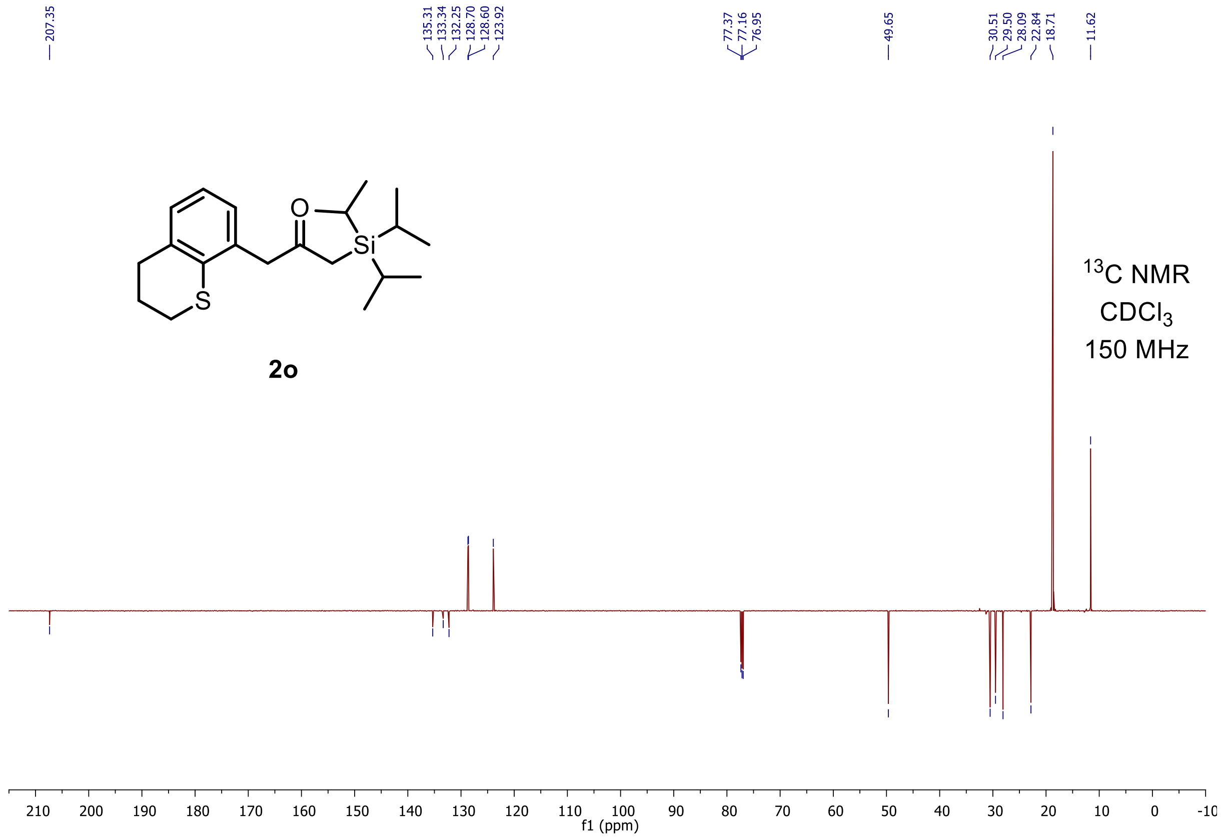
2o
^{13}C NMR
$CDCl_3$
150 MHz
f1 (ppm)

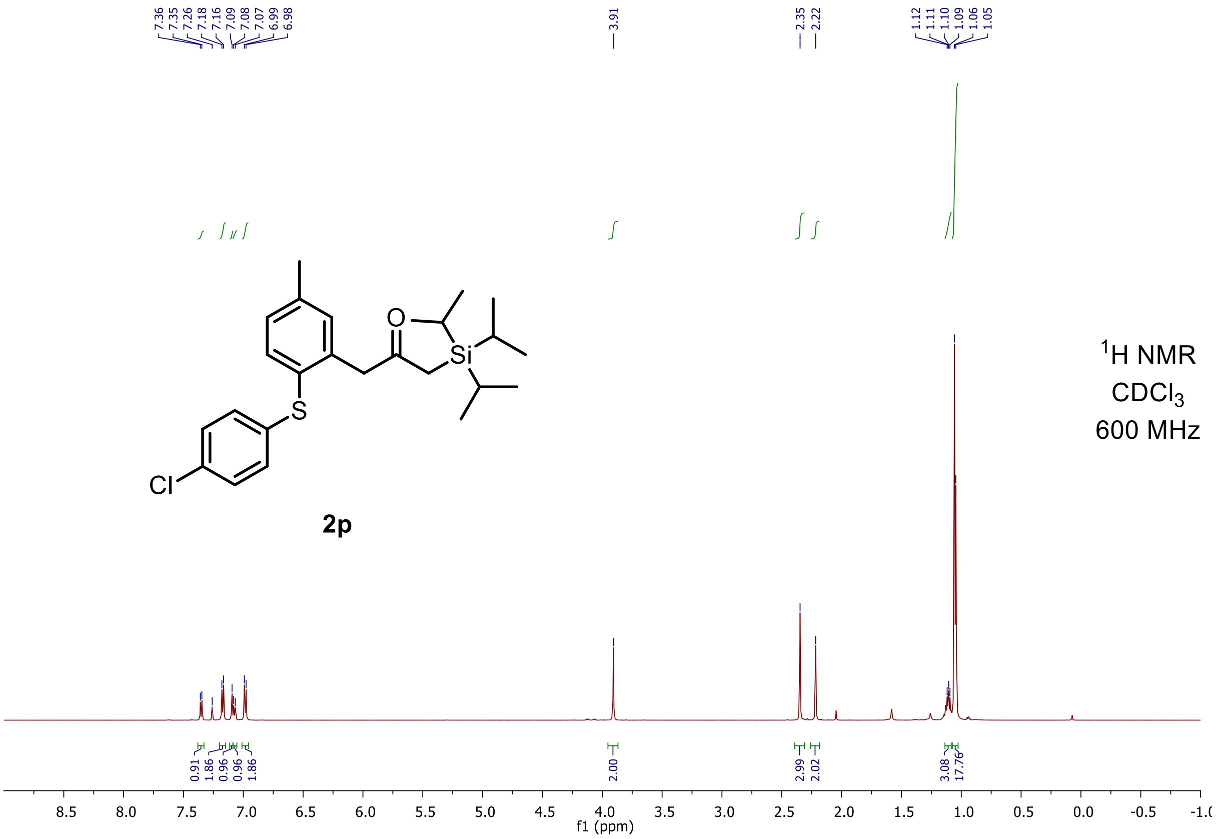

7.36
7.35
7.26
7.18
7.16
7.09
7.08
7.07
6.99
6.98
3.91
2.35
2.22
1.12
1.11
1.10
1.09
1.06
1.05
O
Si
S
Cl
2p
^{1}H NMR
$CDCl_3$
600 MHz
0.91
1.86
0.96
0.96
1.86
2.00
2.99
2.02
3.08
17.76
f1 (ppm)

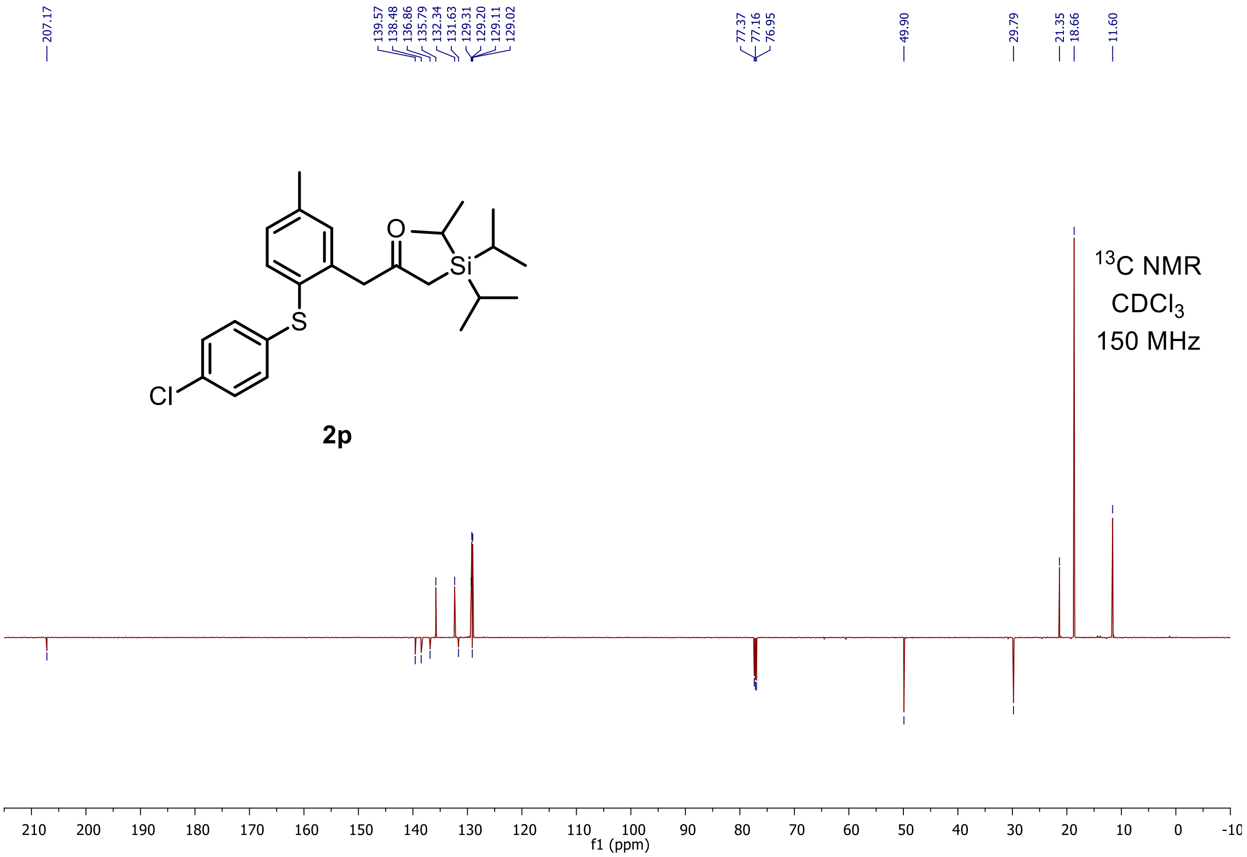

207.17
139.57
138.48
136.86
135.79
132.34
131.63
129.31
129.20
129.11
129.02
77.37
77.16
76.95
49.90
29.79
21.35
18.66
11.60
O
Si
S
Cl
2p
^{13}C NMR
$CDCl_3$
150 MHz
f1 (ppm)

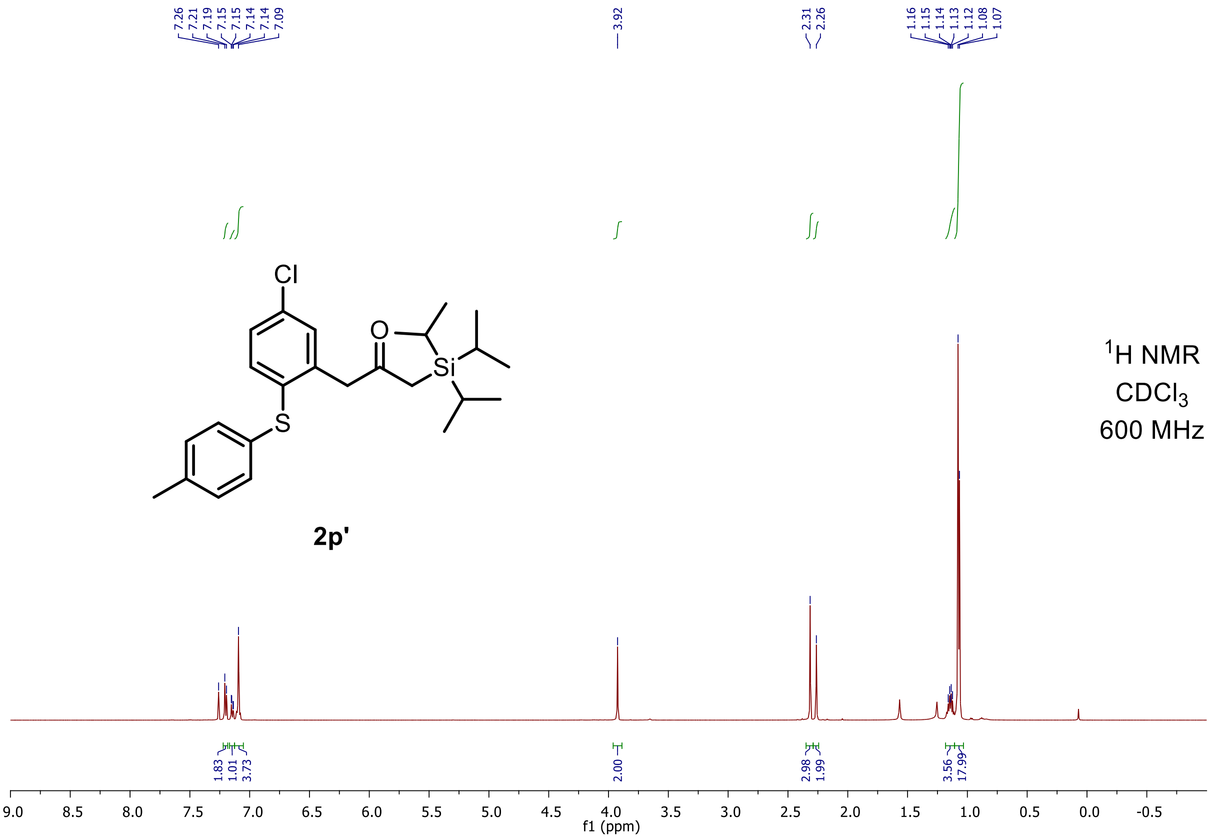
7.26
7.21
7.19
7.15
7.15
7.14
7.14
7.09
3.92
2.31
2.26
1.16
1.15
1.14
1.13
1.13
1.12
1.08
1.07
Cl
O
Si
S
2p'
^{1}H NMR
CDCl$_3$
600 MHz
1.83
1.01
3.73
2.00
2.98
1.99
3.56
17.99
f1 (ppm)

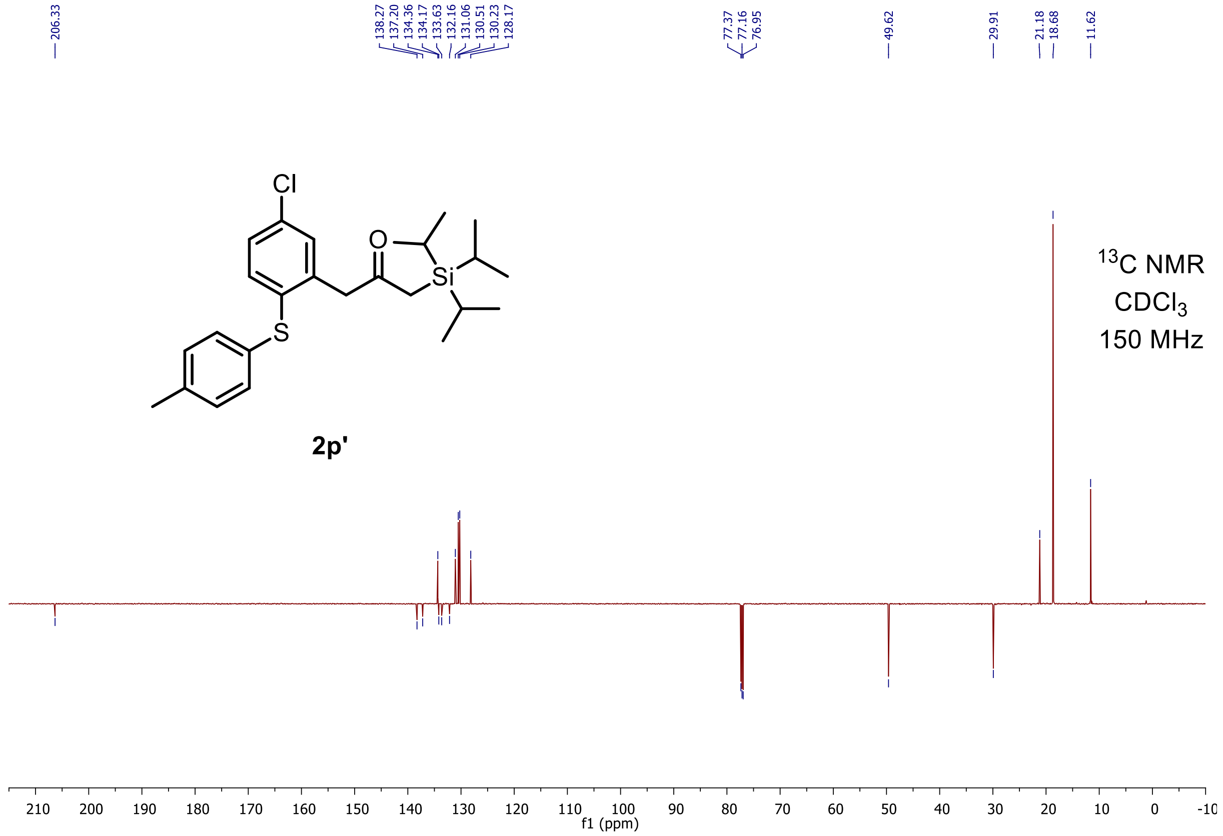
206.33
138.27
137.20
134.36
134.17
133.63
132.16
131.06
130.51
130.23
128.17
77.37
77.16
76.95
49.62
29.91
21.18
18.68
11.62
Cl
O
Si
S
2p'
^{13}C NMR
CDCl$_3$
150 MHz
f1 (ppm)

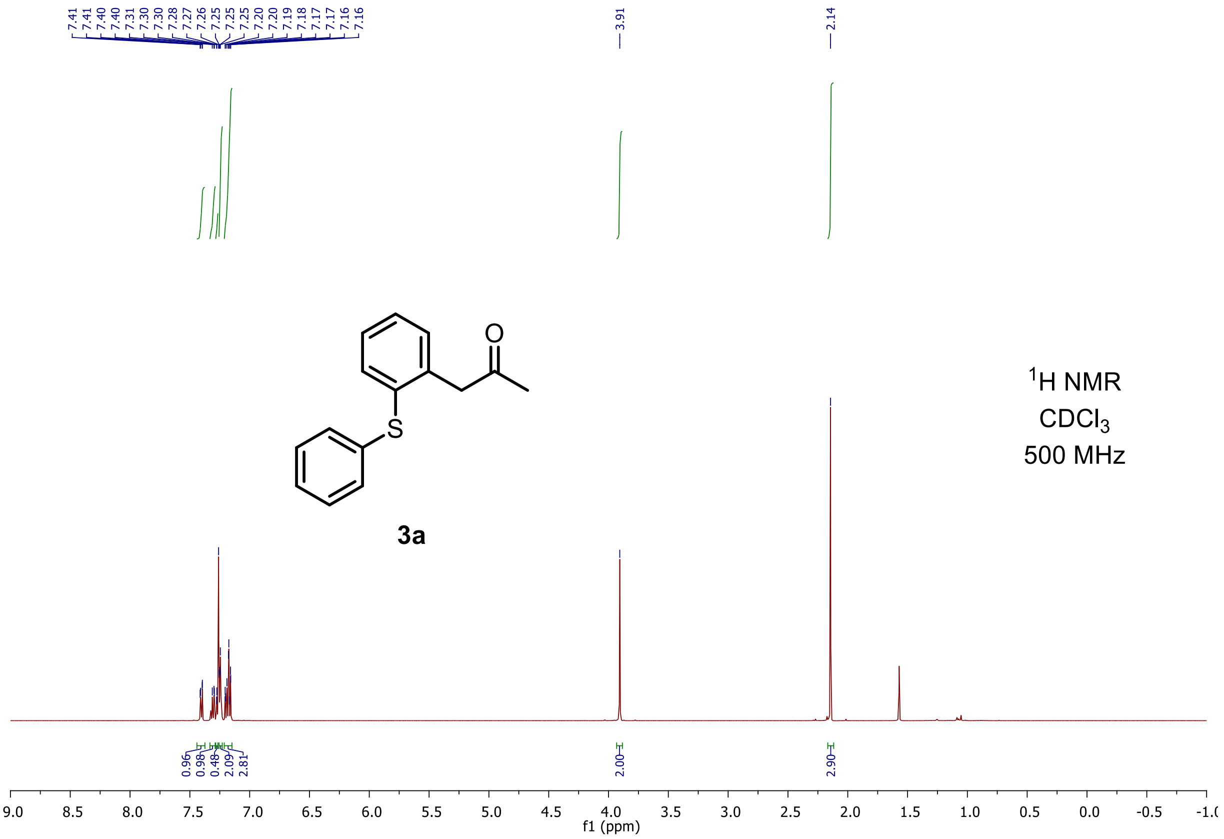
7.41
7.41
7.40
7.40
7.31
7.30
7.30
7.28
7.27
7.26
7.25
7.25
7.25
7.20
7.20
7.19
7.18
7.17
7.16
7.16
3.91
2.14
S
O
3a
1H NMR
CDCl3
500 MHz
0.96
0.98
0.48
2.09
2.81
2.00
2.90
f1 (ppm)

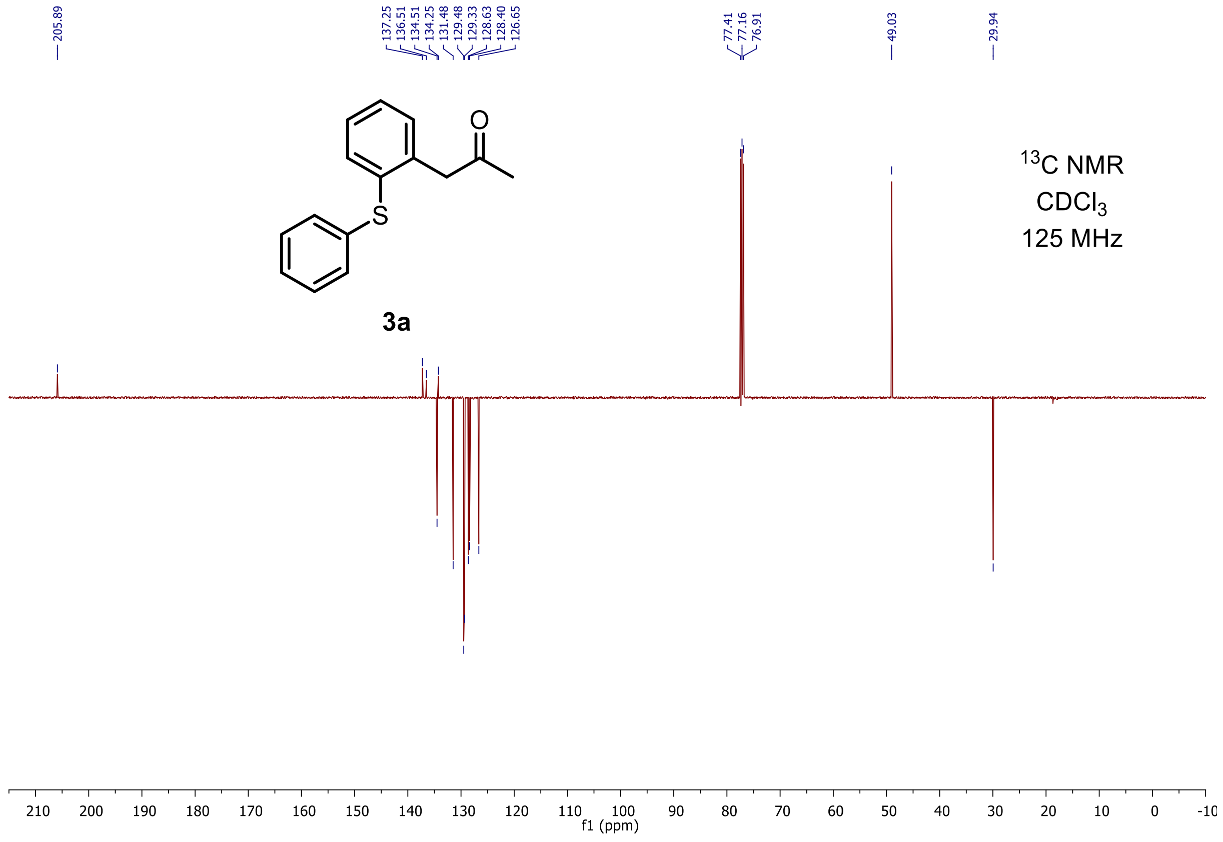
205.89
137.25
136.51
134.51
134.25
131.48
129.48
129.33
128.63
128.40
126.65
77.41
77.16
76.91
49.03
29.94
S
O
3a
13C NMR
CDCl3
125 MHz
f1 (ppm)

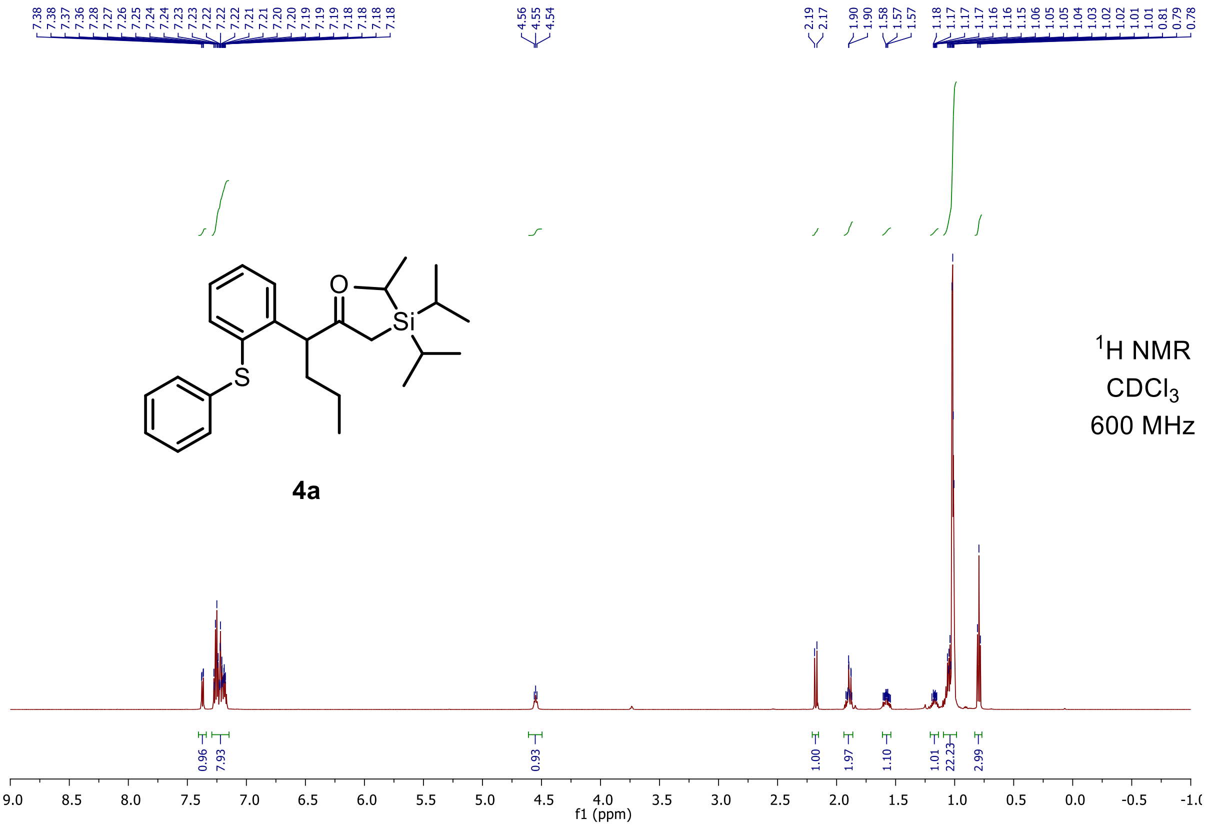
4a
1H NMR
CDCl3
600 MHz
f1 (ppm)

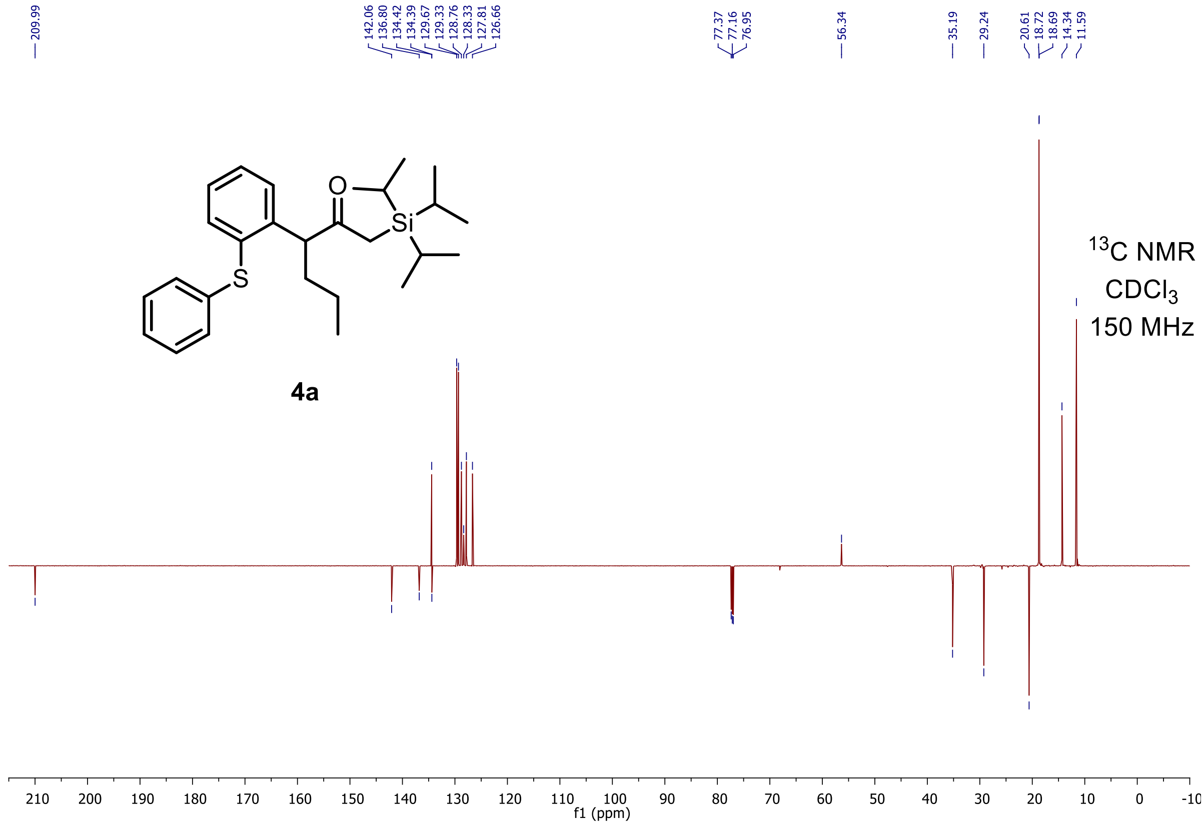
4a
13C NMR
CDCl3
150 MHz
f1 (ppm)

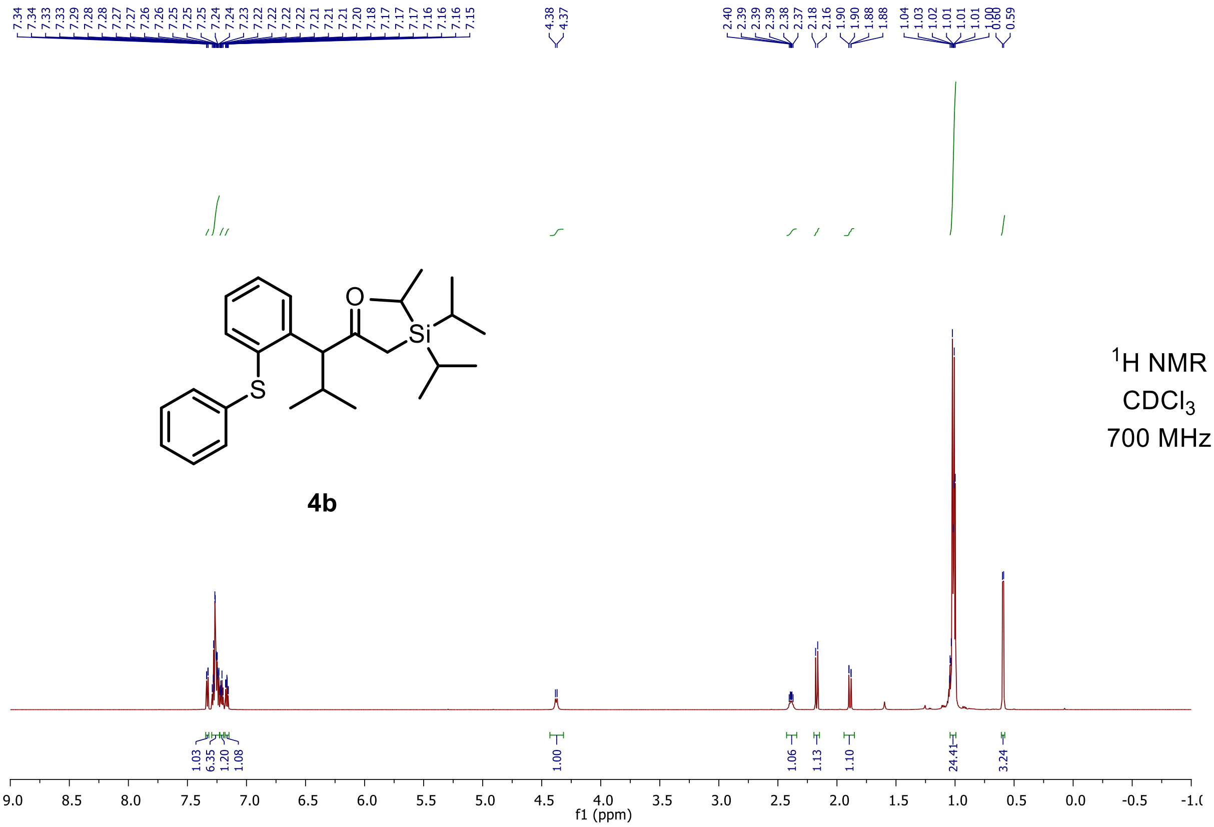
7.34 7.34 7.33 7.33 7.29 7.28 7.28 7.27 7.27 7.26 7.26 7.25 7.25 7.25 7.24 7.24 7.23 7.22 7.22 7.22 7.22 7.21 7.21 7.21 7.20 7.18 7.17 7.17 7.17 7.16 7.16 7.15
4.38 4.37
2.40 2.39 2.39 2.39 2.38 2.37 2.18 2.16 1.90 1.90 1.88 1.88
1.04 1.03 1.02 1.01 1.01 1.01 1.00 0.60 0.59
O
Si
S
4b
1H NMR
CDCl3
700 MHz
1.03 6.35 1.20 1.08
1.00
1.06 1.13 1.10
24.41
3.24
9.0 8.5 8.0 7.5 7.0 6.5 6.0 5.5 5.0 4.5 4.0 3.5 3.0 2.5 2.0 1.5 1.0 0.5 0.0 -0.5 -1.0
f1 (ppm)

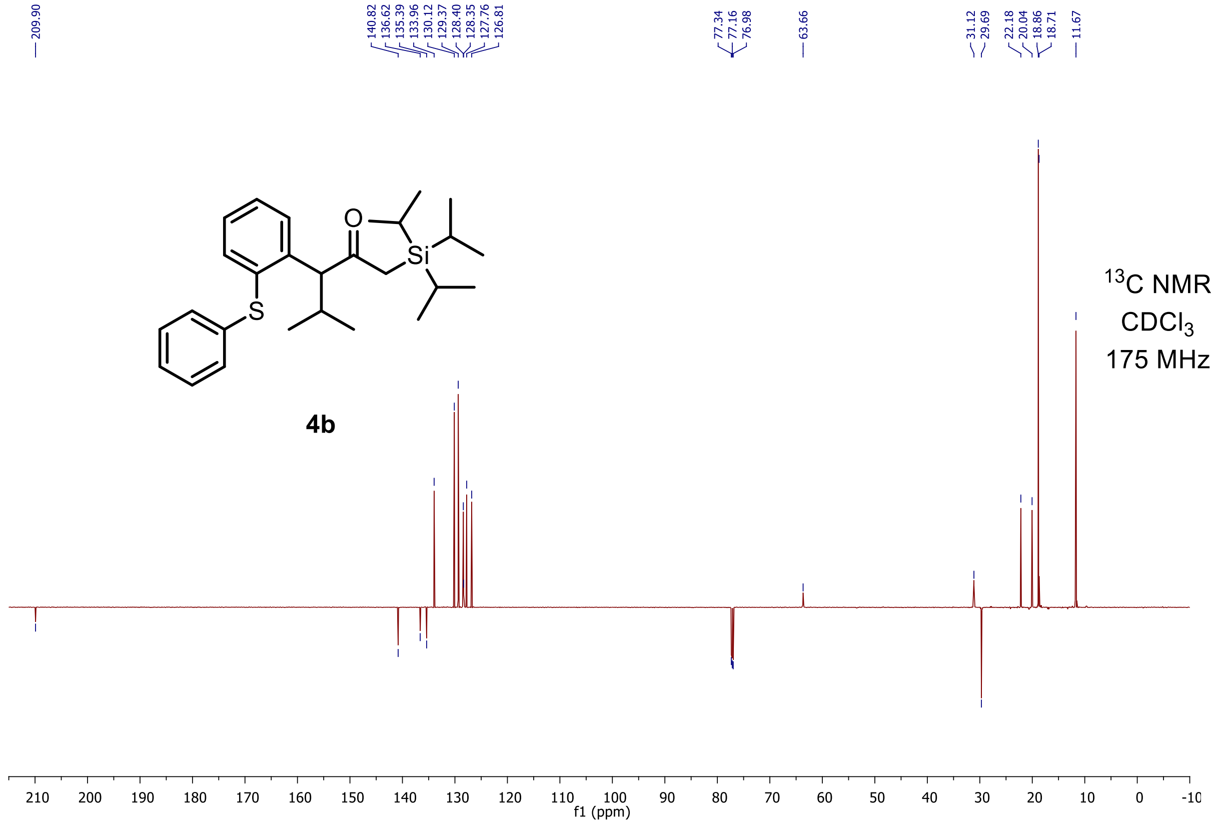
209.90
140.82 136.62 135.39 133.96 130.12 129.37 128.40 128.35 127.76 126.81
77.34 77.16 76.98
63.66
31.12 29.69
22.18 20.04 18.86 18.71
11.67
O
Si
S
4b
13C NMR
CDCl3
175 MHz
210 200 190 180 170 160 150 140 130 120 110 100 90 80 70 60 50 40 30 20 10 0 -10
f1 (ppm)

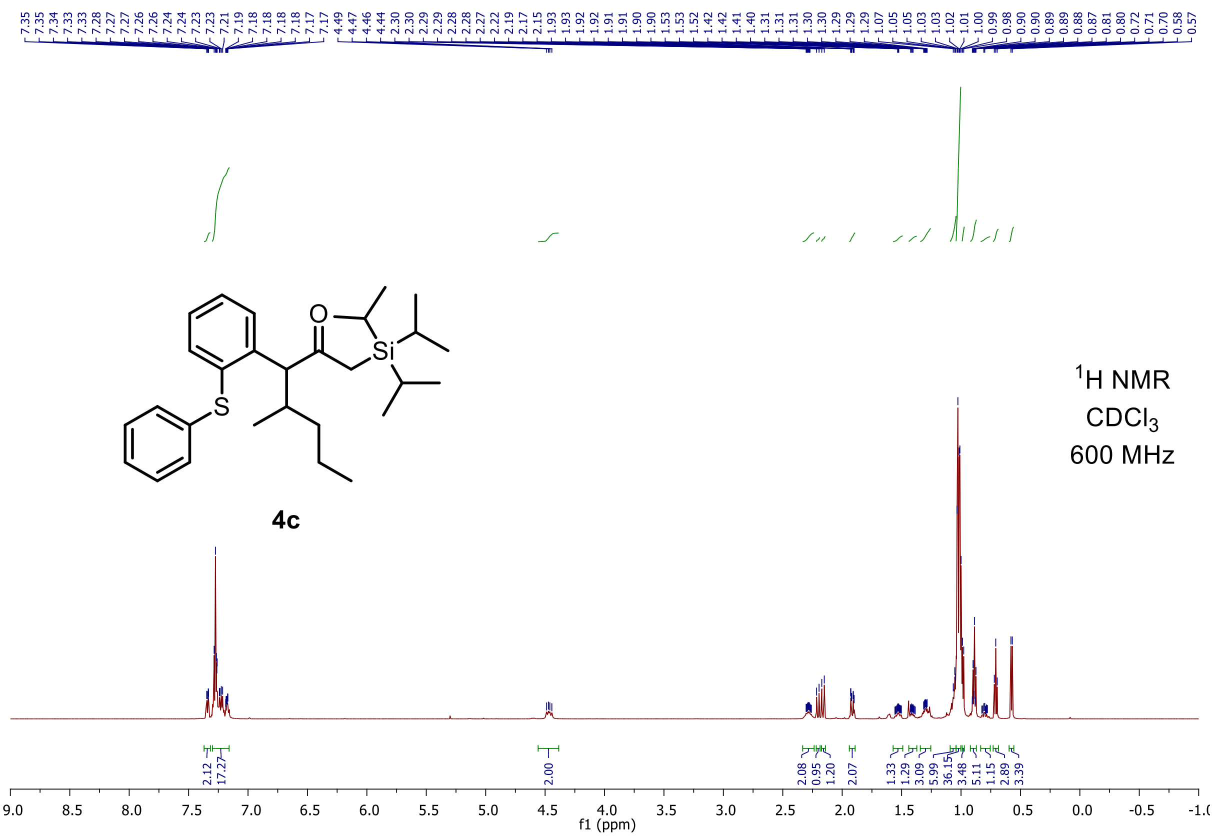
4c
1H NMR
CDCl3
600 MHz
f1 (ppm)

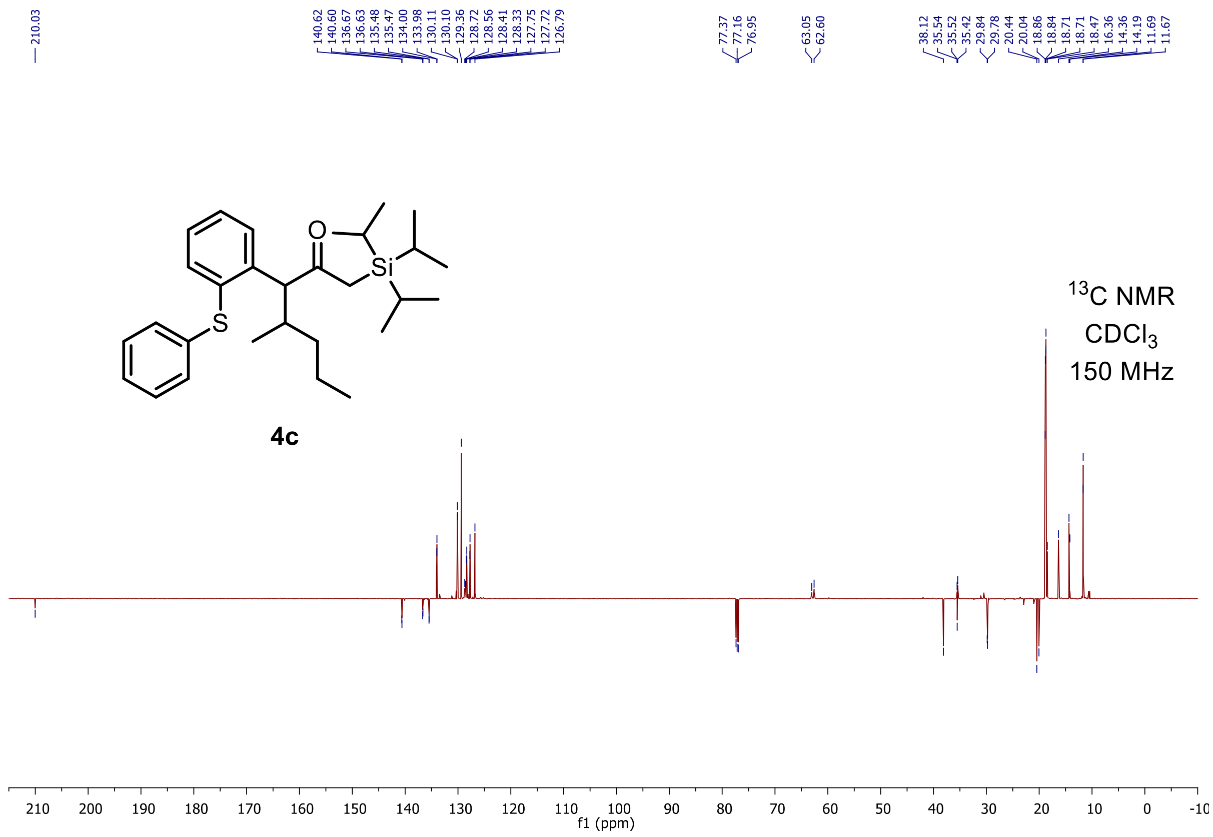
4c
13C NMR
CDCl3
150 MHz
f1 (ppm)

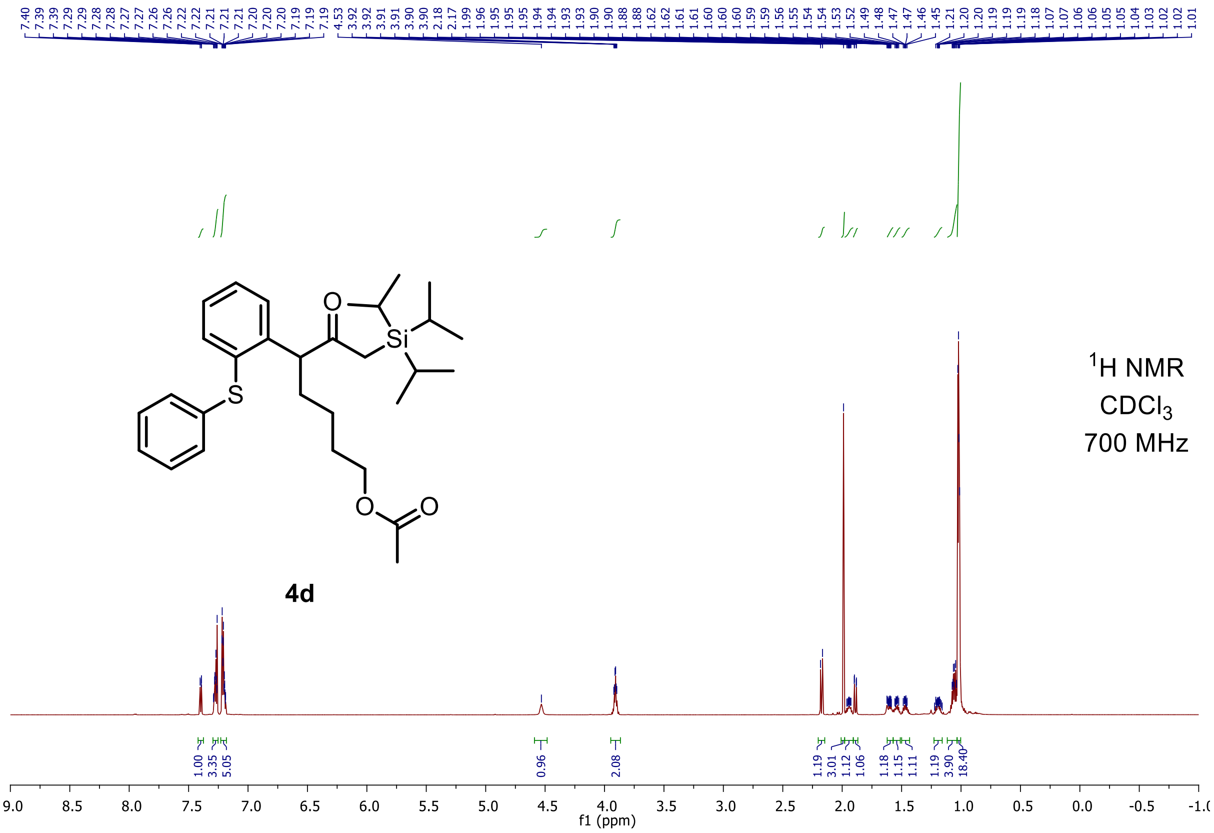
4d
^{1}H NMR
CDCl$_3$
700 MHz
f1 (ppm)

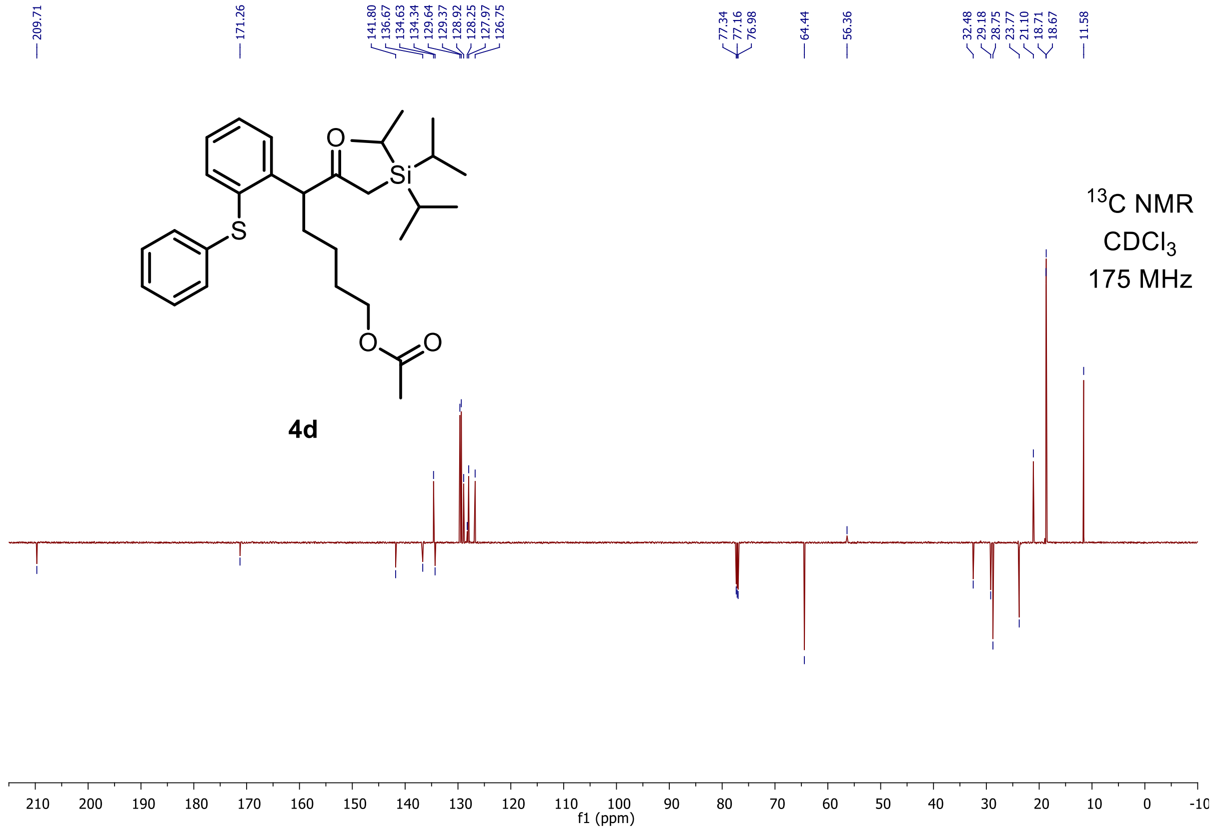
4d
^{13}C NMR
CDCl$_3$
175 MHz
f1 (ppm)

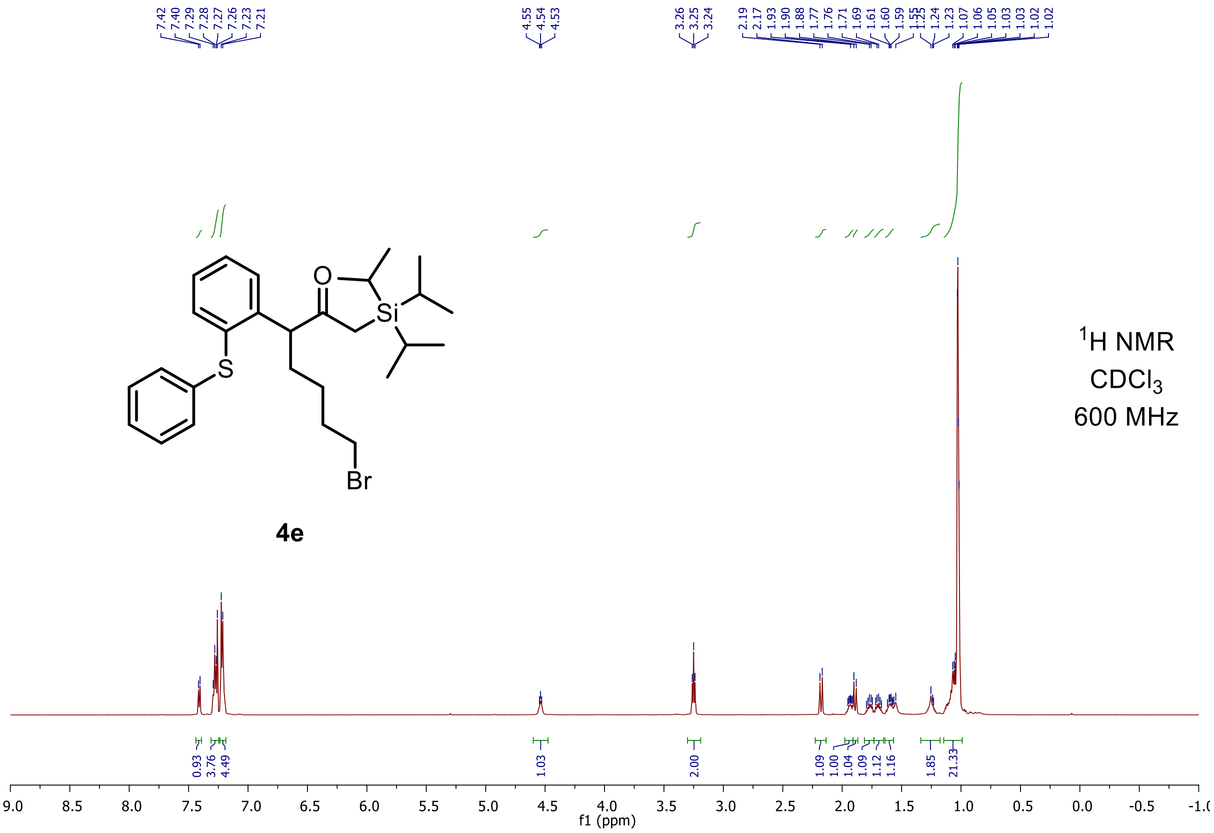

O
Si
S
Br
4e
^{1}H NMR
CDCl$_3$
600 MHz
f1 (ppm)

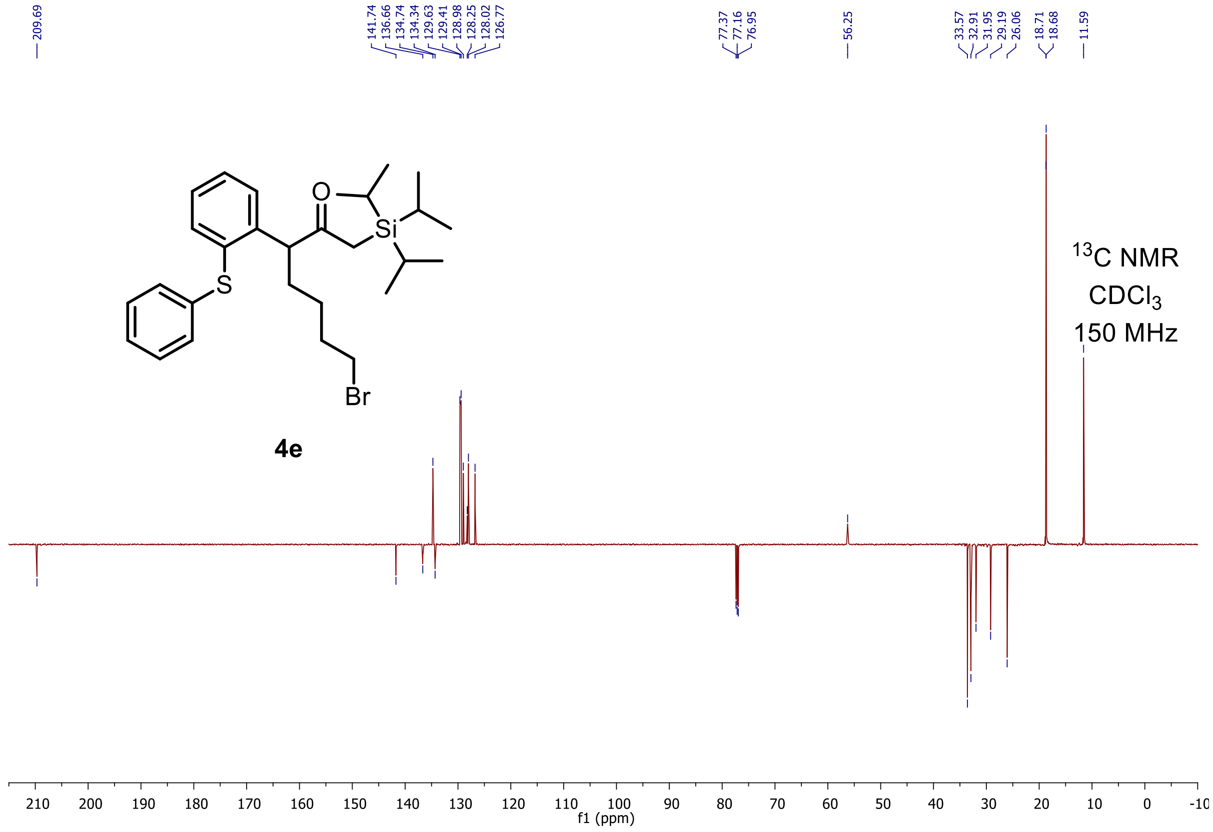

O
Si
S
Br
4e
^{13}C NMR
CDCl$_3$
150 MHz
f1 (ppm)

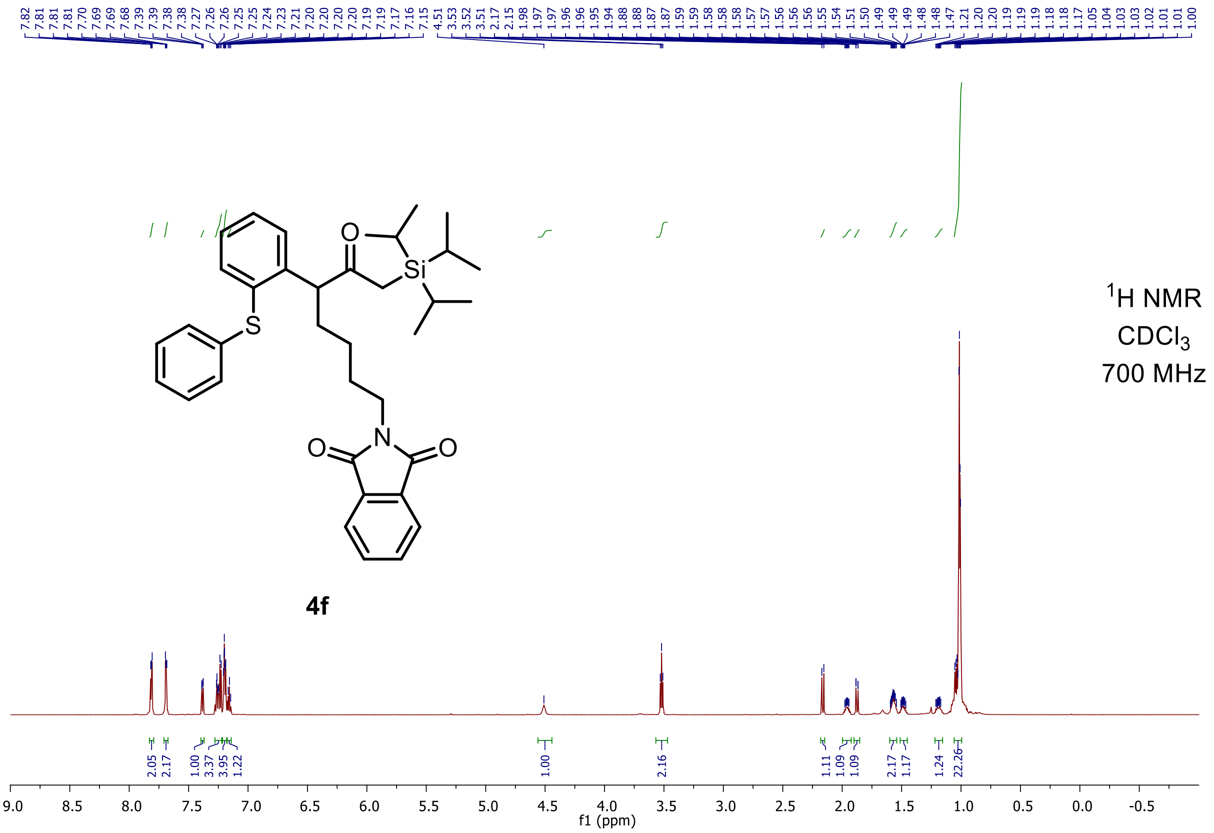
7.82 7.81 7.81 7.81 7.70 7.69 7.69 7.68 7.39 7.39 7.38 7.38 7.27 7.26 7.26 7.25 7.25 7.24 7.23 7.21 7.20 7.20 7.20 7.20 7.19 7.19 7.19 7.17 7.16 7.15 4.51 3.53 3.52 3.51 2.17 2.15 1.98 1.97 1.97 1.96 1.96 1.95 1.94 1.88 1.88 1.87 1.59 1.59 1.58 1.58 1.58 1.57 1.57 1.56 1.56 1.56 1.55 1.54 1.51 1.50 1.49 1.49 1.49 1.48 1.47 1.21 1.20 1.19 1.19 1.19 1.18 1.18 1.17 1.05 1.04 1.03 1.03 1.02 1.01 1.01 1.00
4f
^{1}H NMR
$CDCl_3$
700 MHz
2.05 2.17 1.00 3.37 3.95 1.22 1.00 2.16 1.11 1.09 1.09 2.17 1.17 1.24 22.26
9.0 8.5 8.0 7.5 7.0 6.5 6.0 5.5 5.0 4.5 4.0 3.5 3.0 2.5 2.0 1.5 1.0 0.5 0.0 -0.5
f1 (ppm)

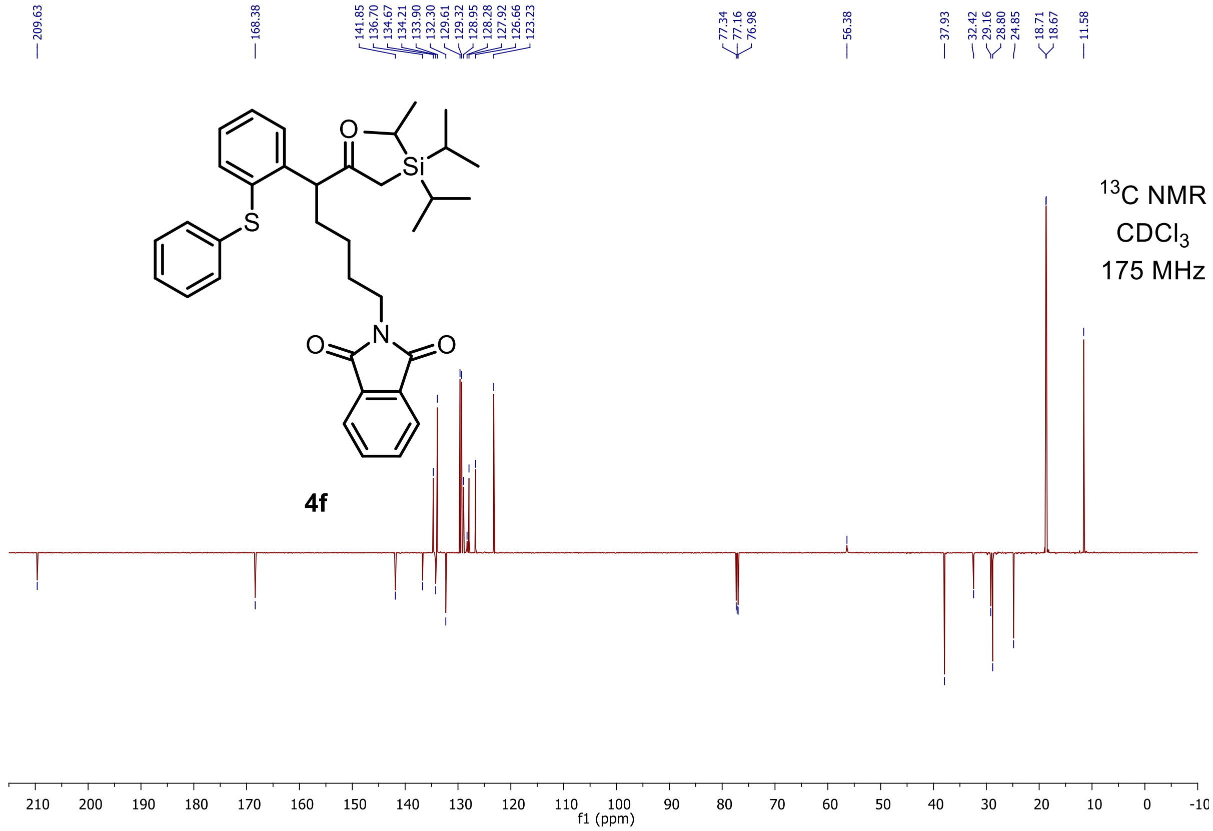
209.63
168.38
141.85 136.70 134.67 134.21 133.90 132.30 129.61 129.32 128.95 128.28 127.92 126.66 123.23
77.34 77.16 76.98
56.38
37.93
32.42 29.16 28.80 24.85
18.71 18.67
11.58
4f
^{13}C NMR
$CDCl_3$
175 MHz
210 200 190 180 170 160 150 140 130 120 110 100 90 80 70 60 50 40 30 20 10 0 -10
f1 (ppm)

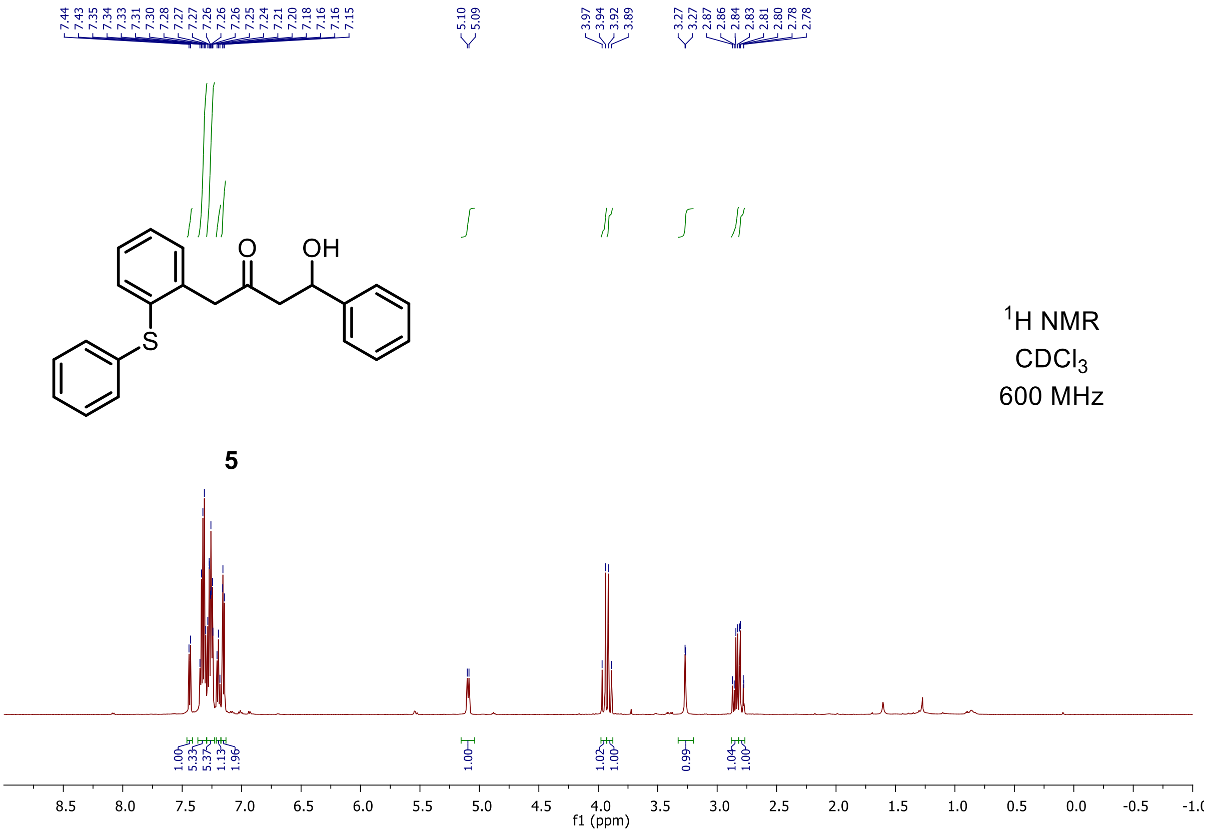
7.44
7.43
7.35
7.34
7.33
7.31
7.30
7.28
7.27
7.27
7.26
7.26
7.26
7.25
7.24
7.21
7.20
7.18
7.16
7.15
5.10
5.09
3.97
3.94
3.92
3.89
3.27
3.27
2.87
2.86
2.84
2.83
2.81
2.80
2.78
2.78
O
OH
S
5
1H NMR
CDCl3
600 MHz
1.00
5.33
5.37
1.13
1.96
1.00
1.02
1.00
0.99
1.04
1.00
f1 (ppm)

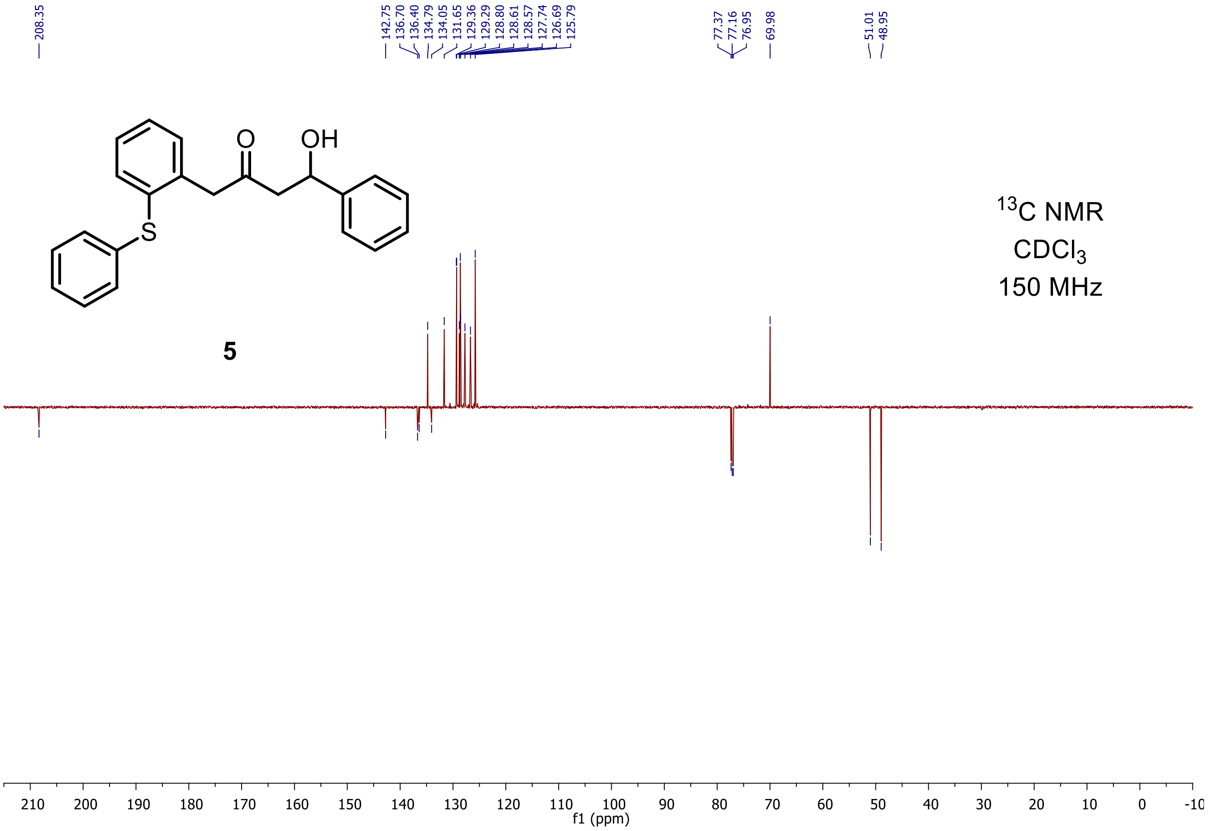
208.35
142.75
136.70
136.40
134.79
134.05
131.65
129.36
129.29
128.80
128.61
128.57
127.74
126.69
125.79
77.37
77.16
76.95
69.98
51.01
48.95
O
OH
S
5
13C NMR
CDCl3
150 MHz
f1 (ppm)

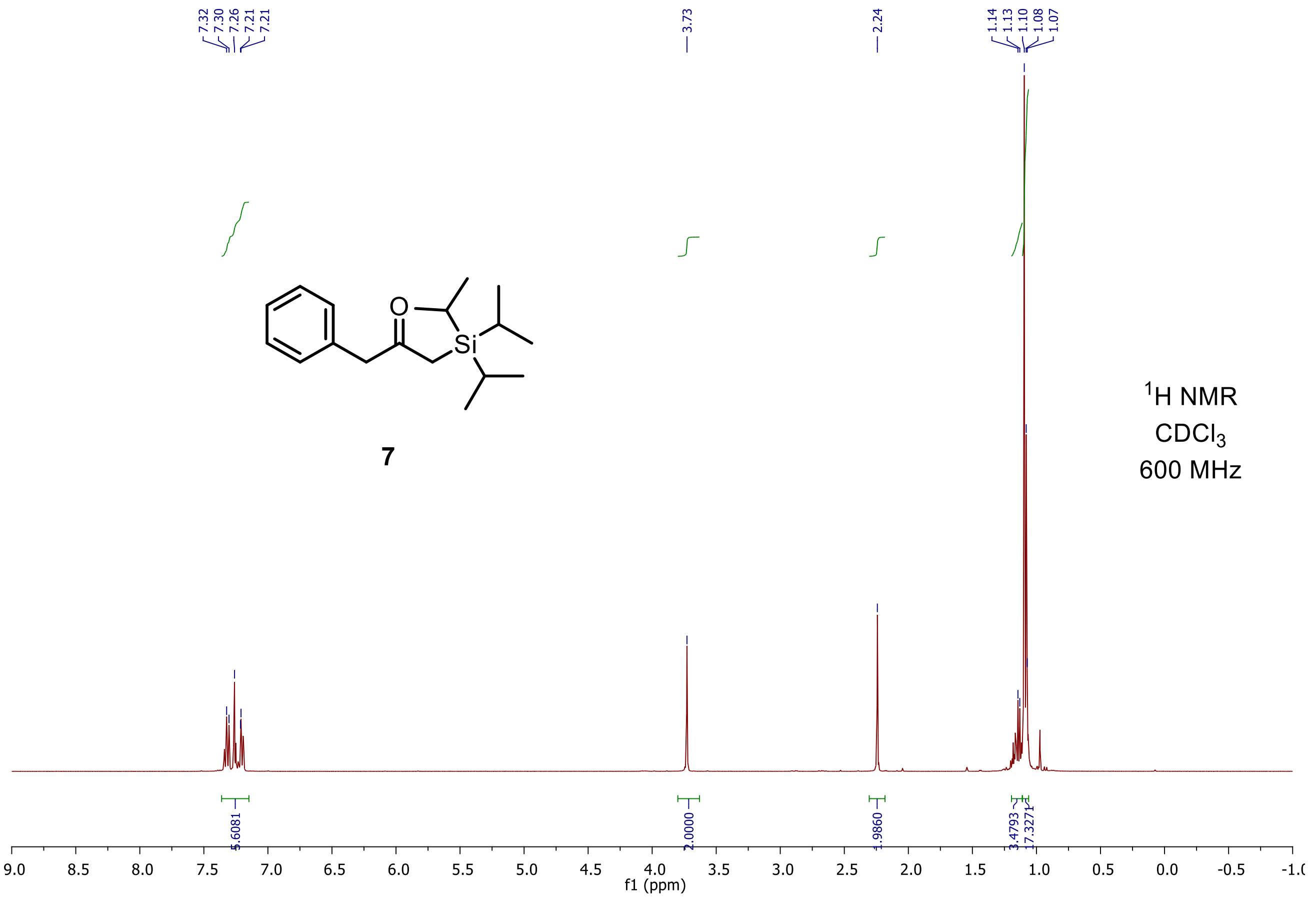
7.32
7.30
7.26
7.21
7.21
3.73
2.24
1.14
1.13
1.10
1.08
1.07
O
Si
7
^{1}H NMR
$CDCl_3$
600 MHz
5.6081
2.0000
1.9860
3.4793
17.3271
f1 (ppm)

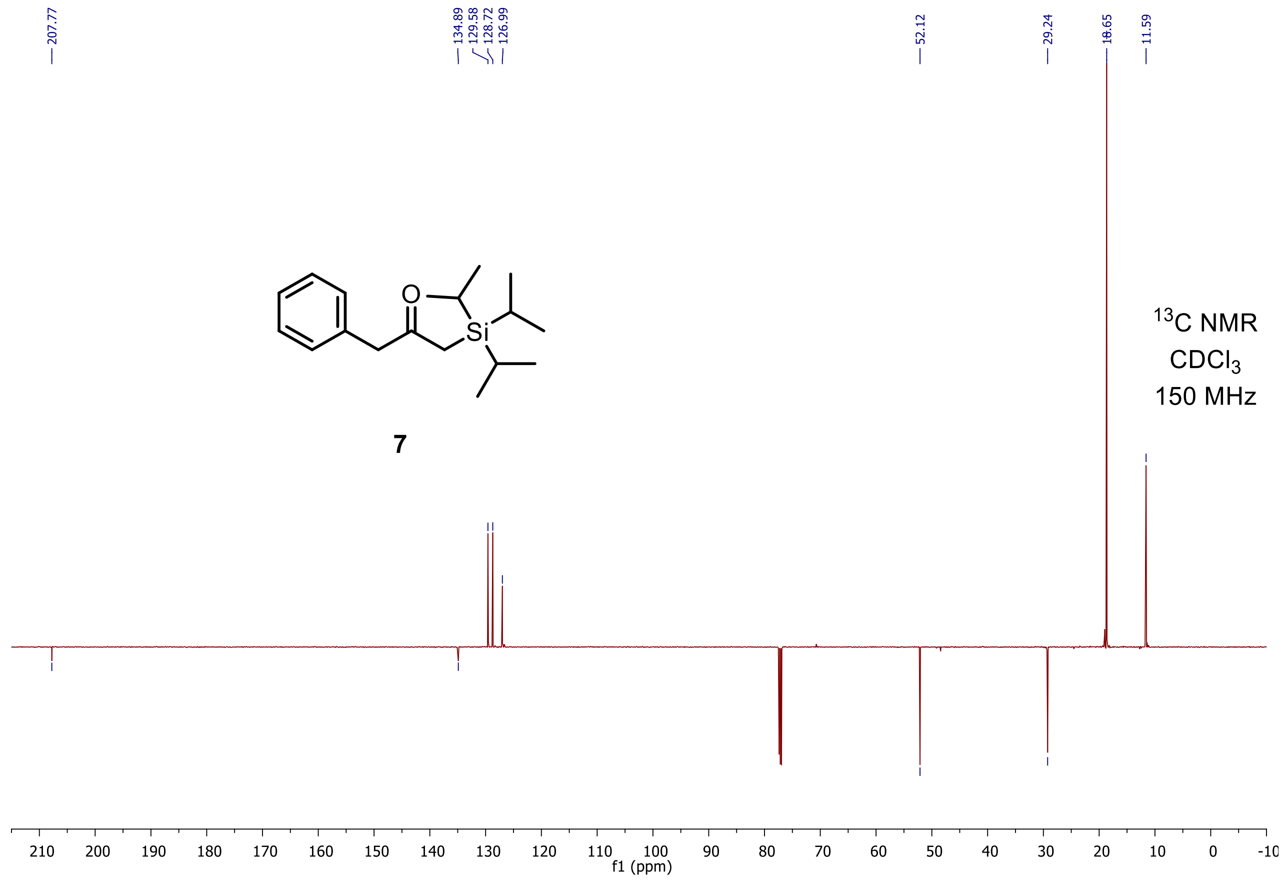
207.77
134.89
129.58
128.72
126.99
52.12
29.24
18.65
11.59
O
Si
7
^{13}C NMR
$CDCl_3$
150 MHz
f1 (ppm)

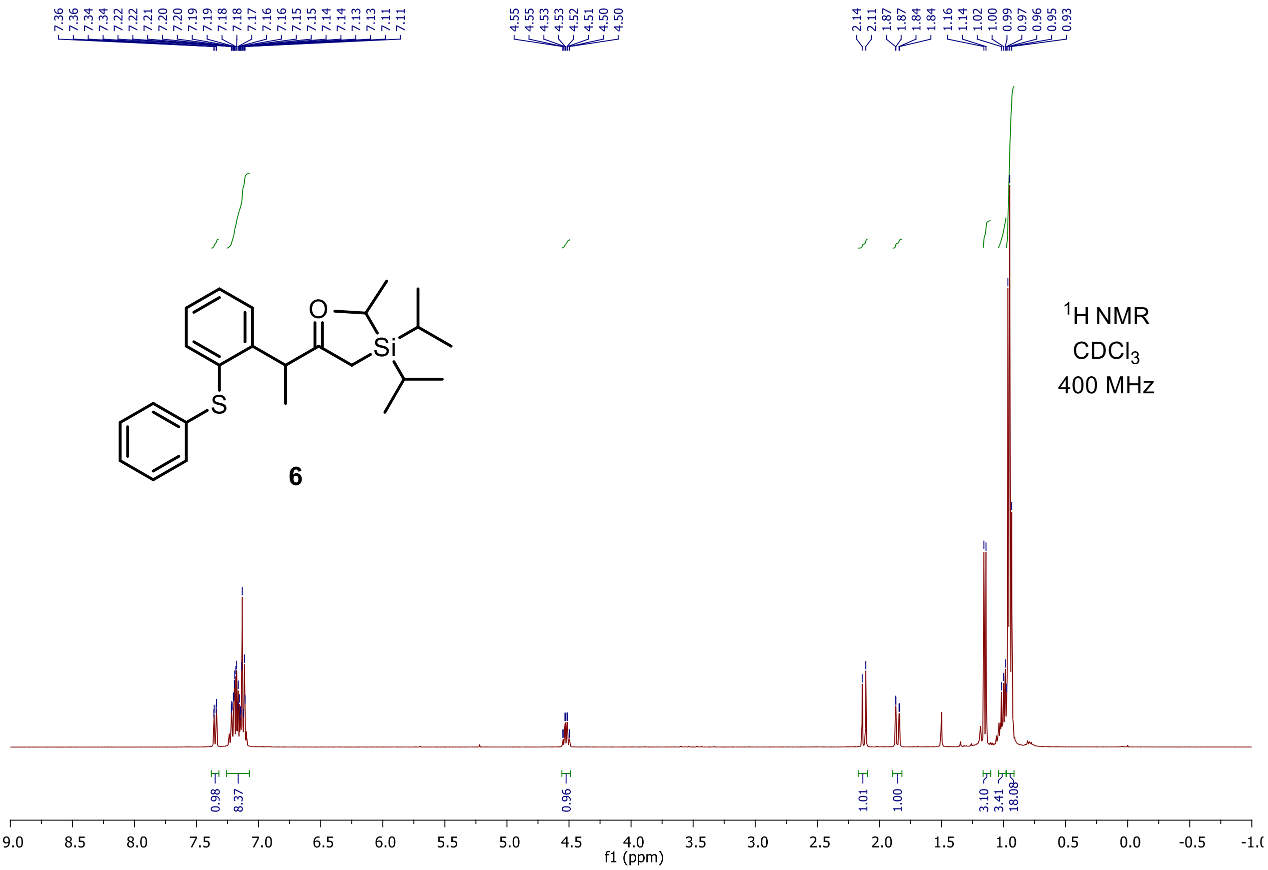
6
1H NMR
CDCl3
400 MHz
f1 (ppm)

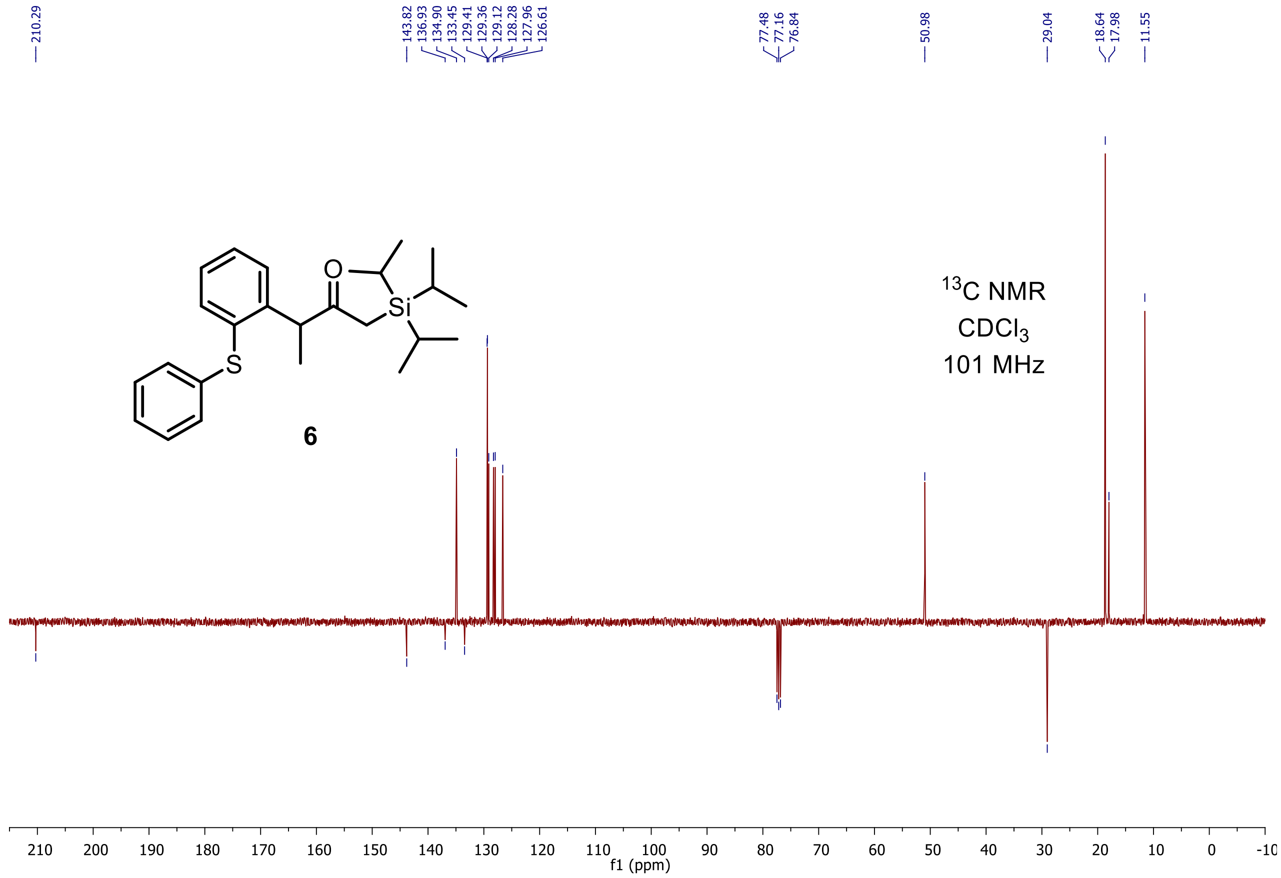
6
13C NMR
CDCl3
101 MHz
f1 (ppm)

References

[1] J.-H. Chun, C. L. Morse, F. T. Chin, V. W. Pike, *Chem. Commun.* **2013**, *49*, 2151.

[2] X. Li, Y. Sun, X. Huang, L. Zhang, L. Kong, B. Peng, *Org. Lett.* **2017**, *19*, 838.

[3] D. Kaiser, L. F. Veiros, N. Maulide, *Chem. – Eur. J.* **2016**, *22*, 4727.

[4] J. Drabowicz, P. Łyżwa, M. Popielarczyk, M. Mikołajczyk, *Synthesis* **1990**, *1990*, 937.

[5] B. Wang, Y. Liu, C. Lin, Y. Xu, Z. Liu, Y. Zhang, *Org. Lett.* **2014**, *16*, 4574.

[6] B. Rama Raju, S. Sarkar, U. Chandramoulali Reddy, A. K. Saikia, *J. Mol. Catal. Chem.* **2009**, *308*, 169.

[7] S. Gan, J. Yin, Y. Yao, Y. Liu, D. Chang, D. Zhu, L. Shi, *Org. Biomol. Chem.* **2017**, *15*, 2647.

[8] P. A. Procopiou, V. J. Barrett, K. Biggadike, P. R. Butchers, A. Craven, A. J. Ford, S. B. Guntrip, D. S. Holmes, S. C. Hughes, A. E. Jones, et al., *J. Med. Chem.* **2014**, *57*, 159.

[9] F. J. Devlin, P. J. Stephens, P. Scafato, S. Superchi, C. Rosini, *Tetrahedron Asymmetry* **2001**, *12*, 1551.

[10] (a) J. Pornet, *J. Organomet. Chem.* **1988**, *340*, 273-282. (b) M. Puriņš, A. Mishnev, M. Turks, *J. Org. Chem.* **2019**, *84*, 3595.

[11] L. L. Baldassari, A. C. Mantovani, Senoner S., B. Maryasin, N. Maulide, D. S. Lüdtke, *Org. Lett.* **2018**, *20*, 5881.
